# Loss of TIMP3 underlies diabetic nephropathy via FoxO1/STAT1 interplay

**DOI:** 10.1002/emmm.201201475

**Published:** 2013-02-12

**Authors:** Loredana Fiorentino, Michele Cavalera, Stefano Menini, Valentina Marchetti, Maria Mavilio, Marta Fabrizi, Francesca Conserva, Viviana Casagrande, Rossella Menghini, Paola Pontrelli, Ivan Arisi, Mara D'Onofrio, Davide Lauro, Rama Khokha, Domenico Accili, Giuseppe Pugliese, Loreto Gesualdo, Renato Lauro, Massimo Federici

**Affiliations:** 1 Department of Systems Medicine, University of Rome Tor VergataRome, Italy; 2 Department of Clinical and Molecular Medicine, University of Rome La SapienzaRome, Italy; 3 Department of Emergency and Organ Transplantation, University of BariBari, Italy; 4 Genomics Unit, European Brain Research InstituteRome, Italy; 5 Ontario Cancer Institute, University of TorontoToronto, Ontario, Canada; 6 Department of Medicine, Columbia UniversityNY, USA

**Keywords:** autophagy, diabetic nephropathy, FoxO1, STAT1, TIMP3

## Abstract

ADAM17 and its inhibitor TIMP3 are involved in nephropathy, but their role in diabetic kidney disease (DKD) is unclear. Diabetic *Timp3*^*−/−*^ mice showed increased albuminuria, increased membrane thickness and mesangial expansion. Microarray profiling uncovered a significant reduction of *Foxo1* expression in diabetic *Timp3*^*−/−*^ mice compared to WT, along with FoxO1 target genes involved in autophagy, while STAT1, a repressor of FoxO1 transcription, was increased. Re-expression of Timp3 in *Timp3*^*−/−*^ mesangial cells rescued the expression of Foxo1 and its targets, and decreased STAT1 expression to control levels; abolishing STAT1 expression led to a rescue of FoxO1, evoking a role of STAT1 in linking *Timp3* deficiency to FoxO1. Studies on kidney biopsies from patients with diabetic nephropathy confirmed a significant reduction in TIMP3, FoxO1 and FoxO1 target genes involved in autophagy compared to controls, while STAT1 expression was strongly increased.

Our study suggests that loss of TIMP3 is a hallmark of DKD in human and mouse models and designates TIMP3 as a new possible therapeutic target for diabetic nephropathy.

## INTRODUCTION

Diabetic kidney disease (DKD) is one of the most severe complications associated with both type 1 and type 2 diabetes, developing in about one-third of diabetic patients (Groop et al, 2009). DKD is characterized by albuminuria, glomerulosclerosis and progressive loss of renal function. Poor glycaemic control and elevated systolic blood pressure exacerbate proteinuria and renal injury that may culminate in end-stage renal disease (Lane et al, 1990; Mogensen et al, 1983). Current therapies for DKD, such as blood glucose control, angiotensin II (ATII) receptors blockers and ACE inhibitors, slow down, but do not halt, the progression of this pathology (Ruggenenti et al, 2010). Recently, a cross-talk between ATII and the epidermal growth factor receptor (EGFR) has been shown to play a pivotal role in stimulating the development of renal lesions. ATII also causes redistribution of the metalloprotease ADAM17 to the apical membrane of renal tubules (Lautrette et al, 2005).

ADAMs are transmembrane proteins with shedding activity acting on a variety of substrates localized in the plasma membrane to generate inflammatory, growth, migration and metabolic signals. These enzymes belong to the metalloproteinase class of enzymes which also comprise matrix metalloproteinases (MMPs) known for the continuous remodelling of the extracellular matrix and cleavage of cell surface proteins (Dreymueller et al, 2012). Recent data suggest a role for MMPs in a number of acute and chronic renal disorders (Catania et al, 2007).

ADAM17, also known as TNF-α converting enzyme (TACE), mediates the shedding of TNF-α and its receptors (TNFRI and II), adhesion molecules (l-selectin, VCAM), and several EGFR ligands, such as amphiregulin, TGF-α and heparin-binding EGF-like growth factor (HB-EGF; Blobel, 2000, 2005). This latter class of molecules have been implicated in the development of renal inflammatory and fibrotic lesions in mice (Bollee et al, 2011). Recently, it has been shown that elevated serum concentrations of soluble TNFRI and II are strong predictor of early renal function loss either in type 1 and type 2 diabetes (Gohda et al, 2012; Niewczas et al, 2012). ADAM17 is also involved in the cleavage of Notch in the plasma membrane to generate the Notch intra-cellular domain (NICD), which then moves to the nucleus to regulate gene expression (Murthy et al, 2012). The Notch pathway is necessary for glomerular and proximal tubular development, and its alteration is involved in DKD (Niranjan et al, 2008).

The proteolytic activity of ADAMs and MMPs is finely regulated by endogenous inhibitors called TIMPs (tissue inhibitor of metalloproteinase, 1/2/3/4), with TIMP3 being effective on most ADAMs (Mohammed et al, 2003). Loss of TIMP3, the only known physiological inhibitors of ADAM17, is associated with age-related renal fibrosis and tubulointerstitial fibrosis (Kawamoto et al, 2006; Kassiri et al, 2009), which are important prognostic marker in a wide variety of kidney diseases. TIMP3 was also shown to block the binding of VEGF to VEGF receptor-2 and inhibit downstream signalling and angiogenesis (Qi et al, 2003), and evidence is emerging that VEGF plays a crucial role in maintaining renal homeostasis, as altered (increased or decreased) expression of VEGF leads to glomerular dysfunction and proteinuria (Rask-Madsen & King, 2010). Moreover, Notch and VEGF pathways interact in diabetic podocytes to drive the development of DKD (Lin et al, 2010).

In our study we investigated whether a loss of TIMP3 contributes to the onset and progression of DKD in mouse models. We found that TIMP3 deficiency decreases FoxO1 functions, through an interplay with STAT1, particularly relating to protection from oxidative stress and autophagy. A role for TIMP3/FoxO1 axis in regulation of the autophagy process was investigated in cellular models. We identify similar changes in expression of human TIMP3 and FoxO1 in renal biopsies from patients with diabetic nephropathy. Our findings highlight TIMP3 as a possible new therapeutic target for DKD.

## RESULTS

### Expression of TIMPs and ADAMs in STZ-induced diabetic mice

**Figure 1:**
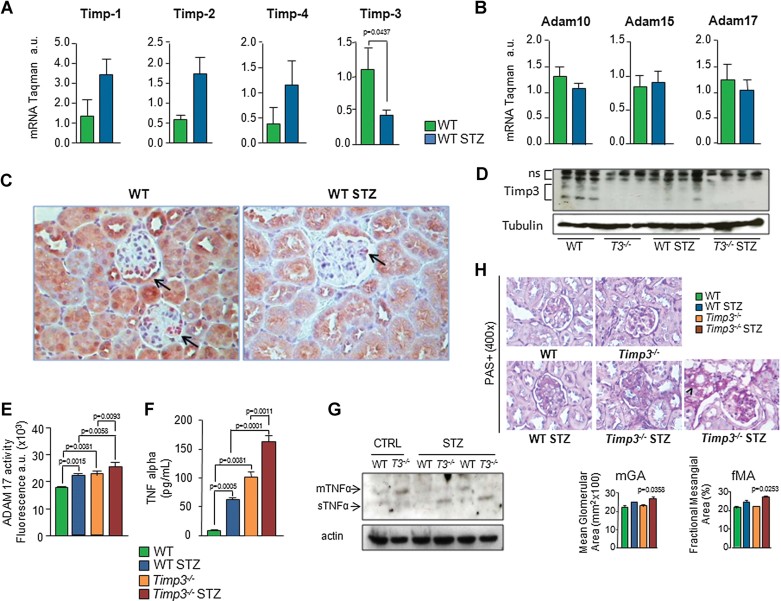
Expression of TIMPs and ADAMs in diabetic mice Levels of Timp family proteins expression were analysed by real-time PCR on mRNA extracted from kidneys of diabetic mice and normoglycemic littermates (*n* = 6, Student's *t*-test).Real-time PCR analysis of Adam10, 15 and 17 expression in diabetic and healthy kidneys (*n* = 6).Immunohistochemical staining of kidney sections from healthy and diabetic (STZ-treated) WT mice (picture magnification: 250×).Representative Western blot of kidney proteins extracted from healthy and diabetic WT and *Timp3*^*−/−*^ mice showing TIMP3 expression levels. Tubulin was used as loading control. ns, non specific bands. Source data is available for this figure in the Supporting Information.Fluorimetric measurement of ADAM17 proteolytic activity in kidneys of WT and *Timp3*^*−/−*^ diabetic, streptozotocin-treated mice compared to untreated control (*n* = 6, Student's *t*-test).Soluble form of TNF-α was measured by ELISA on kidney lysates from normoglycemic and diabetic WT and *Timp3*^*−/−*^mice (*n* = 6, Student's *t*-test).Western blot assessment of membrane-bound and soluble TNF-α in kidneys from healthy and diabetic WT and *Timp3*^*−/−*^ mice. Actin was used as loading control.PAS staining of kidney sections from diabetic and normoglycemic WT and *Timp3*^*−/−*^ mice. Arrow indicates arteriolar hyalinosis at the vascular pole, stars indicate tubular dilation and atrophy and open arrowheads indicate interstitial expansion with fibrosis. Picture magnification is shown on the left. Quantification of mean glomerular area and fractional mesangial area is shown. Statistical significance was evaluated by one-way Anova.

To test the role of TIMP3 and ADAM17 in DKD we treated C57Bl6 WT mice with streptozocin (STZ) to induce hyperglycaemia. We identified a decrease in *Timp3* mRNA expression in diabetic mice (Fig 1A), while the other members of this family (*Timp1*, *2* and *4*) remained unaffected, along with *Adams 10*, *15* and *17* (Fig 1B). This reduction of *Timp3* expression was confirmed at protein level by immunohistochemistry (Fig 1C and Supporting Information Fig S1) and Western blot analysis on WT and *Timp3*^*−/−*^ kidneys from control and diabetic mice (Fig 1D). To assess the significance of TIMP3 reduction in this context we measured ADAM17 activity and TNF-α shedding on kidney homogenates from WT and *Timp3*^*−/−*^ healthy and diabetic mice, as well as circulating TNF-α levels in serum from the same animals. Diabetes induced an increase in ADAM17 activity in the diabetic state, and ADAM17 activity was significantly higher in *Timp3*^*−/−*^ mice compared to WT diabetic littermates (Fig 1E); we also found that ADAM17 activity was increased at the same extent in both right and left kidneys of the two strains (Supporting Information Fig S2A). These analyses confirmed that in diabetic conditions a reduction of TIMP3 occurs, which leads to an increase in ADAM17 metalloprotease activity and TNF-α shedding (Fig 1E–G).

### Analysis of kidneys from WT and *Timp3*^*−/−*^ diabetic mice

**Figure 2:**
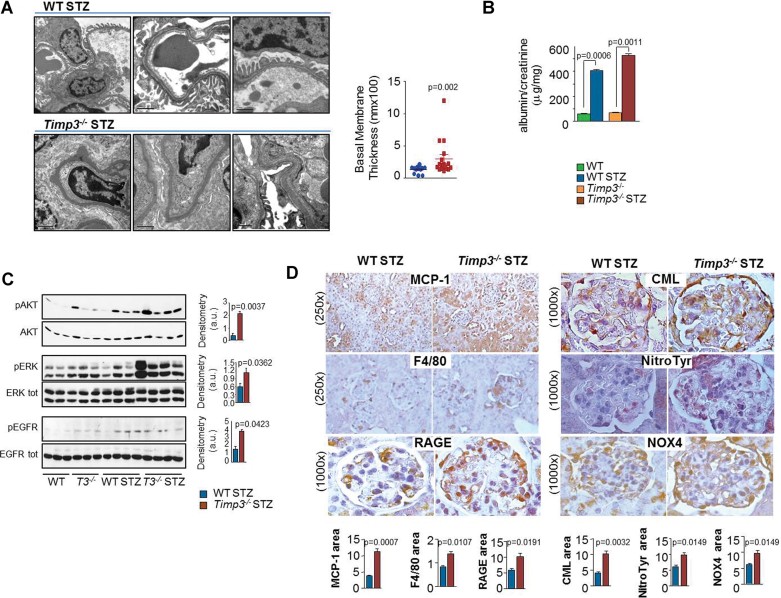
Analysis of kidneys from WT and *Timp3^−/−^* diabetic mice Electron microscopy of kidney sections from WT and *Timp3*^*−/−*^ diabetic mice. Quantification of basal membrane thickness is shown on right panel (Student's *t*-test).Microalbuminuria was determined in WT and *Timp3*^*−/−*^ control and diabetic mice as ratio between urinary albumin and creatinine concentration measured by ELISA (*n* = 3, Student's *t*-test).Representative western blot analysis of lysates from kidneys of healthy and diabetic WT and *Timp3*^*−/−*^ mice. Levels of phosphorylation of Akt (Ser473), ERK (Thr202/Tyr204) and EGFR (Tyr1068) were quantified for STZ-treated animals and expressed as fold increase in the ratio of phospho-protein to total protein (*n* = 6, Student's *t*-test). Source data is available for this figure in the Supporting Information.Immunostaining of kidney sections from WT and *Timp3*^*−/−*^ diabetic mice. Quantification of each staining is shown on the bottom, picture magnification on the left (*n* = 6, Student's *t*-test). CML, *N*-carboxymethyl-lysine; NitroTyr, nitrotyrosine.

Next, we treated WT and *Timp3*^*−/−*^ mice with STZ for 12 weeks to generate overt diabetes (Supporting Information Table S1 and Fig S2B). Kidneys were then removed and analysed by PAS staining (Fig 1H and Supporting Information Fig S3). STZ-treated diabetic *Timp3*^*−/−*^ mice showed significantly increased mean glomerular area (mGA; Fig 1H and Supporting Information Fig S2C), fractional and mean mesangial areas (fMA and mMA; Fig 1H and Supporting Information Fig S2D and E), glomerusclerosis index (GSI), tubulointerstial damage index (TI) compared to both untreated *Timp3*^*−/−*^ littermates and WT control and diabetic mice (Supporting Information Fig S2F and G). The same indexes of kidney damage were evaluated in mice resistant to STZ treatment (STZ low glucose, STZ LG); they were not significantly different in STZ LG and vehicle treated mice (control group); since STZ LG mice showed a random blood glucose levels below 200 mg/dl, they were not further included in the study (Niranjan et al, 2008); from this point on STZ refers only to mice with frank diabetes (random fed glucose >300 mg/dl at the weekly control; Supporting Information Fig S2B). STZ-*Timp3*^*−/−*^ mice also exhibited increased signs of fibrosis and a thicker glomerular basement membrane due to increased amounts of type IV collagen and fibronectin deposition (Supporting Information Fig S2H and S4A–C). Electron microscopy analysis of STZ-*Timp3*^*−/−*^ kidney showed increased basal membrane thickness (Fig 2A) associated with increased albuminuria (Fig 2B). Analysis of signalling pathways activated in diabetic kidneys revealed significant increases in Akt, ERK1/2 and EGFR phosphorylation in *Timp3*^*−/−*^ mice compared to WT littermates (Fig 2C). Moreover, STZ *Timp3*^*−/−*^ kidney had increased macrophage infiltration, measured by MCP-1 expression and F4/80 staining as well as higher levels of RAGE (Fig 2D and Supporting Information Fig S5–S7) compared to controls, which implied a higher grade of inflammation. Oxidative stress markers staining revealed increased expression of *N*-carboxymethyl-lysine (CML), a major product of oxidative modification of glycated proteins, nitro-tyrosine and NOX4, in *Timp3*^*−/−*^ mice (Fig 2D and Supporting Information Figs S8–S10) compared to WT diabetic controls.

### Microarray profiling of kidneys from WT and *Timp3*^*−/−*^ diabetic mice

**Figure 3:**
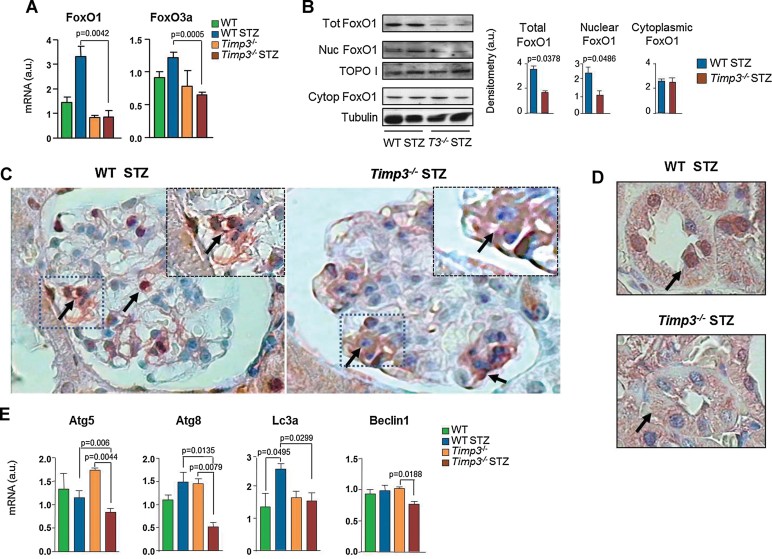
FoxO1 regulation in healthy and diabetic *Timp3^−/−^* kidneys Real-time PCR on kidney mRNA from healthy and diabetic WT and *Timp3*^*−/−*^ mice showing FoxO1 and FoxO3A levels of expression (*n* = 6, Student's *t*-test).Western blot analysis of cytoplasmic, nuclear and total lysates from kidneys of WT and *Timp3*^*−/−*^ diabetic mice. Topoisomerase I (TOPO I) and tubulin were used to normalize levels of nuclear and cytoplasmic proteins, respectively. Densitometric analysis of results are shown on the right (*n* = 6, Student's *t*-test). Source data is available for this figure in the Supporting Information.Foxo1 immunostaining on kidney sections from WT and *Timp3*^*−/−*^ diabetic mice. Arrows indicate nuclear staining in STZ-WT mice (left panel) or cytoplasmic staining in STZ-*Timp3*^*−/−*^ mice (right panel). Magnification: 400×.Higher magnification of panel C (1000×) showing FoxO1 staining in renal tubules (inserts are from Supporting Information Figure S13).Real-time PCR analysis of autophagy related FoxO1 target genes in WT and *Timp3*^*−/−*^ diabetic and normoglycemic kidney (*n* = 6, Student's *t*-test).

To seek the mechanisms by which TIMP3 deficiency may worsen diabetic nephropathy we profiled STZ-WT and STZ-*Timp3*^*−/−*^ kidneys by microarray analysis (Supporting Information Fig S11A). Analysis of the gene ontology showed major differences in clusters of genes involved in inflammation (*Cxcl9*, *Ccr5*, *Mcp-1*, *Mcp-5*, *Aif-1*, *Cd36*, *Mgl-1*, *Mgl-2*, *IkBα*, *IkBβ*, *SOCS-2*), cell proliferation and fibrosis (*Pdgf-d*, *Tgfb3*, *Fgf*, *Ghr*), lipid metabolism (*Fabp5*, *Fasn*, *Ldlr*, *Acaca*, *Acsm3*) and metabolite transport (*Slc13a1*, *Slc7a13*, *Slc7a6*, *Slco4c1*, *Slc12a3*, *Glut8*) in STZ-*Timp3*^*−/−*^ mice compared to STZ-WT controls (Supporting Information Table S2). We chose genes belonging to the inflammatory cluster to validate the microarray results by quantitative PCR on a larger group of mice (*n* = 6 per group; Supporting Information Fig S11B) in which separate groups of non-diabetic controls were also included. Interestingly, STZ-*Timp3*^*−/−*^ mice also showed a significant reduction in the expression of transcription factors connected to the control of oxidative stress such as *Foxo1* and *Foxo3a* (0.6- and 0.5-fold change, respectively; Fig 3A), along with several FoxOs targets (Supporting Information Fig S11C). No significant differences in the expression of these genes were observed when normoglycemic WT and *Timp3*^*−/−*^ mice were compared (Fig 3A).

### FoxO1 regulation in diabetic *Timp3*^*−/−*^ kidneys

Giving the importance of FoxO1 in regulating cell survival and oxidative stress (Nemoto & Finkel, 2002), and renal neoplastic cell proliferation (Gan et al, 2010), we next focused on FoxO1 regulation in STZ-*Timp3*^*−/−*^ kidney. IHC staining of renal sections from STZ-WT and STZ-*Timp3*^*−/−*^ mice confirmed a decrease in FoxO1 expression in the KO strain compared to the WT, while there were no significant differences on FoxO1 expression in healthy WT and *Timp3*^*−/−*^ mice (Supporting Information Fig S12). Importantly, analysis of subcellular compartments revealed that the pool of nuclear Foxo1 (*i.e.* the transcriptionally competent fraction) was mostly affected in STZ-*Timp3*^*−/−*^ mice, as the amount of cytoplasmic FoxO1 remained unchanged in both genotypes (Fig 3B and Supporting Information Fig S13). Immunohistochemical analysis supported the prevalent effect of STZ-*Timp3*^*−/−*^ on nuclear FoxO1, both in the glomerular and tubular compartments (Fig 3C, D and Supporting Information Fig S13). Consistently with FoxO1 exclusion from the nucleus, the expression of several FoxO1 target genes (such as *Ccnd2*, *Cdkn1a*, *Igfbp1*, *Gadd45* and *Ucp2*) that were down-regulated in the microarray analysis, showed also a significant reduction when STZ-*Timp3*^*−/−*^ kidneys were compared to STZ-WT control by quantitative PCR (Supporting Information Fig S14A). Interestingly, FoxO1 targets not found in the microarray analysis (*Bim*, *FasL*) maintained a similar unchanged pattern of expression in PCR validation (Supporting Information Fig S14B). One peculiar set of FoxO1 targets that was consistently downregulated in the microarray and confirmed by PCR validation included *Atg5*, *Atg8*, *LC3a* and *Beclin1* (Fig 3E); all these genes are primarily involved in regulation of autophagy, a lysosomal protein degradation pathway that plays a crucial role in removing protein aggregates as well as damaged or excess organelles to maintain intracellular homeostasis and cell integrity. However, other pathways known to be involved in the pathogenesis of diabetic nephropathy through intracellular stress induced-autophagy (such as AMPK and mTOR activity, hypoxia and ER stress; Fogo, 2011; Godel et al, 2011; He et al, 2010; Inoki et al, 2011; Kume et al, 2010, 2012; Nath, 2010) were not affected in our models (Supporting Information Fig S15A–C).

### TIMP3^knockdown^ MES 13 mesangial cells recapitulate *in vitro* FoxO1 regulation *in vivo*

**Figure 4:**
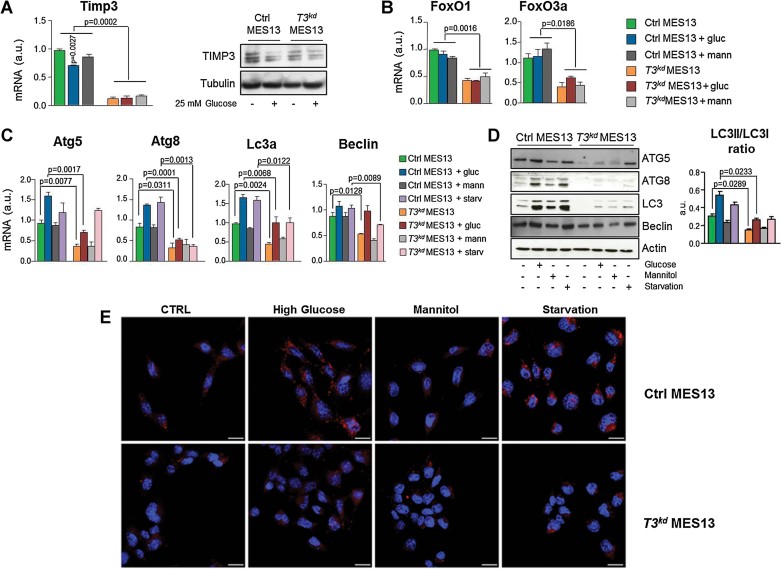
FoxO1 regulation in *T3^kd^* MES13 cell line Analysis of Timp3 mRNA (left panel) and protein (right panel) expression in *T3*^*kd*^ or control MES13 cells grown in basal medium (low glucose) or treated with high glucose (25 mM) for 48 h before harvesting. Mannitol treatment was included in the real time PCR experiment as osmolarity control (*n* = 3, Student's *t*-test). Source data is available for this figure in the Supporting Information.Real-time PCR on *T3*^*kd*^ and control MES13 cells treated as in (**A**), showing FoxO1 and FoxO3A mRNA modulation (*n* = 3, Student's *t*-test).Expression of autophagic FoxO1 target genes in *T3*^*kd*^ and control MES13 cells treated with high glucose or mannitol (25 mM), or serum-starved for 24 h before harvesting (*n* = 3, Student's *t*-test).Western blot analysis of autophagic FoxO1 target genes on lysates from *T3*^*kd*^ and control MES13 cells treated as in (**C**). Ratio between LC3II and LC3I form is shown on the left (*n* = 3, Student's *t*-test).Immunofluorescence (IF) for LC3A/B (red) in *T3*^*kd*^ and control MES13 cells treated as in (**A**). Cells were counterstained with DAPI to detect nuclei (blue). Magnification views: 60×. Scale bars: 20 µm.

**Figure 5:**
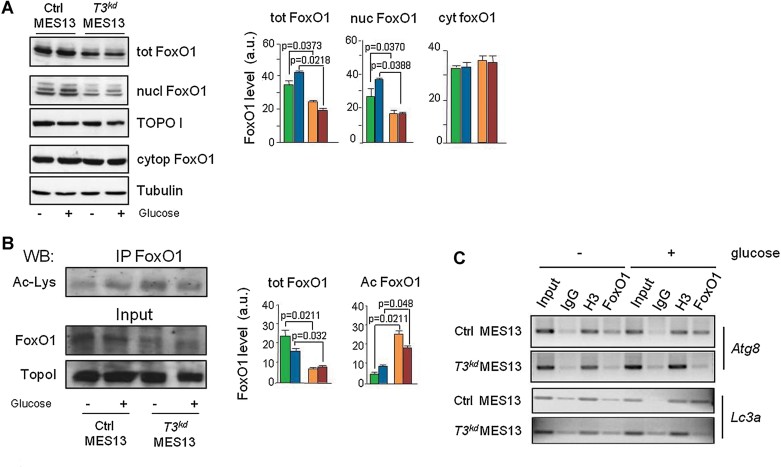
FoxO1 subcellular distribution in *T3^kd^* MES13 cell line Representative Western blot analysis of total, nuclear and cytoplasmic lysates from *T3*^*kd*^ and MES13 control cells grown in low or high glucose. Topoisomerase I and tubulin were used to normalize levels of nuclear and cytoplasmic proteins, respectively. Densitometric analysis of results is shown on the right (*n* = 3, Student's *t*-test). Source data is available for this figure in the Supporting Information.Nuclear FoxO1 protein was immunoprecipitated from *T3*^*kd*^ and control MES13 cells grown in low or high glucose and then subjected to Western blot with an anti-acetyl-lysine antibody. FoxO1 and Topoisomerase I were used as loading control. Densitometric analysis of results is shown on the right (*n* = 3, Student's *t*-test).Chromatin immunoprecipitation (ChIP) on *T3*^*kd*^ or control MES13 cells. Primers used to amplify the FoxO1 binding site on *LC3a* and *Atg8* promoters are described in the Materials and Methods section. IgG, negative control; H3 antibody, positive control.

To investigate the molecular role of FoxO1 in the regulation of autophagy in diabetic nephropathy, we generated *Timp3*^*knockdown*^ cells by stably transfecting SV40 MES13 cells with three shRNA plasmids that target different sequences in the *Timp3* mRNA (*T3*^*kd*^ MES13). These cells exhibited a significant reduction in *Timp3* mRNA and protein (Fig 4A), and similarly to STZ-*Timp3*^*−/−*^ kidney, higher inflammatory response (up-regulation of *ccl2* and down-regulation of *IkBalpha* and *IkBbeta*) and activation of signal transduction pathways (increased EGFR, Akt and ERK phosphorylation; Supporting Information Fig S17A and B), confirming the validity of this model to further explore the role of Timp3/FoxO1 axis in autophagy. As expected *T3*^*kd*^ MES13 cells showed a decreased expression of Foxo1, FoxO3A (Fig 4B) and expression of the autophagy genes *Atg5*, *Atg8*, *Lc3a* and *Beclin* was also reduced, both at the mRNA (Fig 4C) and protein level (Fig 4D). LC3II/I ratio, which is enhanced in the course of autophagy activation, confirmed that the autophagic process was impaired in *T3*^*kd*^ MES13 compared to control MES13. A similar pattern was confirmed by immunofluorescence study of LC3 redistribution inside cells, a marker of autophagosome assembly, that was impaired in *T3*^*kd*^ MES13 cells compared to control MES13 both in high glucose conditions and in a positive control for autophagy activation such as serum starvation (Fig 4E and Supporting Information Fig S16). Moreover, in *T3*^*kd*^ MES13 cells FoxO1 compartmentalization was also altered, and a decrease in the nuclear Foxo1 pool seemed to entirely account for the overall reduction of protein level observed in total lysate of *T3*^*kd*^ MES13 cells, as was observed in STZ-*Timp3*^*−/−*^ mice (Fig 5A). Interestingly, nuclear FoxO1 exhibited a higher degree of acetylation in *T3*^*kd*^ MES13 cells compared to the controls (Fig 5B), and we rationalized this to underlie its nuclear exclusion (Frescas et al, 2005) along with a decline of its transcriptional activity; recent studies have demonstrated that FoxO1 acetylation results in its loss of function (Banks et al, 2011). To verify this hypothesis, we performed chromatin immunoprecipitation on *T3*^*kd*^ and control MES13 cells, either untreated or treated with high glucose. We found that binding of FoxO1 to *Atg8* and *Lc3a* promoters was significantly reduced in the absence of TIMP3 (Fig 5C), explaining the reduction in their mRNA expression observed both in *Timp3*^*−/−*^ mice and cells. This reduction was reverted by infecting *T3*^*kd*^ and control MES13 cells with an adenovirus over-expressing constitutively nuclear FoxO1 (FoxO1-ADA), confirming a direct role of this transcription factor in regulating autophagy-related gene expression in Timp3 knockdown cells (Supporting Information Fig S18).

### FoxO1 regulation in primary mesangial cells

**Figure 6:**
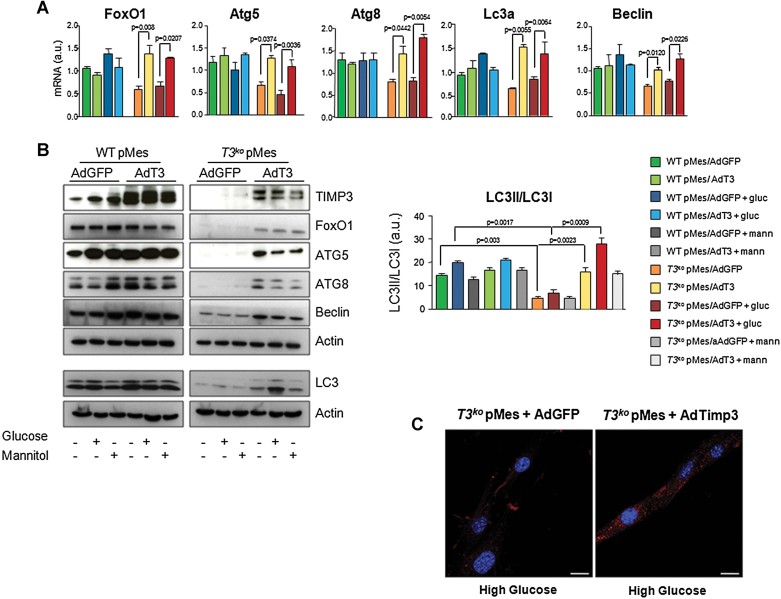
Re-expression of TIMP3 in *T3^ko^* primary mesangial cells rescues FoxO1 effect on autophagy genes Rescue of expression of FoxO1 and its targets in *T3*^*ko*^ primary cells (pMes) following reintroduction of TIMP3. RNA from *T3*^*ko*^ or WT pMes cells infected with GFP or TIMP3 adenovirus and grown either in low or high glucose was used for real-time PCR analysis of FoxO1, Atg5, Atg8, LC3a and Beclin expression (*n* = 3, Student's *t*-test).Western blot analysis of *T3*^*ko*^ and WT pMes cells infected and treated as in (**A**). Samples were run on a 4–12% gradient gel (TIMP3, FoxO1, ATG5, ATG8, Beclin and Actin) or on a 15% gel (LC3 and actin). Ratio between LC3II and LC3I form is shown on the left (*n* = 3, Student's *t*-test). Source data is available for this figure in the Supporting Information.Immunofluorescence analysis of LC3A/B (red) in *T3*^*ko*^ pMes cells infected with AdGFP (left) and AdTimp3 (right) in HG condition. Cells were counterstained with DAPI to detect nuclei (blue). Magnification views: 60×. Scale bars: 20 µm.

To demonstrate that FoxO1 down-regulation in *Timp3*^*−/−*^ mice and cells was due to the absence of TIMP3, we used a more physiological cell model and purified primary mesangial cells (pMes) from WT and *Timp3*^*−/−*^ mice. Primary mesangial cells derived from *Timp3*^*−/−*^ mice (*T3*^*ko*^ pMes) showed a higher degree of ADAM17 activation, and a consequent increase of TNF-α release in the growing medium compared to the WT pMes cells. Both features were promptly reversed by infection of these cells with a Timp3 adenovirus (Supporting Information Fig S19A and B). As shown in Fig 6A and B, TIMP3 over-expression was also sufficient to rescue FoxO1 mRNA and protein expression, either in low or high glucose conditions, as well as that of ATG5, ATG8, LC3 and Beclin, demonstrating a direct link between *Timp3* deficiency and FoxO1 down-regulation. Since the mRNA and protein studies suggested that reintroduction of TIMP3 in *T3*^*ko*^ pMes might restore the autophagic process, we analysed LC3 redistribution in *T3*^*ko*^ pMes infected with a Timp3 adenovirus (*T3*^*ko*^ pMes + AdTimp3) or an adenovirus encoding for GFP as control. These experiments confirmed that TIMP3 was able to enhance LC3 redistribution under both high glucose and serum deprivation condition, suggesting an effect on the autophagy machinery (Fig 6C and Supporting Information Fig S20).

### STAT1 regulates FoxO1 expression in *Timp3*^*−/−*^ diabetic mice and cells

**Figure 7:**
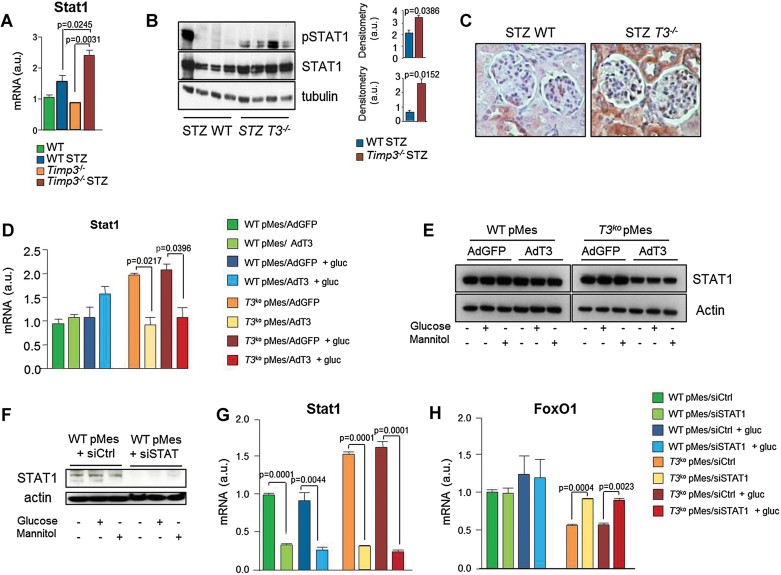
FoxO1 and STAT1 interplay Real-time PCR of STAT1 expression in WT and *Timp3*^*−/−*^ diabetic and normoglycemic kidney (*n* = 6, Student's *t*-test).Representative western blot analysis of STAT1 expression in kidneys from healthy and diabetic WT and *Timp3*^*−/−*^ mice. Quantification for STZ-treated animals is shown on the right (*n* = 3, Student's *t*-test). Source data is available for this figure in the Supporting Information.Immunohistochemical staining of diabetic WT and *Timp3*^*−/−*^ kidney sections showing STAT1 expression. Magnification: 250×.Real-time PCR of STAT1 expression in *T3*^*ko*^ or WT pMes cells infected with GFP or TIMP3 encoding adenovirus (*n* = 3, Student's *t*-test).Western blot analysis of STAT1 expression in *T3*^*ko*^ or WT pMes cells infected with GFP or TIMP3 encoding adenovirus (representative analysis of three independent experiments with the same results).Western blot analysis on control cells transfected with a pool of control or STAT1 siRNA, confirming inhibition of STAT1 expression.Real-time PCR on *T3*^*ko*^ or control pMes cells transfected with a pool of siRNA directed against STAT1, showing reduction of STAT1 expression (*n* = 3, Student's *t*-test).Real-time PCR on *T3*^*ko*^ or WT pMes cells transfected with a pool of siRNA directed against STAT1, showing reciprocal regulation of STAT1 and FoxO1 expression (*n* = 3, Student's *t*-test).

Among the genes differentially regulated in the kidney of diabetic *Timp3*^*−/−*^ mice, *Stat1* was noted to be over-expressed between three- and sevenfold in the KO strain compared to the WT. As STAT family members have been loosely implicated in the regulation of FoxO1 promoter, and STAT1 in particular has a negative effect on FoxO1 transcription (Luo et al, 2011; Ono et al, 2007), we next analysed STAT1 expression in mice. A significant increase of STAT1 mRNA and protein occurred in *Timp3*^*−/−*^ diabetic kidneys compared to the WT (Fig 7A–C and Supporting Information Fig S21). Real-time PCR analysis showed that primary mesangial cells derived from *Timp3*^*−/−*^ kidneys had higher STAT1 mRNA and protein expression than controls, regardless of the cell exposure to high glucose concentrations (Fig 7D). This regulation seemed to be directly linked to deficiency of *Timp3*, as infection of these cells with TIMP3 adenovirus promptly decreased STAT1 mRNA and protein expression to control levels (Fig 7D and E). To further verify the role of STAT1 in FoxO1 regulation, we transfected our *T3*^*ko*^ and control pMes cells with a pool of siRNAs directed against *Stat1* and found that abolishing STAT1 expression in *T3*^*ko*^ pMes cells resulted in a rescue of FoxO1 levels, evoking a possible role of STAT1 as a mediator between *Timp3* deficiency and FoxO1 regulation (Fig 7F–H).

### TIMP3/FoxO1 interplay in human diabetic kidney disease

**Figure 8:**
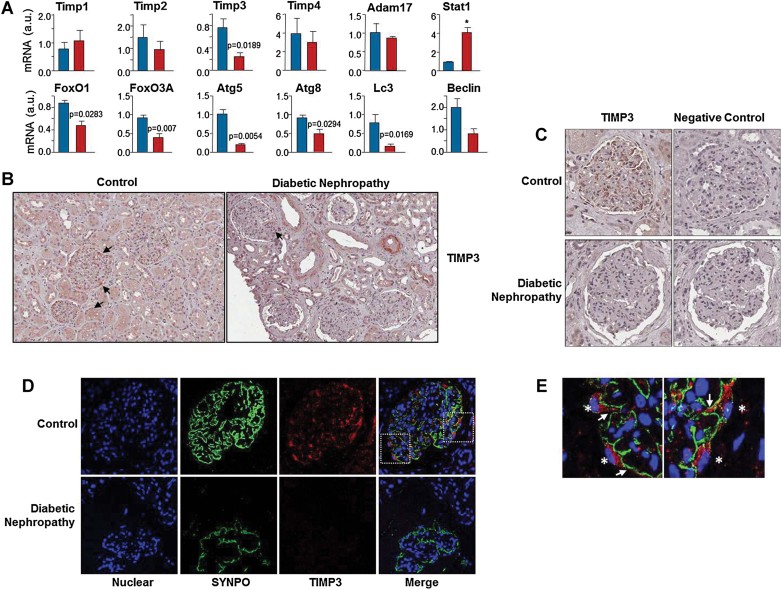
TIMP3 and FoxO1 regulation in diabetic patients Real-time PCR on RNA extracted from four controls and five patients affected by Diabetic Nephropathy (Student's *t*-test).TIMP3 and control staining of kidney sections from healthy and diabetic subjects. Arrows indicate some TIMP3 positive cells. 40× scanning magnification, 10× zoom.Higher magnification of panel (**B**) (20× zoom).Co-immunofluorescence of control and diabetic human kidney sections showing nuclei (blue) synaptopodin (green) and TIMP3 (red). Merged images are shown on the right panels. Magnification: 63×.Higher magnification of panel (**D**) (20× zoom). Synaptopodin immunolabelling (green) highlights podocyte foot processes and is characterized by thin linear staining along the surface of the glomerular basement membranes (GBM) of capillary loops. Timp3 immunoreactivity (red) is mainly observed in the podocyte cell body (*) and primary processes (white arrows).

Diabetic nephropathy is a serious complication of diabetes mellitus in humans, and has become a major health problem worldwide. We explored whether the interplay between TIMP3, FoxO1 and STAT1 was indeed present in humans, and performed real time PCR analysis of Adam17 and the four Timp genes in kidney biopsies from five diabetic patients and four healthy controls (Supporting Information Table S3). Timp3 was significantly reduced in diabetic patients (Fig 8A). Consistent with the results obtained in mice, the patients also showed a diminished expression of *FoxO1* and *FoxO3A*, as well as that of *Atg5*, *Atg8*, *Lc3a* and *Beclin* (Fig 8A) by quantitative PCR. Moreover, *Stat1* gene expression was significantly increased in diabetic subjects (Fig 8A). IHC on kidney sections from these patients confirmed a reduction of TIMP3 and FOXO1 and increase of STAT1 protein in all compartments, particularly in glomeruli, while TIMP3 staining was intact in normal renal tissue (Fig 8B and C, Supporting Information Fig S22 and S23). Immunofluorescence analysis confirmed that in the glomerular area TIMP3 is localized in the extracellular compartment and may play a role to block the activity of ADAM17 and other proteases on multiple cell types (Fig 8D). Enlargement of immunofluorescence images also showed positive TIMP3 immunoreactivity in podocyte cell body and primary processes suggesting that podocyte cells are a site of production of TIMP3 (Fig 8E).

## DISCUSSION

Our study demonstrates a previously unknown mechanism in which the absence of TIMP3, a metalloprotease inhibitor, exacerbates renal damage in response to a chronic hyperglycaemic stress caused by diabetes. MMPs produced by the mesangial cells account for up to 70% of extracellular matrix degradation and turnover in the kidney. Abnormal extracellular matrix deposition is the hallmark of diabetic nephropathy, and a number of studies have reported a link between aberrant MMP expression/activation and the progression of diabetic nephropathy (Catania et al, 2007). TIMP3 is the most highly expressed TIMP in the kidney and has a broad protease inhibition profile; loss of this protein is associated with age-dependent renal fibrosis and tubulointerstitial injury in mice (Kassiri et al, 2009; Kawamoto et al, 2006). Moreover, TIMP3 is the only known physiological inhibitor of ADAM17, a metalloprotease responsible for shedding of several ligands, in particular HB-EGF and TGFα, which are involved in the pathogenesis of chronic kidney disease and glomerulonephritis (Bollee et al, 2011; Lautrette et al, 2005). ADAM17 also participates in the generation of transcriptionally active form of Notch, which is important in glomerular development and regulation of podocytes dysfunction (Niranjan et al, 2008).

Here we report that TIMP3 expression was reduced in the kidney of STZ treated-mice, a well-established model of hyperglycemia and glucotoxicity. *Timp3*^*−/−*^ diabetic kidneys showed a higher degree of inflammation and some evidence of podocyte dysfunction compared to WT diabetic control, indicating that loss of TIMP3 is detrimental to the progression of DKD. These effects arise due to unrestrained activation of ADAM17 which results in a systemic increase in TNF-α signalling and additionally in EGFR activation (Black, 2004). Through gene expression profiling of *Timp3*^*−/−*^ diabetic kidneys, our study confirms that many critical mediators of inflammation and proliferation are found up-regulated, although we did not observe a perturbation of the VEGFR pathway that has been reported to bind TIMP3 (Qi et al, 2003). Noteworthy were the down-regulation of FoxOs transcription factors and some of their target genes, especially those involved in the control of autophagy. FoxO transcription factors are implicated in regulating diverse cellular functions, including differentiation, proliferation, metabolism and survival (Hedrick, 2009; van der Vos & Coffer, 2011). In the kidney, changes in their expression levels may represent a connection between altered metabolic and inflammatory cues which characterize diabetic nephropathy. It is conceivable that TIMP3 deficiency, through an increase in ADAM17 sheddase activity, may concur to dampen FoxO1 activity in two ways. First, an increase in circulating TNF-α levels may be responsible for the observed transcriptional induction of STAT1, which acts as a repressor for FoxO1 promoter and attenuates its expression. While the majority of FoxO1-related research focuses on the FoxO1 function as a transcriptional activator/repressor, the underlying mechanisms that govern FoxO1 gene transcription *per se* are largely unknown. It has been shown that members of the STAT family of transcription factors can bind FoxO1 promoter (Luo et al, 2011; Ono et al, 2007) and STAT1 exerts a negative role on FoxO1 promoter activity in RINm5F cells (Luo et al, 2011). Actually, *Stat1* was over-expressed in *Timp3*^*−/−*^ diabetic kidney compared to the WT; moreover, abolishing *Stat1* expression by RNA interference caused a complete rescue of FoxO1 expression, suggesting a possible role of STAT1 in linking *Timp3* deficiency to FoxO1 regulation.

Second, the activation of EGFR and AKT pathways that we observed in diabetic *Timp3*^*−/−*^ kidney and *T3*^*kd*^ MES13 cells may explain the reduction of the nuclear pool of FoxO1 protein and the consequent attenuation of its transcriptional activity, as indicated by microarray and ChIP analyses. In *T3*^*kd*^ MES13 cells, the amount of FoxO1 protein in the nucleus was not only reduced but also hyper-acetylated compared to control cells. It has been shown that this post-transcriptional modification acts as an ‘off’ signal for FoxO activity (Banks et al, 2011), and it additionally promotes a rapid export of FoxO1 from the nucleus to the cytoplasm, which correlates with a decrease in transcriptional activity of the protein. Thus, in our systems we envision a ‘two-hit hypothesis’ by which TIMP3 deficiency impacts FoxO1 function: a transcriptional down-regulation of the gene (through STAT1 over-expression) and a nuclear exclusion caused by EGFR/AKT phosphorylation and/or hyper-acetylation. This unexplored effect of TIMP3 on FoxO1 function in the kidney is dependent on a hyperglycaemic environment which contributes to reduce TIMP3 expression possibly through epigenetic regulation of its promoter (Cardellini et al, 2009; Federici et al, 2002). Our *in vitro* data suggest that TIMP3 impinges on FoxO1 expression and transcriptional activity through complex mechanisms, since knockdown or over-expression of TIMP3 in cells is able to reproduce or rescue this effect on FoxO1, respectively.

Recently, some of the FoxO1 and FoxO3A target genes were shown to be involved in autophagic protection of skeletal myocytes, cardiomyocytes and neurons from stress conditions (Hariharan et al, 2010; Masiero et al, 2009; Medema & Jaattela, 2010; Sengupta et al, 2009; van der Vos et al, 2011; Xu et al, 2011; Zhao et al, 2007). Autophagy can also be induced by intracellular stresses that are involved in the pathogenesis of diabetic nephropathy including hypoxia or ER stress (Kume et al, 2012; Mizushima & Komatsu, 2011), and plays an important role as a survival factor especially in post-mitotic cells such as podocytes (Fogo, 2011; Godel et al, 2011; Hartleben et al, 2010; He et al, 2010; Inoki et al, 2011; Kume et al, 2010; Nath, 2010). Activated FoxOs stimulate autophagy mainly through a transcription-dependent mechanism and increase the expression of several autophagy-related genes, such as, *Atg5*, *Atg8*, *Lc3* and *Atg12*, as part of a general mechanism of oxidative stress resistance (Kume et al, 2012). However, Zhao and colleagues recently described a different mechanism in which the cytoplasmic pool of acetylated FoxO1 molecules appeared to be essential for induction of autophagy (Zhao et al, 2010), and FoxO1-mediated induction of autophagy was independent of FoxO1 transcriptional activity. Thus we hypothesize that in a diabetic, metabolically stressed context the effect of TIMP3 deficiency on the FoxO-regulated autophagic pathway may contribute, through the ‘two-hit’ model, to attenuate the protective function of the autophagic process and hence worsen diabetic nephropathy.

Previous studies have focused on the involvement of TIMP3 in kidney pathology (Kassiri et al, 2009; Kawamoto et al, 2006): loss of TIMP3 associated with renal fibrosis and tubular interstitial injury in a mouse model of unilateral urethral obstruction (UUO; Kassiri et al, 2009), even though in human kidney biopsies TIMP3 expression was shown to be increased in patients with diabetic nephropathy secondary to T2DM or with chronic allograft nephropathy compared to healthy controls, possibly as a compensatory mechanism aimed at minimizing renal damage and disease progression. We also analysed kidney biopsies from diabetic patients and found a significant decreased of TIMP3 expression especially in diabetic glomeruli compared to the controls. Consistently with our findings, in a recent transcriptome analysis of human DKD biopsies, TIMP3 was significantly down-regulated in glomeruli but not in tubuli of diabetic kidneys compared to their healthy controls (Woroniecka et al, 2011), and it is therefore possible that TIMP3 may have different roles in these distinct compartments, and/or may be differentially regulated at subsequent stages of the progression of diabetic nephropathy (Kassiri et al, 2009). Consistently with the results obtained in *Timp3*^*−/−*^ mice we found increased STAT1 expression and reduced FoxO1 expression in the diabetic biopsies. Moreover, since also the expression of autophagic genes was found reduced in our patients with diabetic nephropathy, we might speculate that there is a parallel between the two models.

Our study has clearly some limitations: being TIMP3 an extracellular protein it is conceivable that its reduction affects more than one cell type, including podocytes, and therefore some of the effects that we have observed in this study may be not cell specific but indirect. Furthermore, the effect of increased ADAM17 activity may differ depending on its distribution between the different compartments of the kidney. Finally, the human data are limited given that the source material does not allow performing all the experiments as in experimental models and some differences with the experimental models might be due to concomitant medications used in patients.

Nevertheless, our study demonstrates that loss of TIMP3 is a hallmark of DKD in human and mouse models. Reduction of TIMP3 causes a concomitant STAT1-dependent and compartment-specific loss of FoxO1 activity, which in turn diminishes the expression of protective autophagy genes to fuel glomeruli damage in a mouse model. Thus, TIMP3 plays an important function in maintaining kidney homeostasis and represents a new protective candidate to be explored for controlling diabetic nephropathy.

## MATERIALS AND METHODS

### Reagents

STZ, glucose, proteinase K and other common chemicals were from Sigma–Aldrich (St. Louis, MO). Anti p-Akt (#9271), Akt (#9272), p-ERK (#9101), ERK (#9102), p-EGFR (#2234), p-FOXO1 (#9461), FOXO1 (#2880), acetyl-lysine (#9441), mTOR (#2972), p-mTOR (#2971), p70S6k (#2708), p-p70S6k (#9205) and LC3A/B (#4108) antibodies were from Cell Signaling (Beverly, MA). TIMP3 (#39185), TNF-α (#9739), Fibronectin (#6328), ATG5 (#78073), ATG8 (#86947) and BECLIN (#16998) antibodies were from Abcam (Cambridge, UK). Actin (#1616), tubulin (#53646), EGFR (#03), Topoisomerase I (#5342), STAT1 (#592) and p-STAT1 (#136229) antibodies were from Santa Cruz (Santa Cruz, CA). Small interfering STAT1 (#44124) and control (#37007) RNAs were from Santa Cruz.

### Animal models and induction of diabetes

*Timp3*^*−/−*^ mice were previously described (Federici et al, 2005). The animal methods are described *in extenso* in the online only Supporting Information section.

### TACE activity

TACE activity was determined using the SensoLyte 520 TACE Activity Assay Kit (AnaSpec, San José, CA), accordingly to the manufacturers protocol. Thirty micrograms of tissue proteins were used for the assay. Reaction was started by adding 40 µM of the fluorophoric QXL520/5FAM FRET substrate. Fluorescence of the cleavage product was measured in a fluorescence microplate reader (FLx800, BIO-TEK Instruments, Winooski, VT) at lex 490 nm and lem 520 nm.

### TNF-α ELISA

Measurements of TNF-α concentration in mice sera and cells supernatants were obtained using an ELISA kit (R&D Systems), according to the manufacturer's protocol.

### Measurement of albuminuria

Prior to sacrifice, mice were placed into metabolic cages for a 24 h urine collection. Urine albuminuria was determined using a mouse albumin ELISA kit (Assaypro, St. Charles, MO) and a mouse creatinine ELISA kit (Cusabio, Newark, DE) according to the manufacturers' instructions. Values of urine albumin were normalized against urine creatinine concentration.

### Histological analysis and quantification of renal lesions

All methods are reported in the Supporting Information section.

### EM analysis

Kidney specimens were fixed in 0.1 mol/L phosphate-buffered Karnovsky's fixative. Tissue samples were post-fixed in 1% phosphate-buffered osmium and embedded in epoxy resin (Epon 812; Electron Microscopy Sciences, Hatfield, PA). Ultrathin sections were examined by means of Hitachi H-7100FA electron microscope (Hitachi Software Engineering, Yokohama, Japan).

### Cell culture

SV40 MES 13 mesangial cells were obtained from ATCC and cultured in low glucose DMEM. To induce hyperglycaemia, 20 mM glucose was added to the culture medium (25 mM final concentration) for 24–48 h. For osmolarity control, 20 mM mannitol was used. To generate *Timp3*^*kd*^ MES13 cells, WT MES 13 cells were stably transfected with three different pLKO.1 Timp3 shRNA lentiviral vectors (Open Biosystems, Huntsville, AL; *T3*^*kd*^ MES13 cells) or a scramble control shRNA lentiviral vector (Ctrl MES13 cells). After 1-week of puromycin selection, single clones were isolated and analysed for Timp3 expression. For siRNA experiments, primary mesangial cells were transfected with a pool of STAT1 or control siRNA using the Amaxa nucleofector according to the manufacturer instructions.

### Gene expression analysis by RNA microarray

Total RNA was isolated from three WT and three *Timp3*^*−/−*^ mice using the Qiagen RNeasy kit (Qiagen, Valencia, CA). RNA labelling, hybridization, scanning and data analysis was performed by DNA Vision (Charleroi, Belgium) using the GeneChip® Mouse Genome 430 2.0 Array (Affimetrix, Santa Clara, CA) that contains probe sets for over 39,000 transcripts. Expression data were processed in Log2 scale. Differentially expressed transcripts were selected by the Limma package of R-Bioconductor. Heatmaps and hierarchical clusters were created using MeV 4.4 (MultiExperiment Viewer, TM4 suite; Saeed et al, 2006).

The microarray data discussed in this publication have been deposited in NCBI's Gene Expression Omnibus database and are accessible for referees through GEO Series accession number GSE36336 at the following website: http://www.ncbi.nlm.nih.gov/geo/query/acc.cgi?token=xliplckueyyqyds&acc=GSE36336.

**The paper explained**

PROBLEM:

DKD is a major long term complication of diabetes; its prevalence has been increasing worldwide, generating an urgent need to identify new therapeutic targets to prevent diabetic nephropathy. Extracellular matrix accumulation in the glomerular basement membrane is a major feature of this disease, pointing at a possible involvement of matrix metalloprotease in the development of diabetic kidney disease. Activation of ADAM17 (a member of the ADAM subfamily of matrix metalloproteases) has been involved in the pathogenesis of diabetic nephropathy, but the role of this enzyme and its specific inhibitor TIMP3 in the development of diabetic kidney disease is still unknown. Here we investigated whether a loss of TIMP3 contributes to the onset and progression of DKD in a mouse model of diabetes.

RESULTS:

We found that TIMP3 expression was reduced in the kidney of diabetic mice compared to control littermates, while ADAM17 proteolytic activity was increased. Diabetic *Timp3*^*−/−*^ mice showed increased albuminuria and their kidneys presented a higher degree of inflammation along with morphological and molecular alterations of podocytes and increased basal membrane thickness compared to diabetic WT littermates, indicating that loss of TIMP3 is detrimental to the progression of diabetic kidney disease. Gene expression analysis of diabetic *Timp3*^*−/−*^ kidneys showed a significant reduction of *Foxo1* expression, along with FoxO1 target genes involved in autophagy, and an increase of STAT1, a repressor of FoxO1 transcription. Studies on kidney biopsies from patients with diabetic nephropathy confirmed a significant reduction in TIMP3, FoxO1 and FoxO1 target genes involved in autophagy compared to controls, while STAT1 expression was strongly increased.

IMPACT:

Our study suggests that loss of TIMP3 is a hallmark of diabetic kidney disease in human and mouse models. Reduction of TIMP3 causes a concomitant STAT1-dependent loss of FoxO1 activity, which in turn increases the expression of deleterious oxidative genes and diminishes that of protective autophagy genes to fuel glomeruli damage. Therefore, TIMP3 reduction primes the diabetic kidney with reduced ability to use autophagy proteins if needed as a consequence of other processes. Thus, TIMP3 plays an important function in maintaining kidney homeostasis and represents a new possible therapeutic target for controlling diabetic nephropathy.

### Gene expression analysis by qRT-PCR

Total RNA was isolated from different samples using Trizol reagent (Invitrogen Corp, Carlsbad, CA). Two micrograms of total RNA were reverse-transcribed into complementary DNA (cDNA) using the High Capacity cDNA Archive kit (Applied Biosystems, Foster City, CA). Quantitative real-time polymerase chain reaction was performed using an ABI PRISM 7700 System and TaqMan reagents (Applied Biosystems). Each reaction was performed in triplicate using standard reaction conditions and results were normalized by β-actin and 18S ribosomal protein expression in mouse and human samples, respectively. Sequences of the Applied Biosystems primers used are available upon request.

### Preparation of nuclear and cytoplasmic extracts

Kidneys, *T3*^*kd*^ MES13 and control MES 13 cells were resuspended in hypotonic buffer and incubated for 15 min on ice. After centrifugation, supernatants were removed and kept as cytoplasmic fractions. Nuclear pellets were briefly washed in hypotonic buffer, resuspended in hypertonic buffer and incubated on ice for 20 min, vortexing every 5 min. Nuclear extracts were obtained after centrifugation. Both nuclear and cytoplasmic fractions were quantified spectrofotometrically using the Bradford reagent (BioRad, Hercules, CA).

### Western blot and immunoprecipitation

Kidneys and cell lines were lysed in RIPA buffer, total extracts were quantified using the Bradford reagent (BioRad) and then analysed by SDS–PAGE. Nuclear cell extracts were also immunoprecipitated using a FOXO1 antibody from Santa Cruz and then probed with anti-acetyl lysine antibody.

### Chromatin immunoprecipitation

ChIP assays were performed as follow: *T3*^*kd*^ MES 13 and control cells underwent cross-linking with 1% formaldehyde at room temperature for 10 min, followed by quenching with 0.125 M glycine. Cells were then rinsed with cold PBS, detached with tripsin and centrifuged. Cells were lysed in cell lysis buffer (10 mM HEPES pH 8.0, 10 mM NaCl, 0.2% NP40 and protease inhibitors). Nuclei were collected by centrifugation, resuspended in nuclei lysis buffer (50 mM Tris–Cl, pH 8.1, 10 mM EDTA, 1% SDS and protease inhibitors) and incubated on ice for 10 min. Samples were sonicated to an average DNA fragment length of 500 bp and then centrifuged. The chromatin solution was pre-cleared by adding protein A beads (GE Healthcare, Little Chalfont, UK). Immunoprecipitation of chromatin was carried out overnight at 4°C, using 2 µg of control (normal rabbit IgG) or specific (anti-FOXO1 or anti-histone H3) antibodies, and collected by incubation with protein A beads for 2 h. Immunoprecipitates were washed several times with wash buffers at different ionic strengths and finally with TE. Antibody/protein/DNA complexes were eluted in 1% SDS elution buffer, treated with proteinase K (Sigma–Aldrich), and incubated at 65°C overnight to reverse crosslinking. DNA was purified from samples using QIAquick PCR Purification Kit (Qiagen). PCR was performed using 5 µl of immunoprecipitated DNA. Primers sequences are: *Atg8* forward 5′-CCATTCTCCAGCTCCCAATA-3′; *Atg8* reverse 5′-AAGGGCAGTTCTTCAGTCCA-3′; *Lc3a* forward 5′-CATGCCTTGGGACACCAGAT-3′; *Lc3a* reverse 5′-ACCTTCTTCAAGTGCTGTTTGT-3′.

### Immunofluorescence

*T3*^*kd*^ MES13 and control cells were treated with 25 mM glucose or mannitol, or serum-starved for 24 h, then washed in PBS and fixed for 15 min with 4% paraformaldehyde, permeabilized with methanol for 10 min at −20°C and blocked in PBS containing 5% normal goat serum and 0.3% Triton-X-100 for 1 h at room temperature. Primary antibody against LC3A/B (Cell Signaling, #4108) was used overnight at 4°C in PBS containing 1% of BSA and 0.3% Triton. Anti-goat IgG antibody conjugated to Alexa Fluor 568 was used for 1 h at RT then rinsed several times; cells cultures on glass slides were mounted with Vectashield mounting medium containing DAPI, and analysed with a confocal microscope (Nikon).

### Isolation of glomerular cells

Glomeruli were isolated from six WT and six *Timp3*^*−/−*^ mice by differential sieving. Briefly, kidneys were coarsely minced; tissue fragments were passed through a no. 100 mesh sieve (Falcon) with a sterile rubber stopper and rinsed several times with PBS. The suspension was then sequentially passed through no. 70 and no. 40 sieves. Glomeruli were digested with type IV collagenase (Sigma, St. Louis, MO, USA) for 40 min at 37°C before plating on gelatin coated-tissue culture dishes. Mesangial cells (WT and *T3*^*ko*^ pMes) were cultured in RPMI with 20% FCS in a 37°C humidified 10% CO_2_ incubator. After the first passages, they were shifted to 10% FCS culture medium.

### Statistical analysis

Results of the experimental studies are mean ± SD, as indicated. Statistical analysis was performed using one-way ANOVA or unpaired Student's *t*-test as appropriate. Values of *p* < 0.05 were considered statistically different.

For more detailed materials and methods see the Supporting Information.

## Supplementary material

As a service to our authors and readers, this journal provides supporting information supplied by the authors. Such materials are peer reviewed and may be re-organized for online delivery, but are not copy-edited or typeset. Technical support issues arising from supporting information (other than missing files) should be addressed to the authors.
