# Supplementary material for: Loss of TIMP3 underlies diabetic nephropathy via FoxO1/STAT1 interplay
Source: EMBO Mol Med. 2013 Feb 12;5(3):441–55. doi: 10.1002/emmm.201201475 (PMC3598083; doi:10.1002/emmm.201201475)

Full unedited gel for Figure 1D (TIMP3)

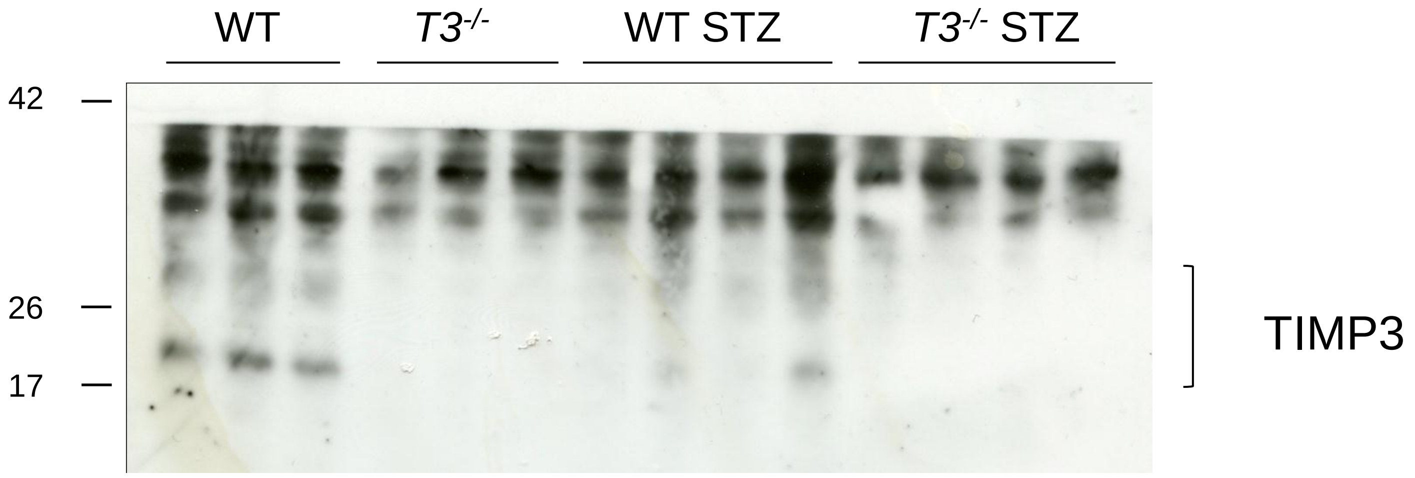

Full unedited gel for Figure 1D (TUBULIN)

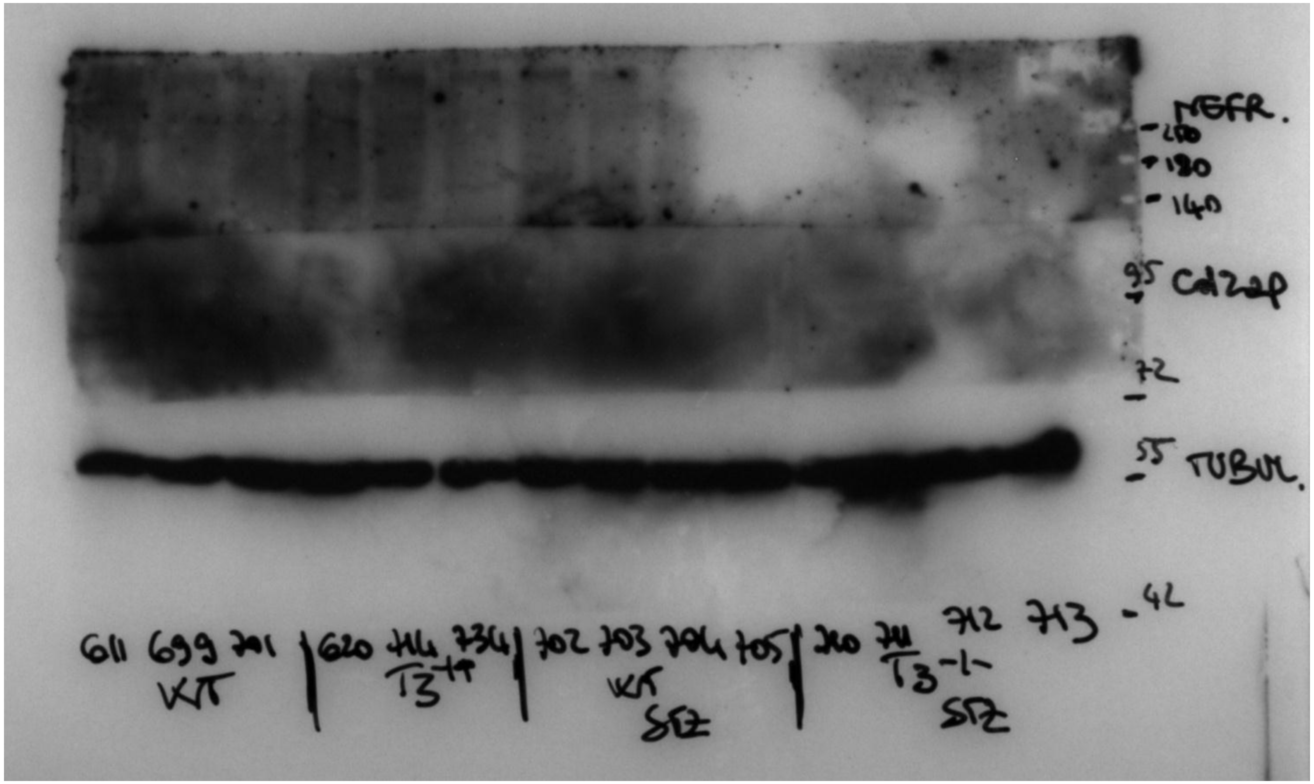

Full unedited gel for Figure 1G (TNF-α)

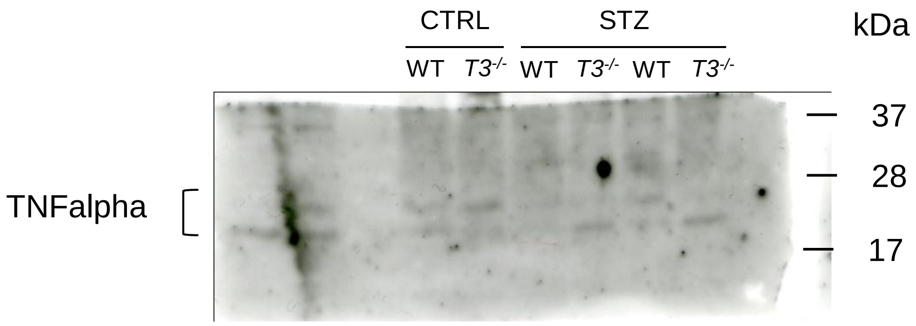

Full unedited gel for Figure 1G (ACTIN)

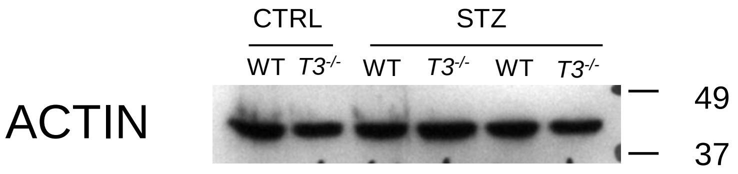

Supplement: Supplementary file 1 [file emmm0005-0441-SD1.pdf]
