# Supplementary material for: Loss of TIMP3 underlies diabetic nephropathy via FoxO1/STAT1 interplay
Source: EMBO Mol Med. 2013 Feb 12;5(3):441–55. doi: 10.1002/emmm.201201475 (PMC3598083; doi:10.1002/emmm.201201475)

Full unedited gel for Figure 2C (phospho-AKT)

phospho-AKT

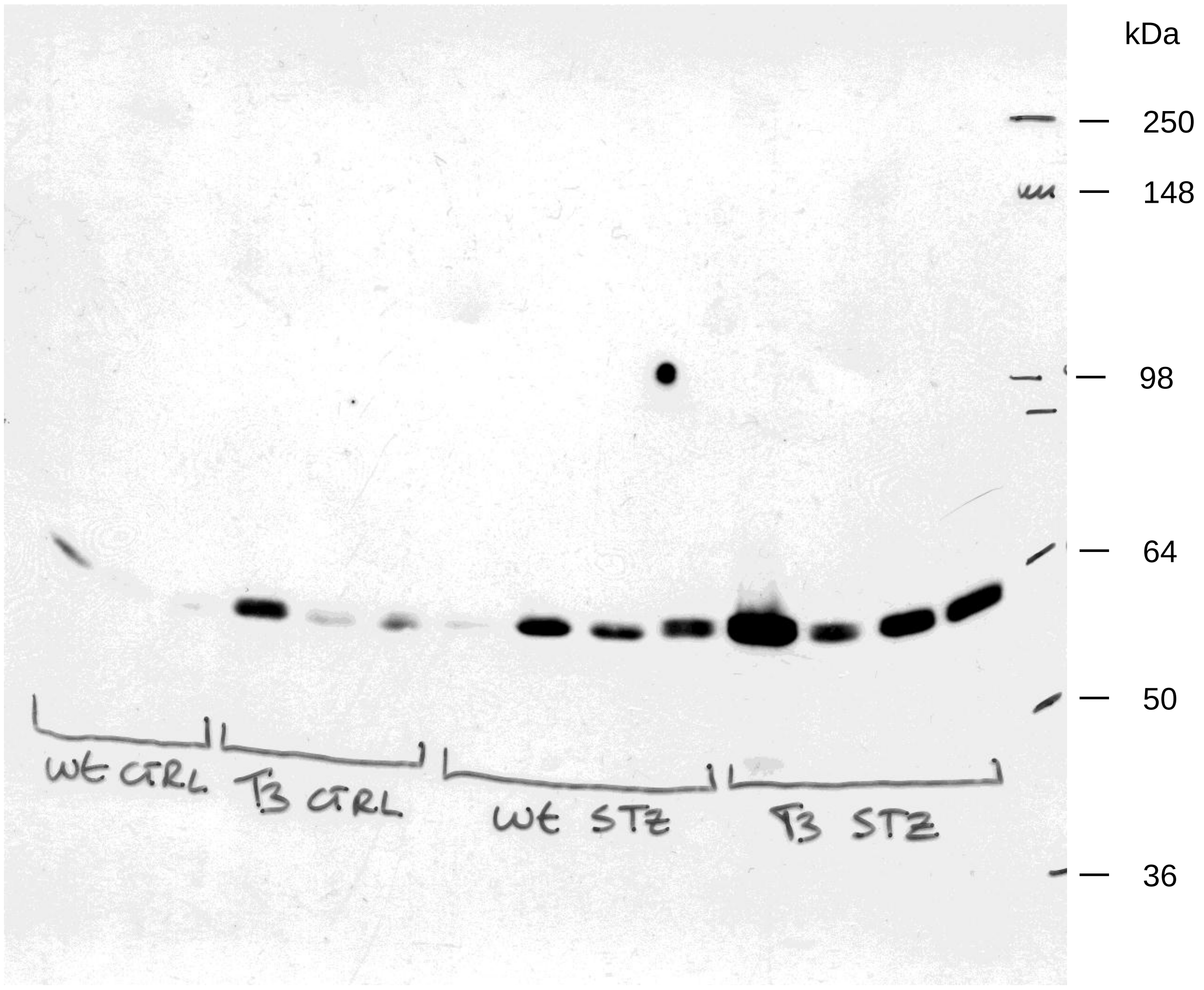

Full unedited gel for Figure 2C (total AKT)

total AKT

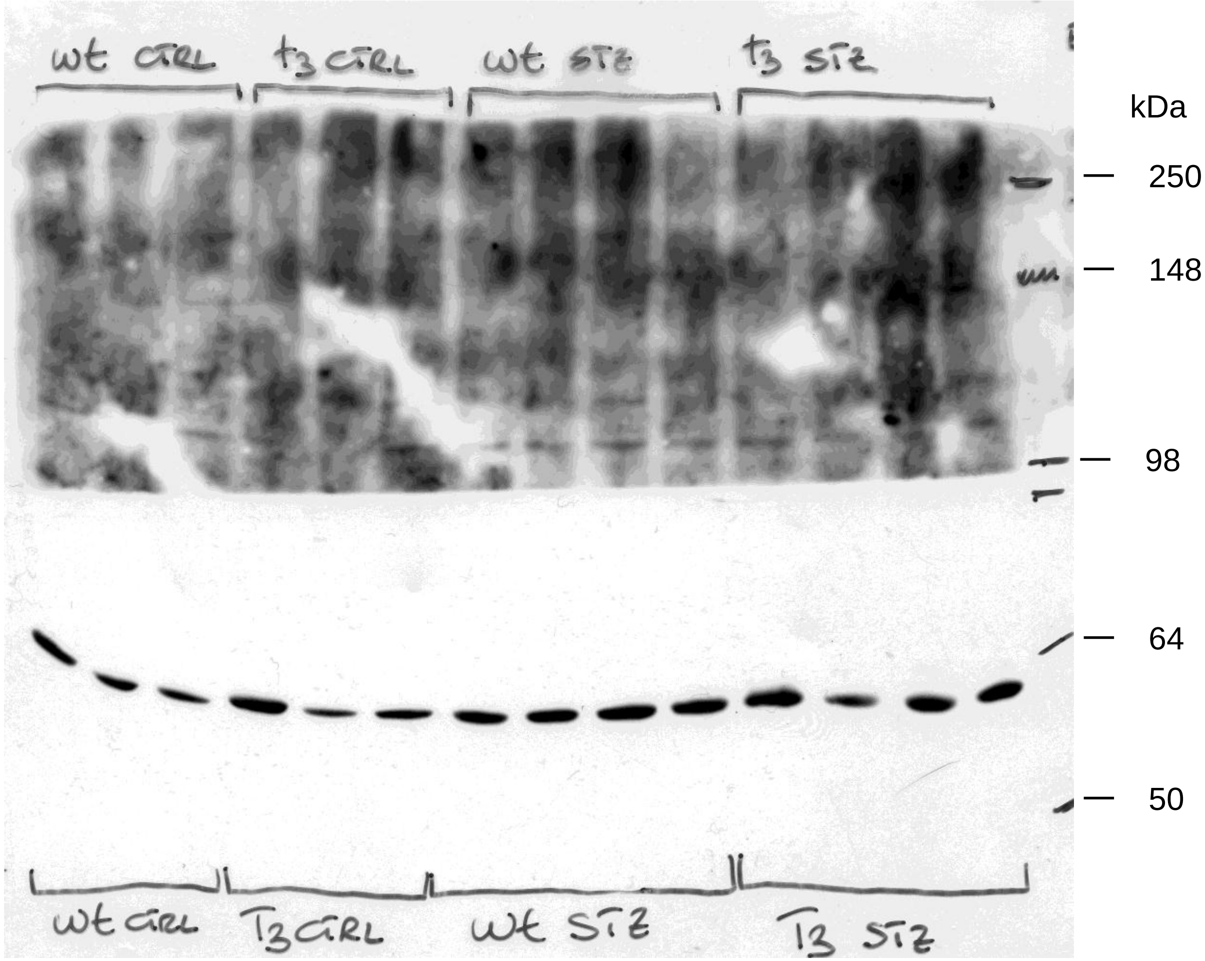

Full unedited gel for Figure 2C (phospho-ERK)

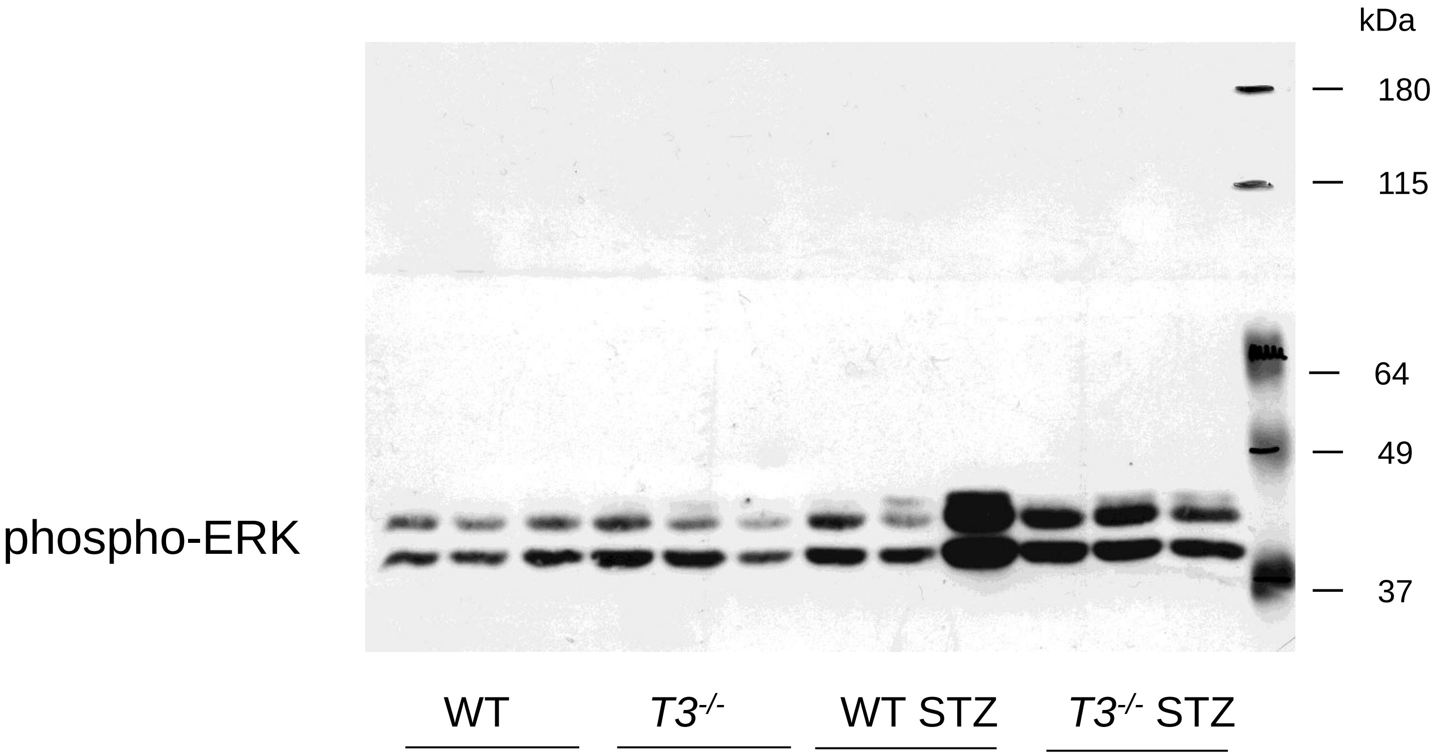

Full unedited gel for Figure 2C (total-ERK)

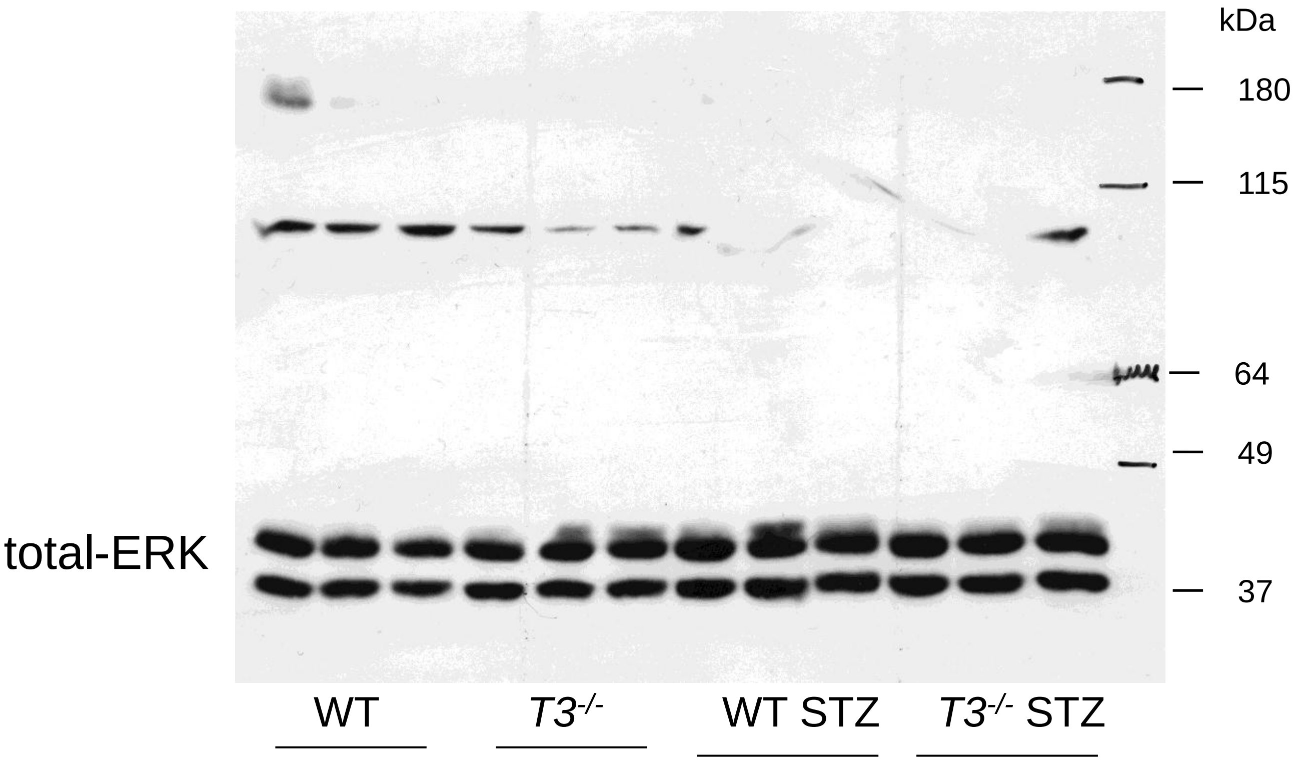

Full unedited gel for Figure 2C (phospho-EGFR)

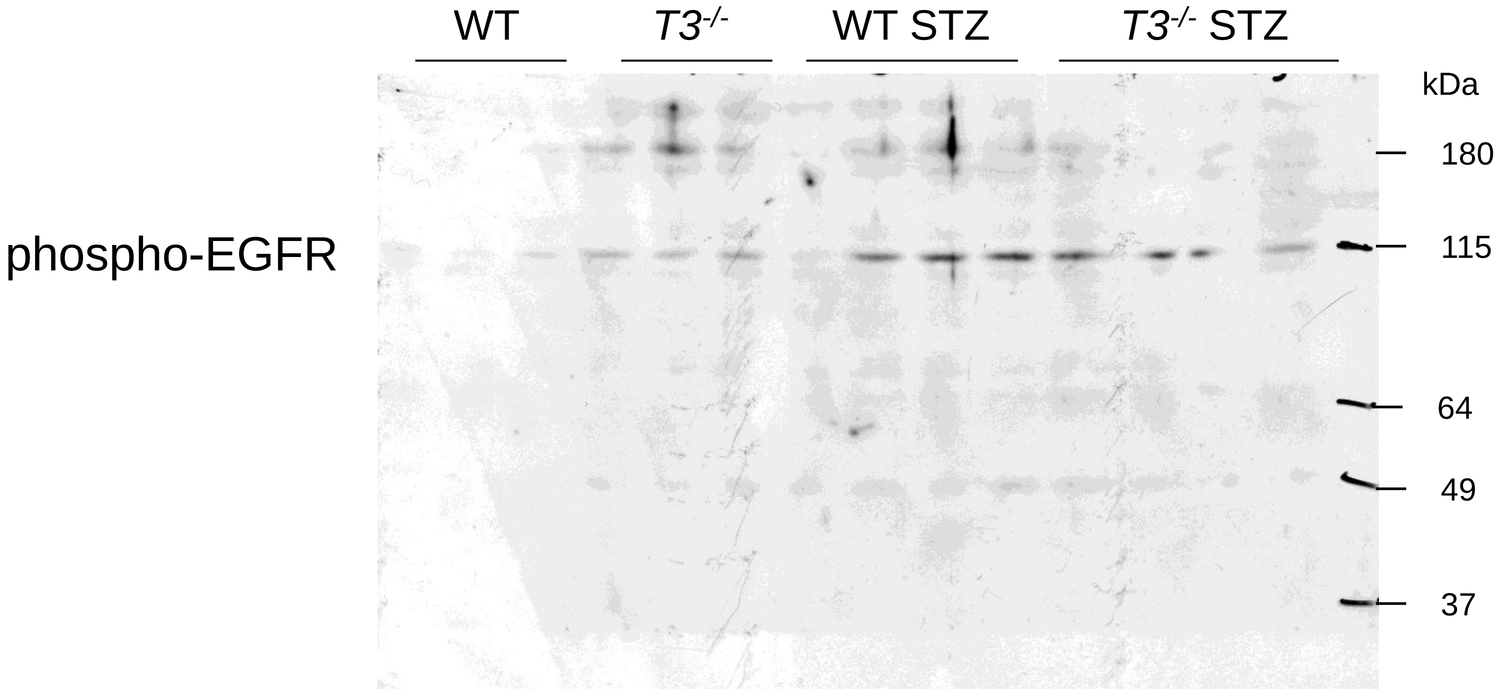

Full unedited gel for Figure 2C (total-EGFR)

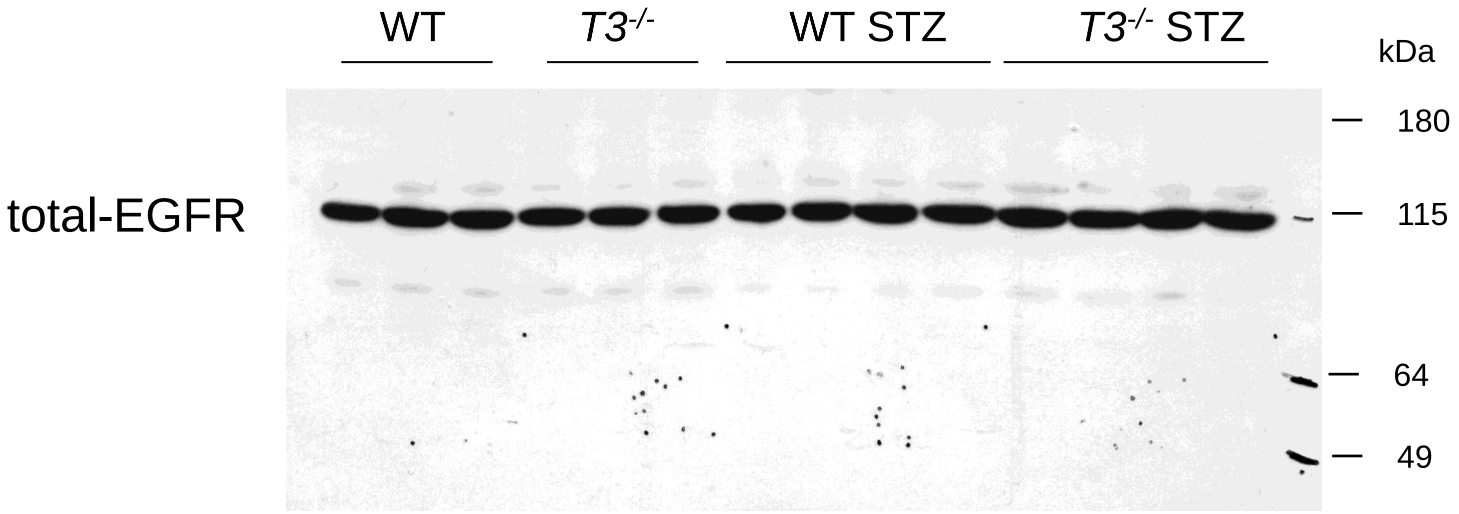

Supplement: Supplementary file 2 [file emmm0005-0441-SD2.pdf]
