# Supplementary material for: Loss of TIMP3 underlies diabetic nephropathy via FoxO1/STAT1 interplay
Source: EMBO Mol Med. 2013 Feb 12;5(3):441–55. doi: 10.1002/emmm.201201475 (PMC3598083; doi:10.1002/emmm.201201475)

Full unedited gel for Figure 3B (total Foxo and tubulin)

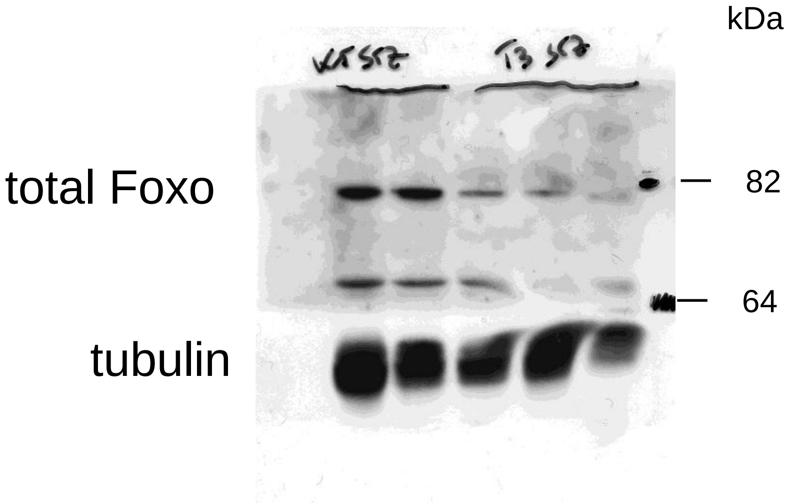

Full unedited gel for Figure 3B (nuclear Foxo)

nuclear Foxo

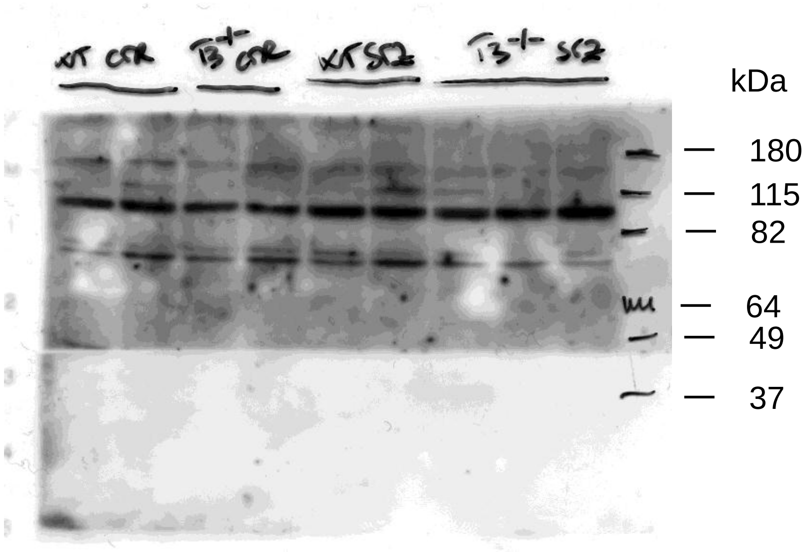

Full unedited gel for Figure 3B (Topoisomerase I)

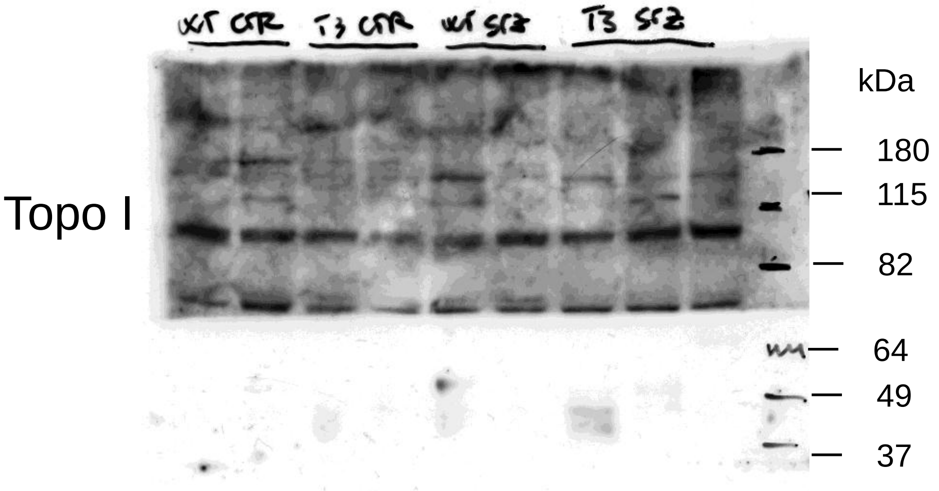

Full unedited gel for Figure 3B (cytoplasmic Foxo)

cyt Foxo

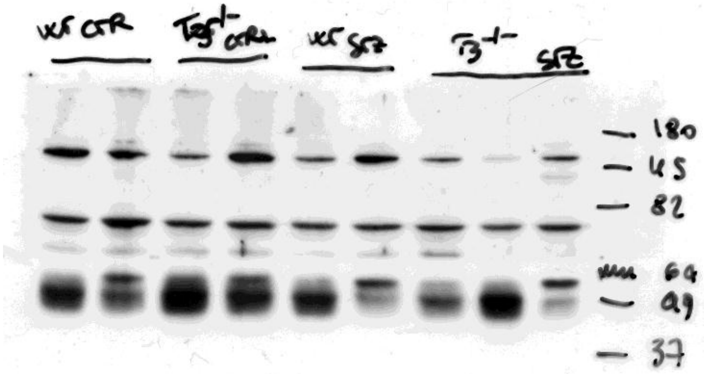

Supplement: Supplementary file 3 [file emmm0005-0441-SD3.pdf]
