# Supplementary material for: Loss of TIMP3 underlies diabetic nephropathy via FoxO1/STAT1 interplay
Source: EMBO Mol Med. 2013 Feb 12;5(3):441–55. doi: 10.1002/emmm.201201475 (PMC3598083; doi:10.1002/emmm.201201475)
Supplement: Supplementary file 4 [file emmm0005-0441-SD4.pdf]

Full unedited gel for Figure 4A (TIMP3, long exposure)

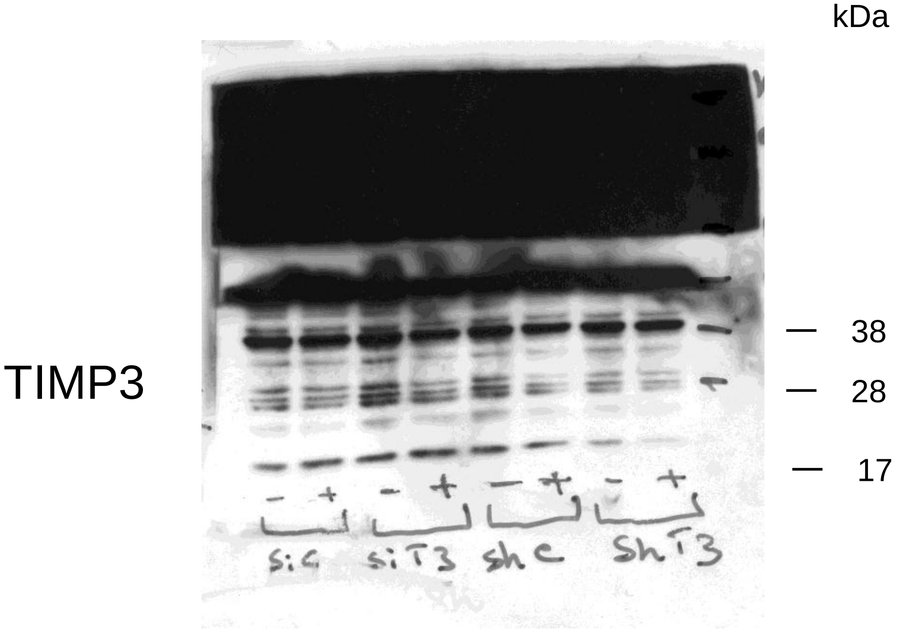

Full unedited gel for Figure 4A (tubulin, short exposure)

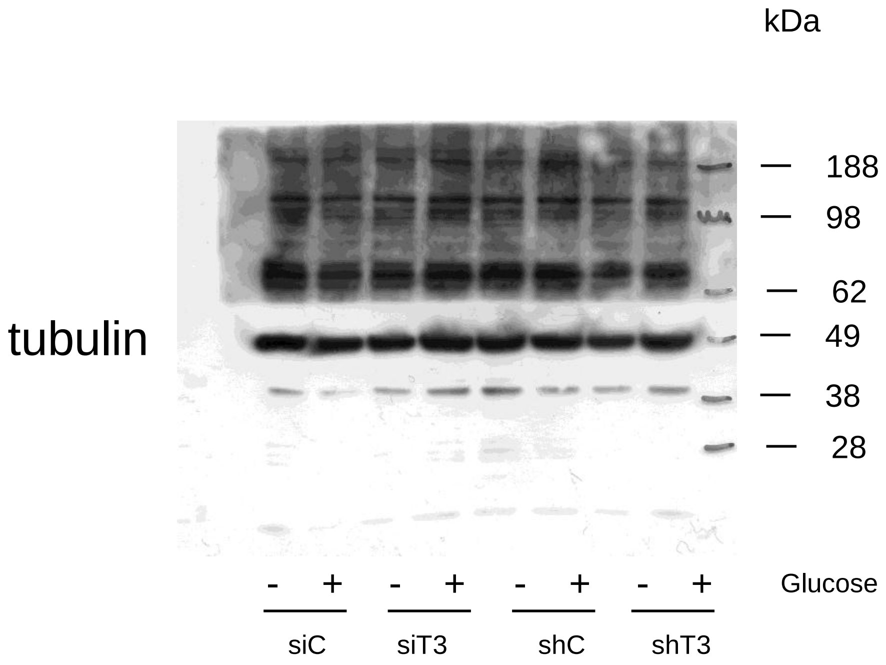

Full unedited gel for Figure 4D (ATG5)

Ctrl MES13	*T3*^{kd} MES13

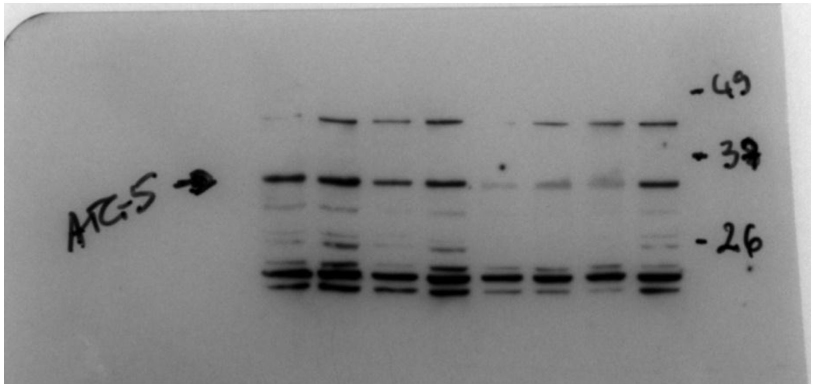

-	+	-	-	-	+	-	-	Glucose
-	-	+	-	-	-	+	-	Mannitol
-	-	-	+	-	-	-	+	Starvation

Full unedited gel for Figure 4D (ATG8)

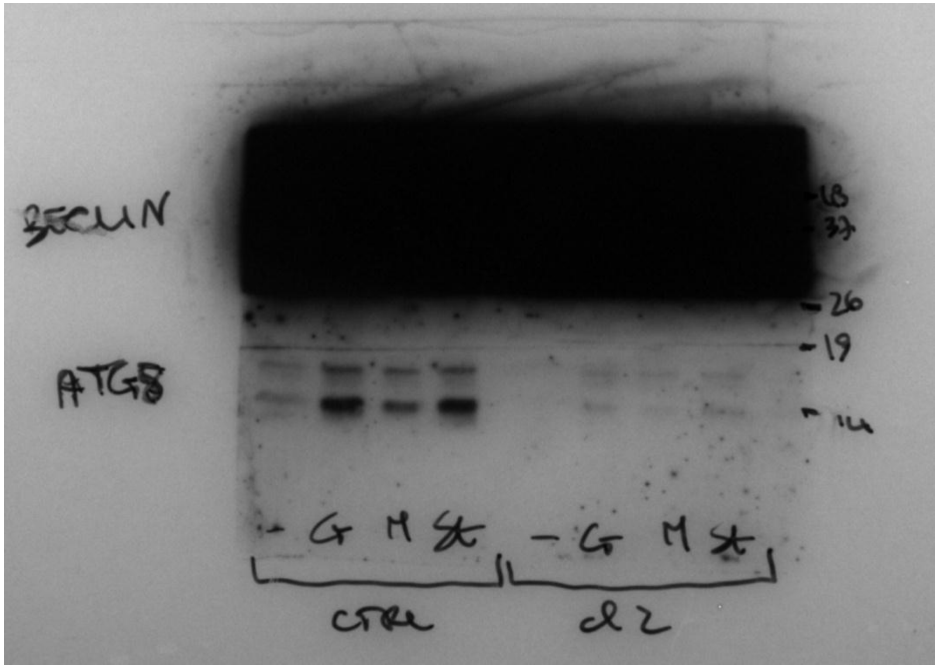

Full unedited gel for Figure 4D (LC3)

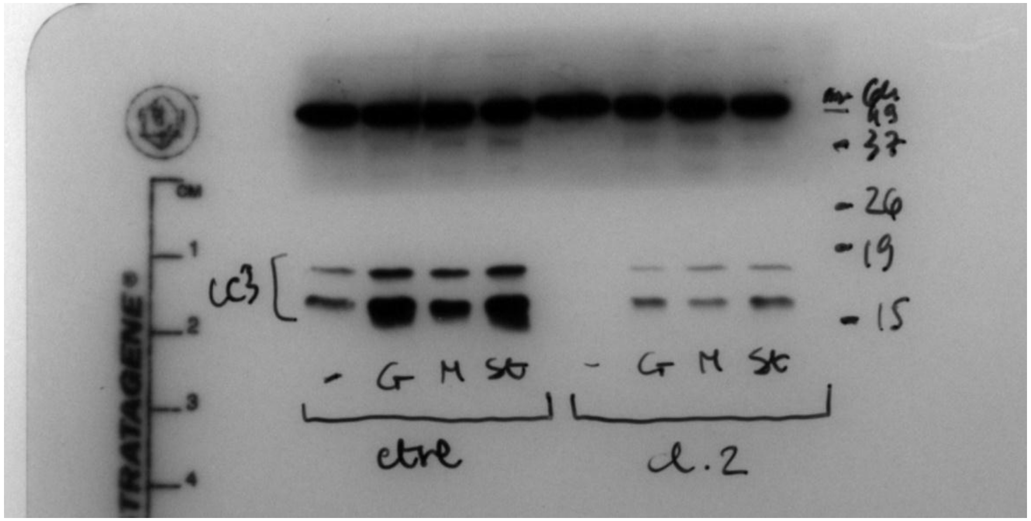

Full unedited gel for Figure 4D (BECLIN)

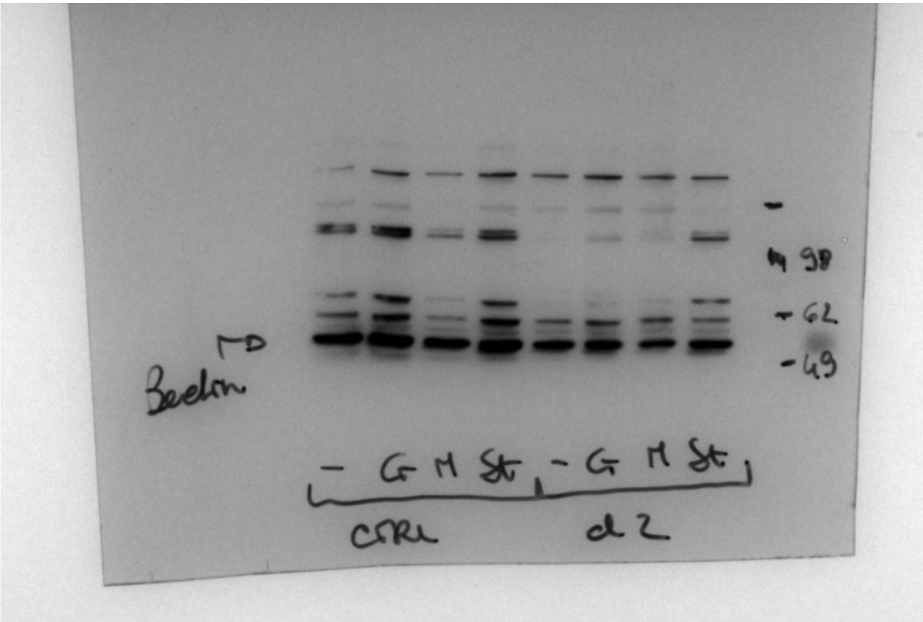

Full unedited gel for Figure 4D (ACTIN)

Ctrl MES13 — *T3*^{kd} MES13

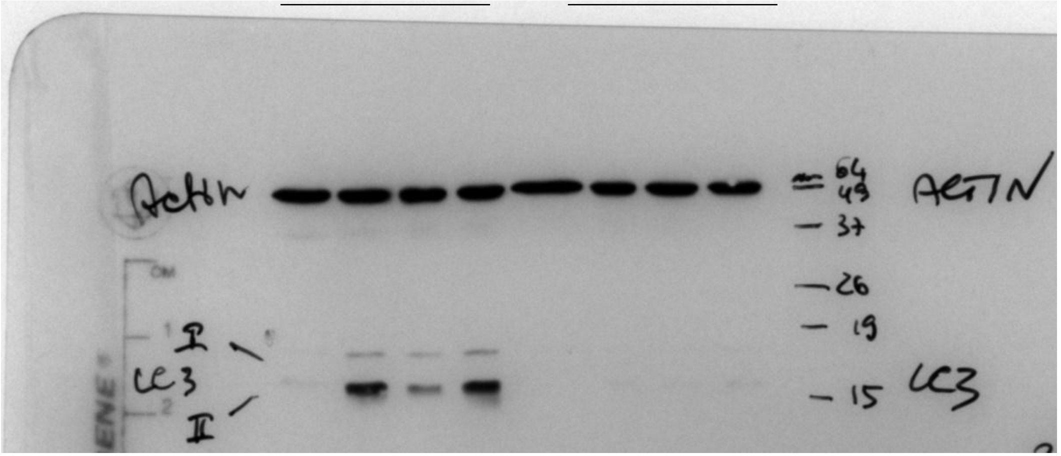

-	+	-	-	-	+	-	-	Glucose
-	-	+	-	-	-	+	-	Mannitol
-	-	-	+	-	-	-	+	Starvation
