# Supplementary material for: Loss of TIMP3 underlies diabetic nephropathy via FoxO1/STAT1 interplay
Source: EMBO Mol Med. 2013 Feb 12;5(3):441–55. doi: 10.1002/emmm.201201475 (PMC3598083; doi:10.1002/emmm.201201475)
Supplement: Supplementary file 5 [file emmm0005-0441-SD5.pdf]

Full unedited gel for Figure 5A (total Foxo)

total Foxo

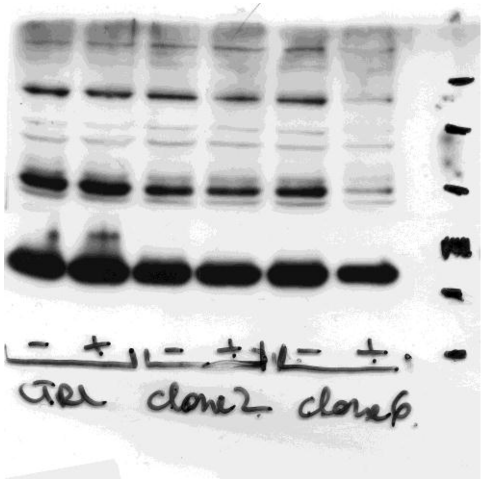

kDa
180
115
82
64
49
37

Full unedited gel for Figure 5A (nuclear Foxo)

nuclear Foxo

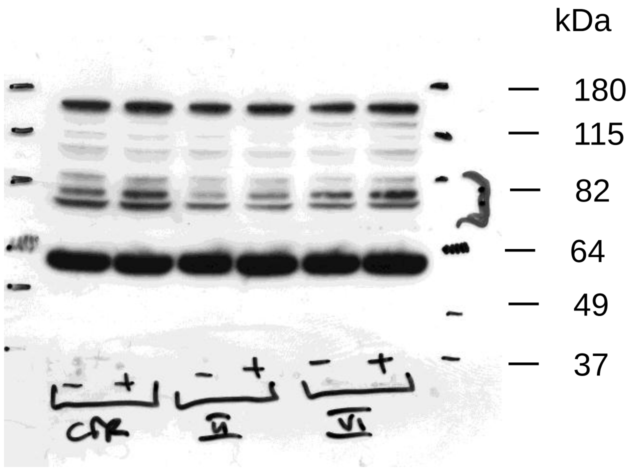

Full unedited gel for Figure 5A (Topoisomerase I)

Topo I

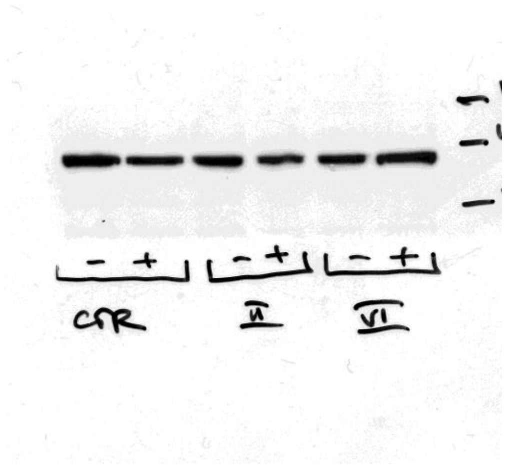

kDa
180
115
82

Full unedited gel for Figure 5A (cytoplasmic Foxo)

cyt Foxo

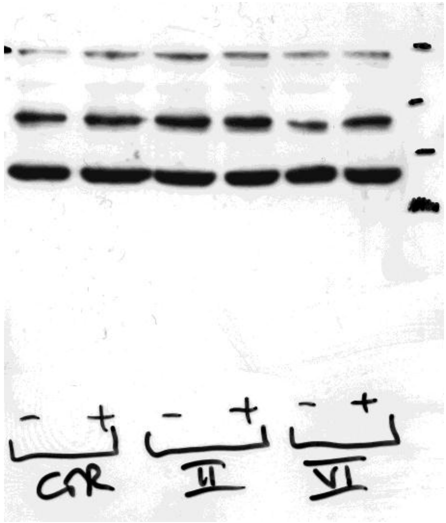

kDa

180

115

82

Full unedited gel for Figure 5A (tubulin)

tubulin

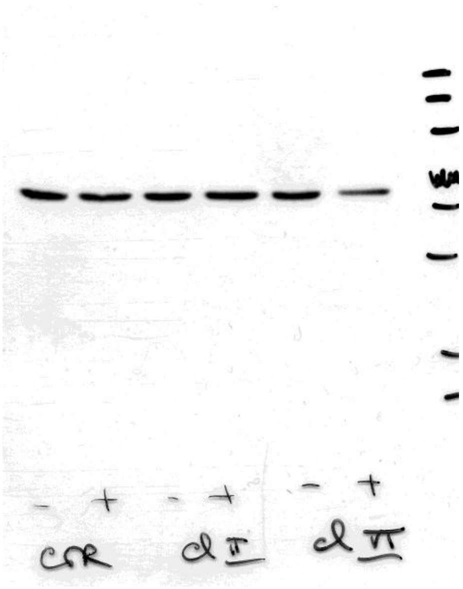

kDa

180
115
82
64
49
37

Full unedited gel for Figure 5B (acetyl-Foxo, long exposure)

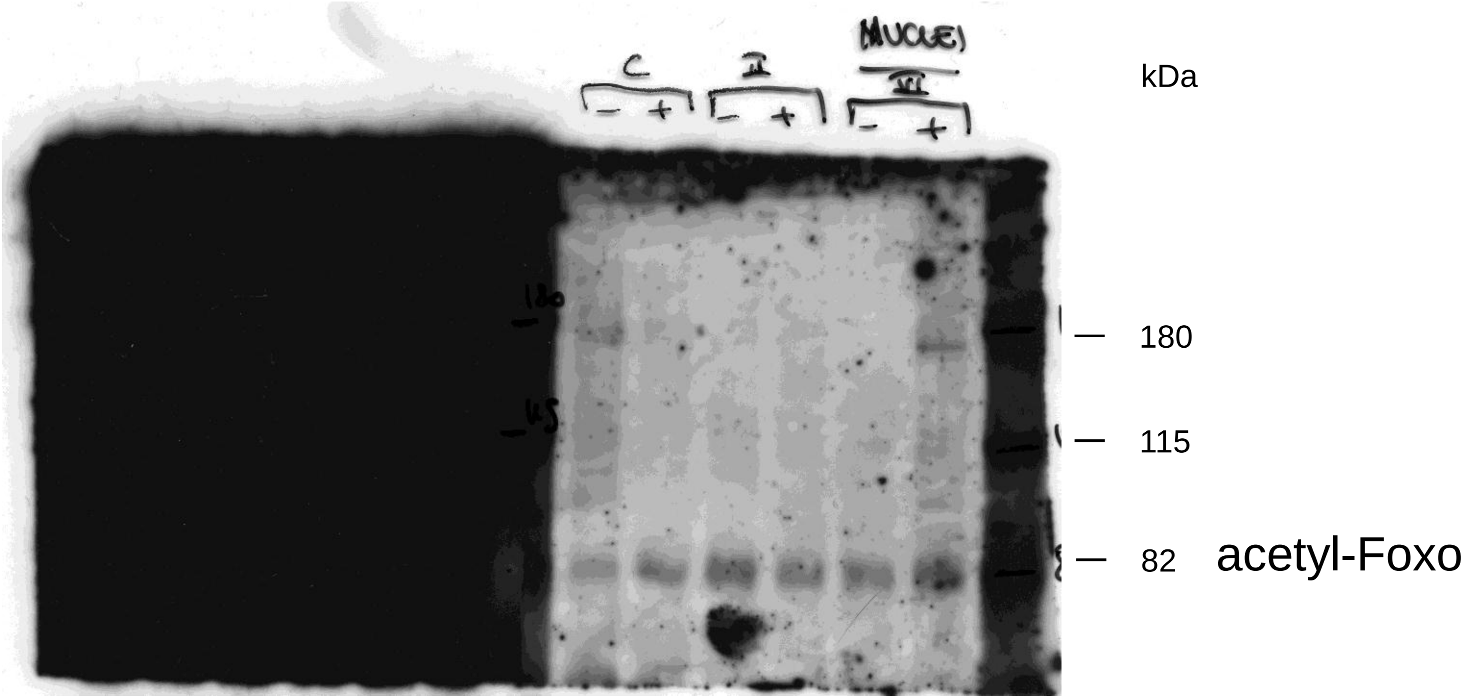

Full unedited gel for Figure 5B (total Foxo, short exposure)

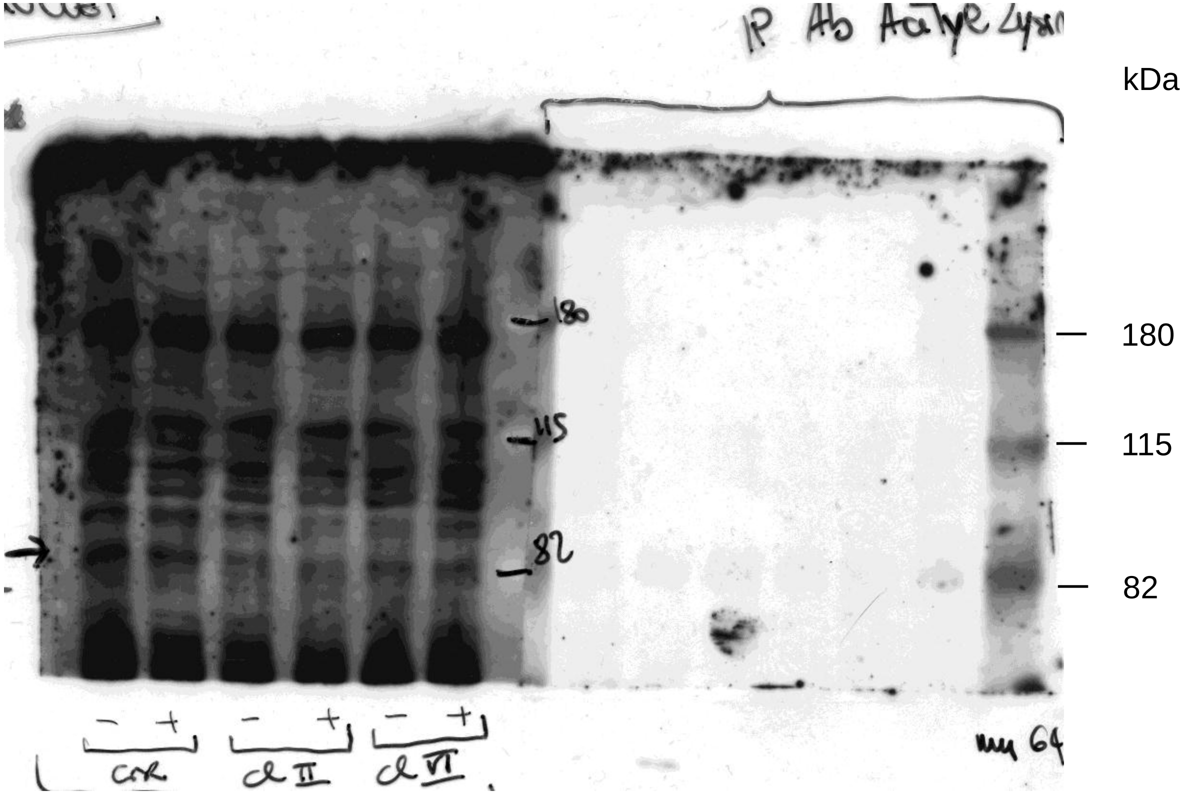

kDa

180

115

total Foxo

82

Full unedited gel for Figure 5B (Topoisomerase I)

Topo I

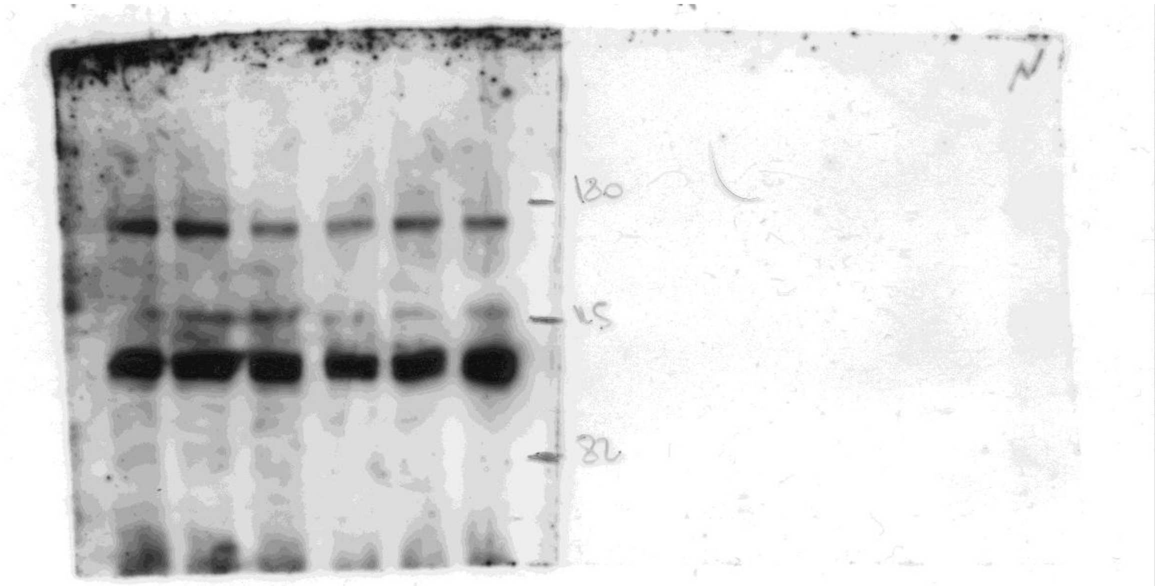

- + - + - +

Ctrl cl.II cl.VI

Full unedited gel for Figure 5C (Map1LC3a CHIP)

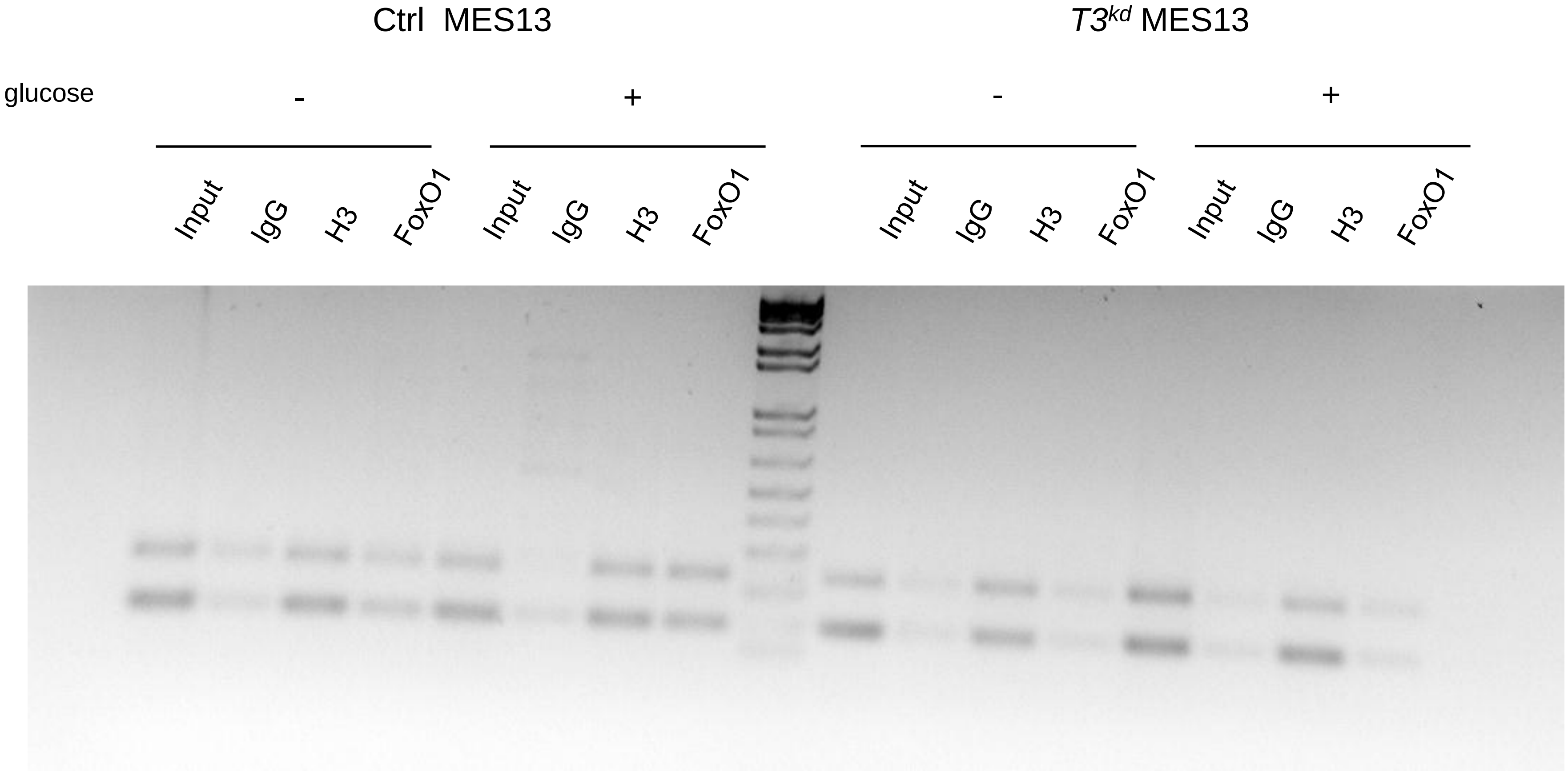

Marker = 1Kb ladder

Full unedited gel for Figure 5C (Gabarapl1 CHIP)

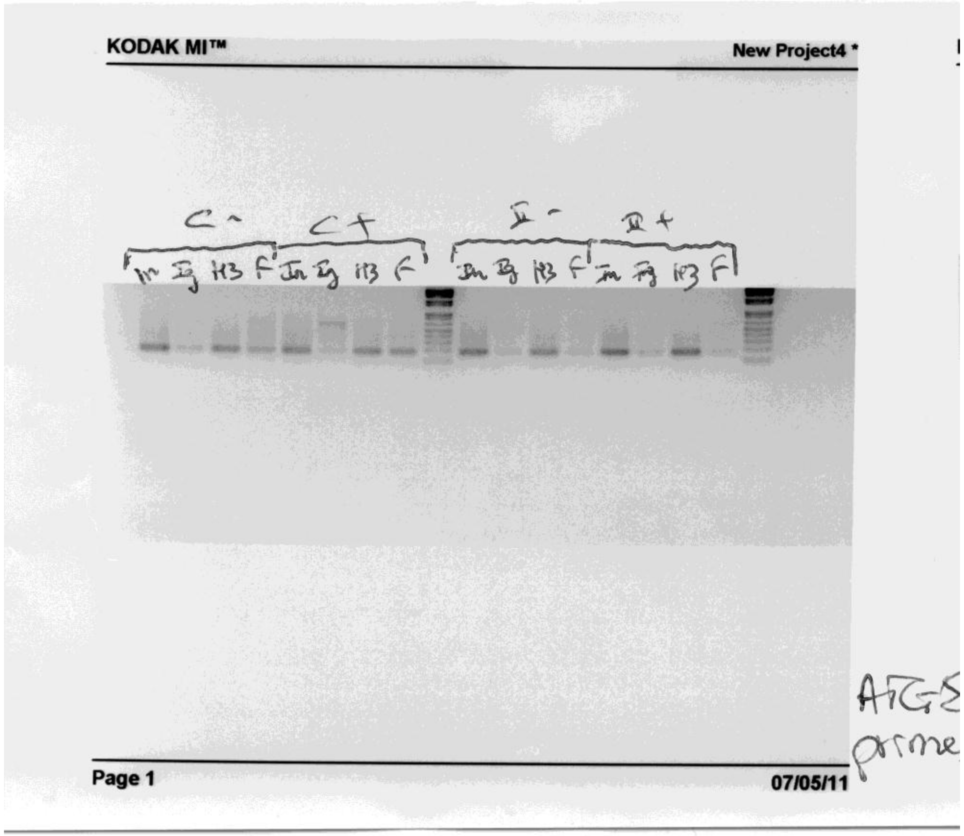

Marker = 1Kb ladder
