# Supplementary material for: Loss of TIMP3 underlies diabetic nephropathy via FoxO1/STAT1 interplay
Source: EMBO Mol Med. 2013 Feb 12;5(3):441–55. doi: 10.1002/emmm.201201475 (PMC3598083; doi:10.1002/emmm.201201475)

Full unedited gel for Figure 6B (TIMP3)

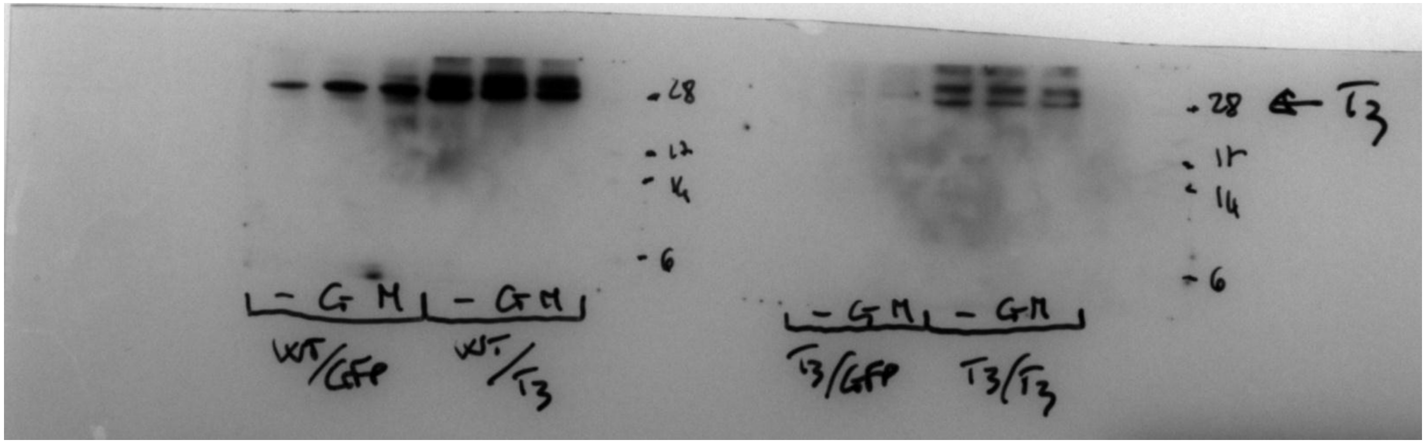

Full unedited gel for Figure 6B (FOXO1)

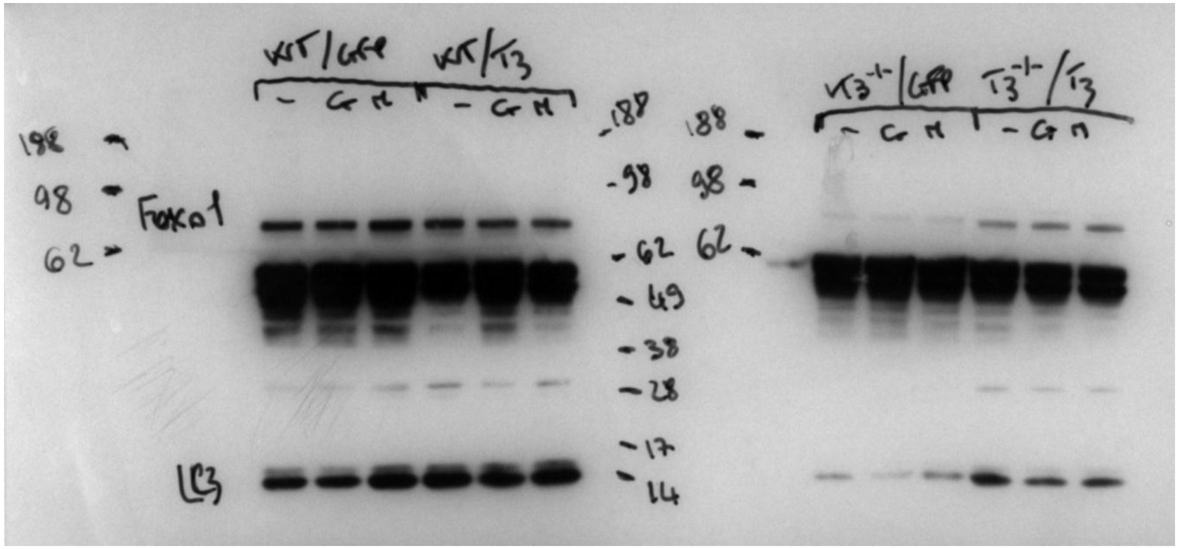

Full unedited gel for Figure 6B (ATG5)

WT pMes — AdGFP, AdT3

T3^{ko} pMes — AdGFP, AdT3

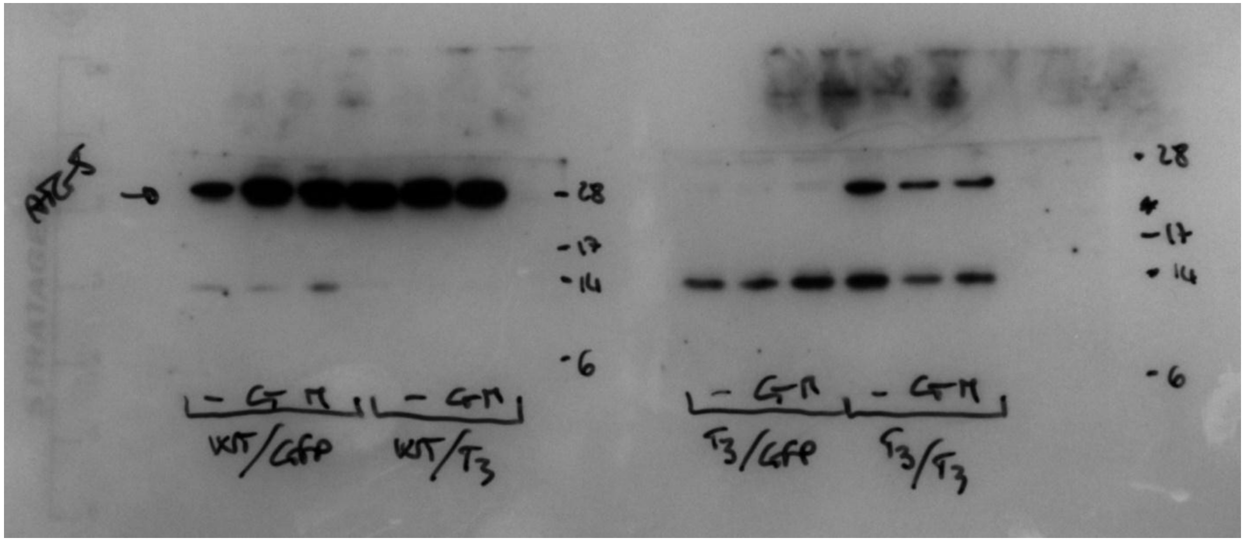

	WT pMes AdGFP			WT pMes AdT3			*T3*^{ko} pMes AdGFP			*T3*^{ko} pMes AdT3		
Glucose	-	+	-	-	+	-	-	+	-	-	+	-
Mannitol	-	-	+	-	-	+	-	-	+	-	-	+

Full unedited gel for Figure 6B (ATG8)

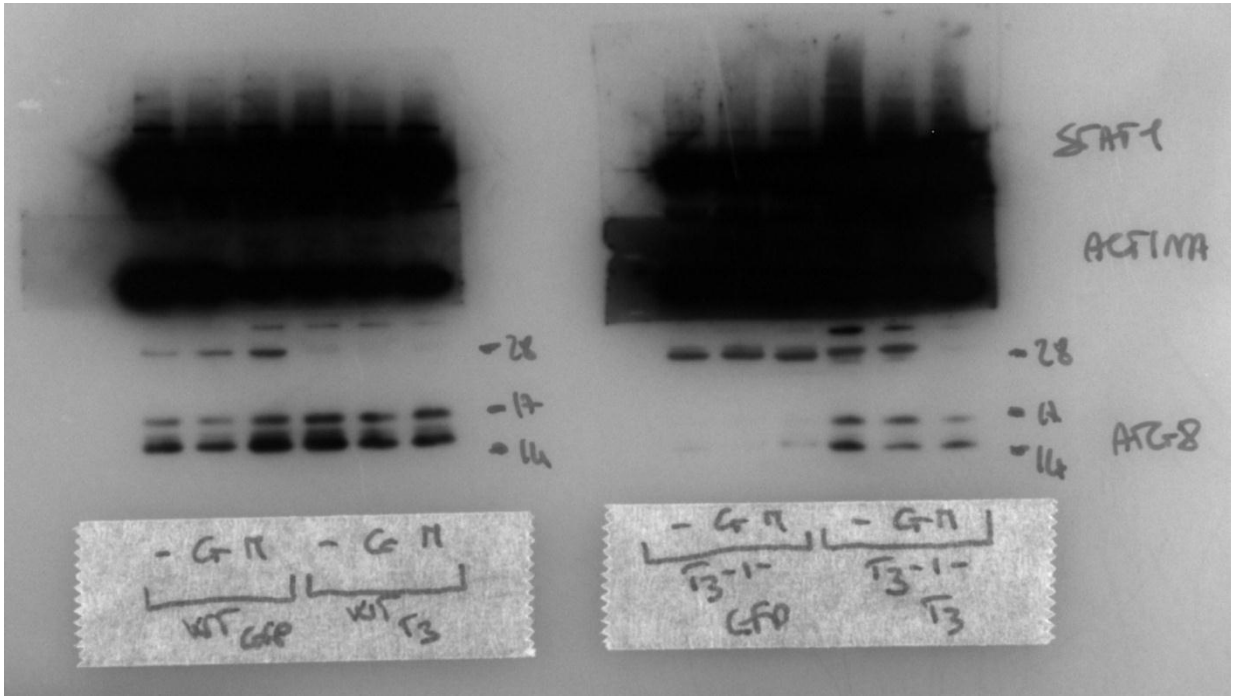

Full unedited gel for Figure 6B (BECLIN)

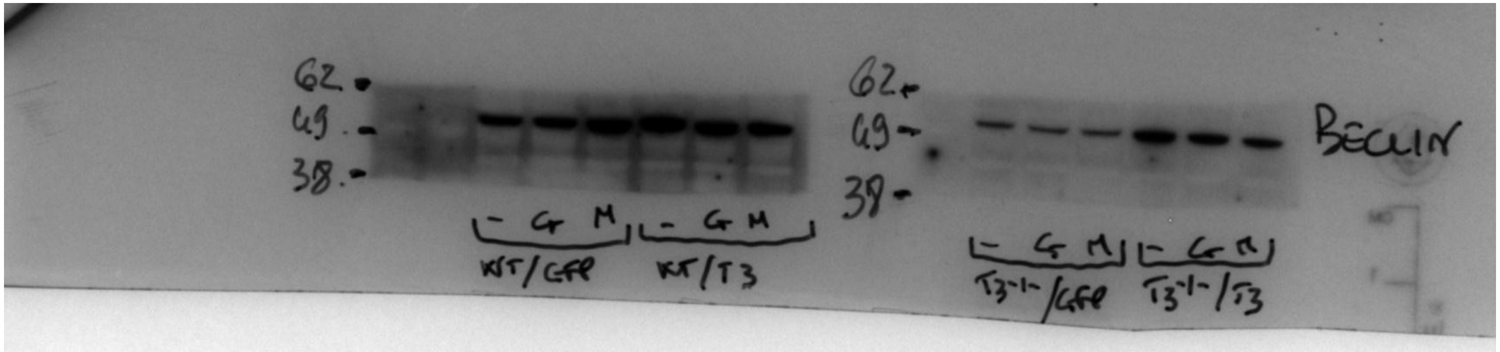

Full unedited gel for Figure 6B (ACTIN)

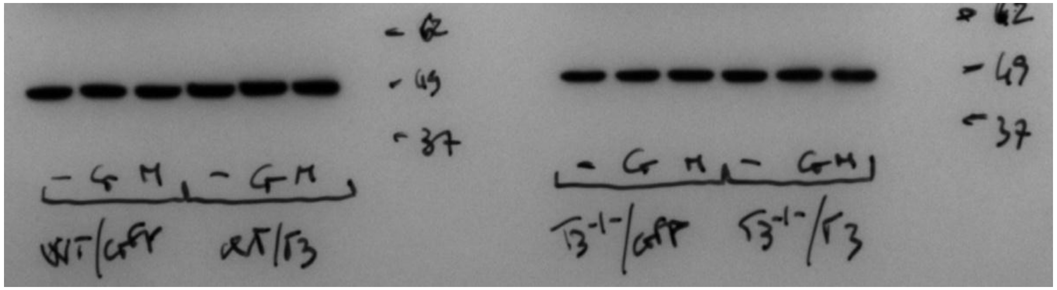

Full unedited gel for Figure 6B (LC3)

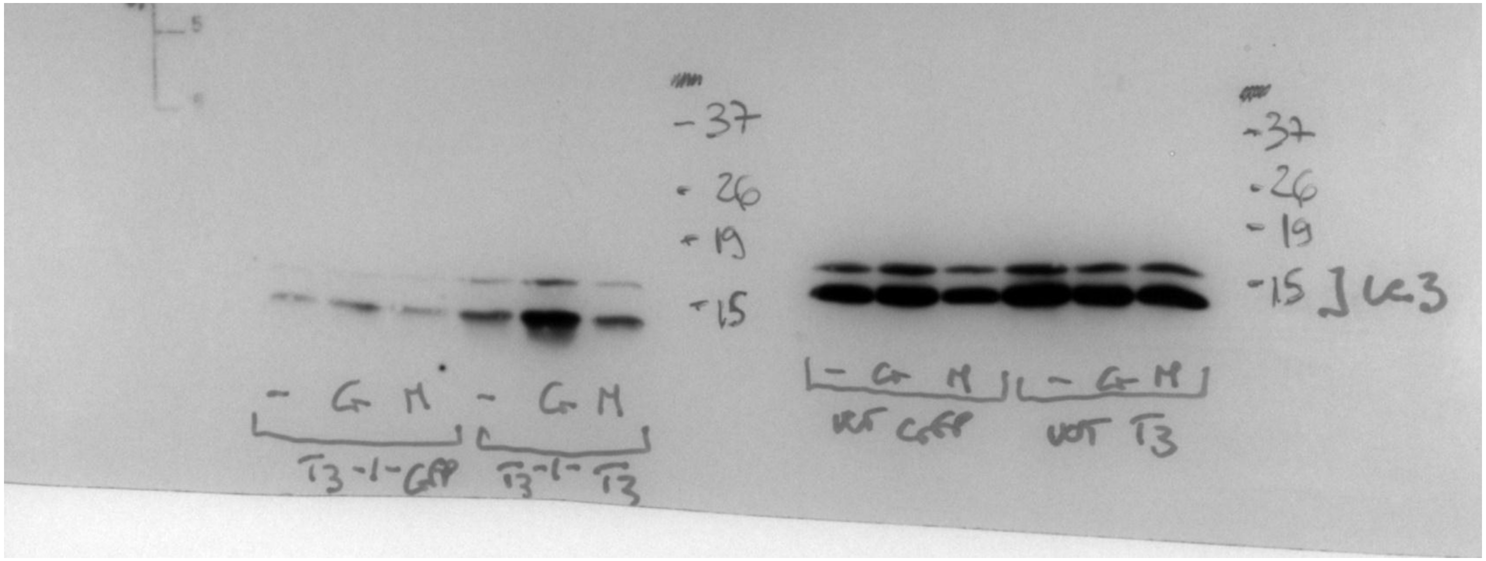

Full unedited gel for Figure 6B (ACTIN, 15%)

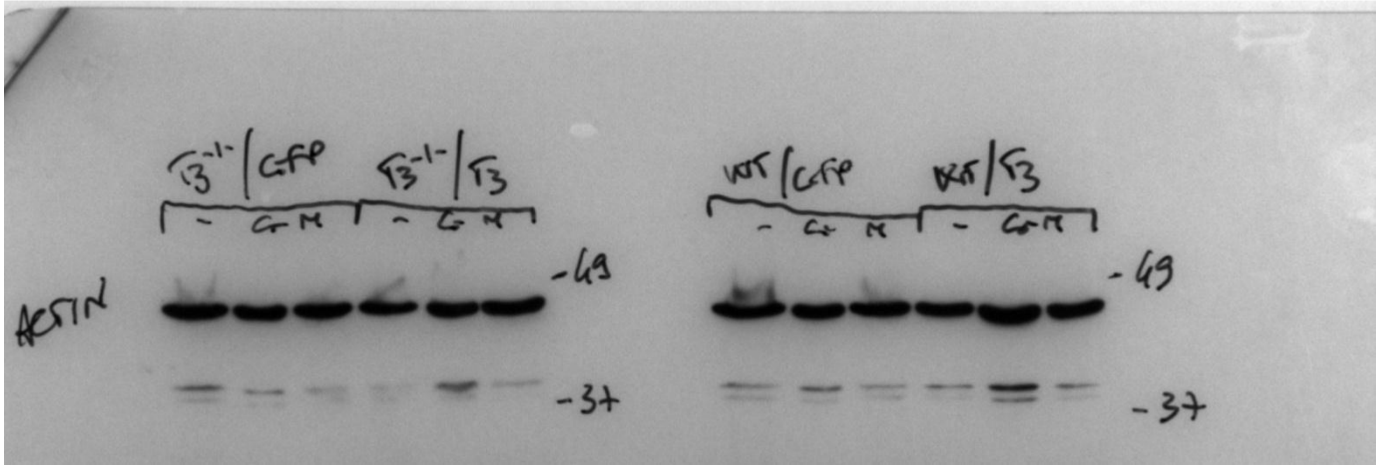

Supplement: Supplementary file 6 [file emmm0005-0441-SD6.pdf]
