# Supplementary material for: Loss of TIMP3 underlies diabetic nephropathy via FoxO1/STAT1 interplay
Source: EMBO Mol Med. 2013 Feb 12;5(3):441–55. doi: 10.1002/emmm.201201475 (PMC3598083; doi:10.1002/emmm.201201475)

Full unedited gel for Figure 7B (phospho-STAT1)

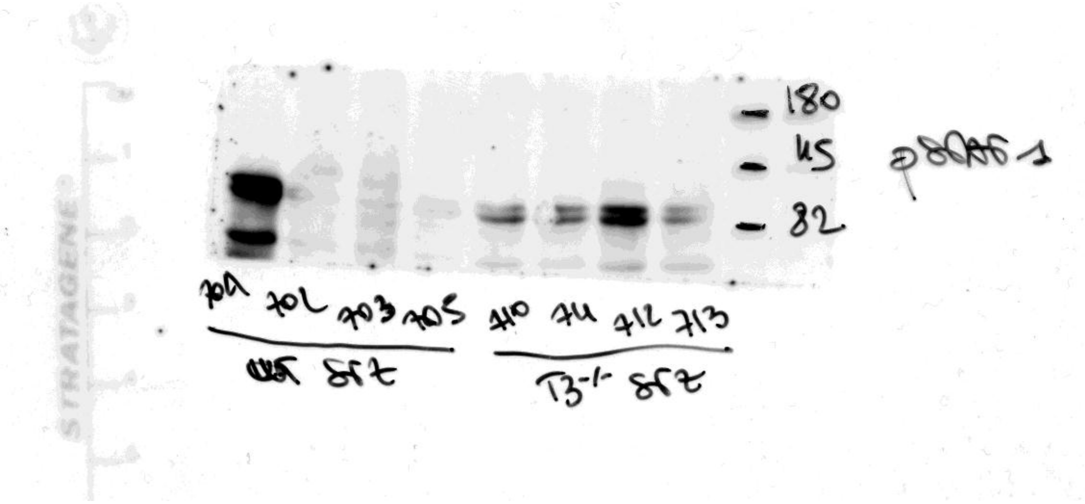

Full unedited gel for Figure 7B (total STAT1)

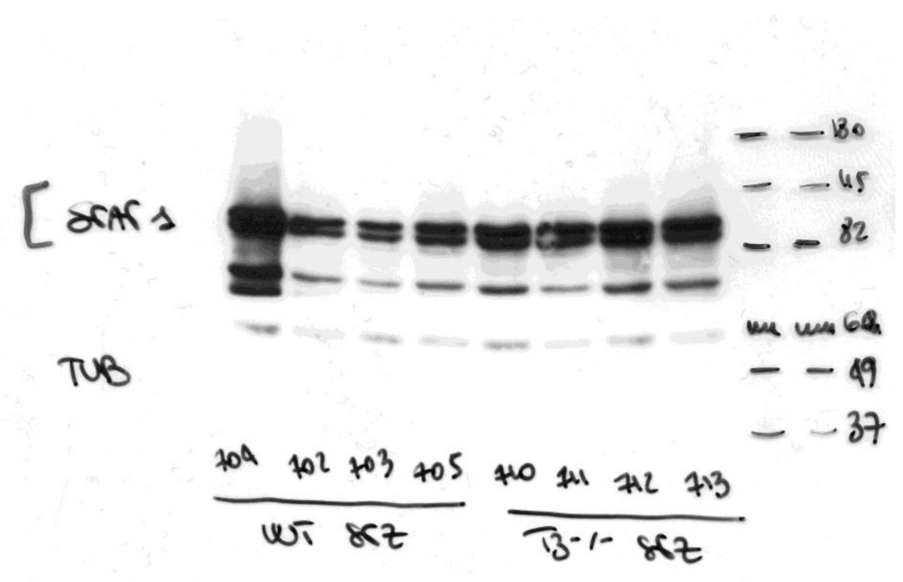

Full unedited gel for Figure 7B (tubulin)

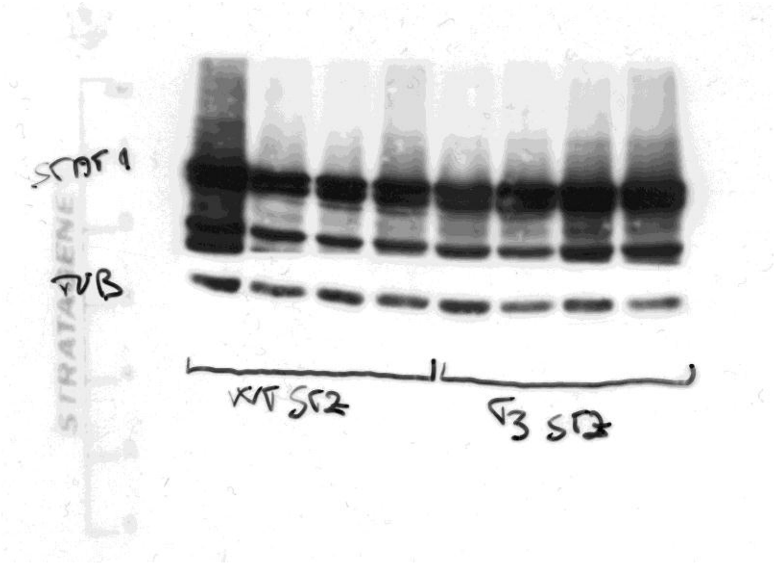

Full unedited gel for Figure 7E (STAT1 and ACTIN)

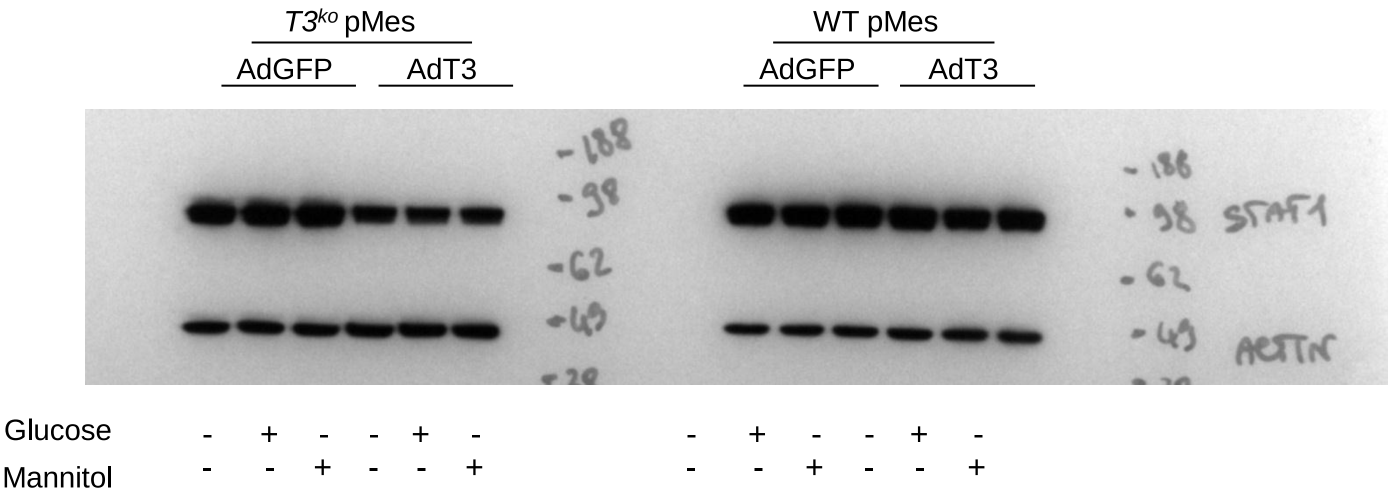

Full unedited gel for Figure 7F (STAT1)

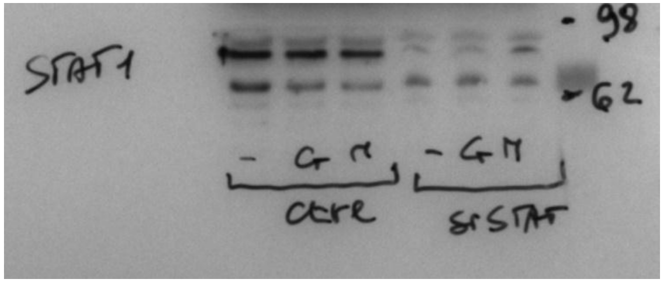

Full unedited gel for Figure 7F (ACTIN)

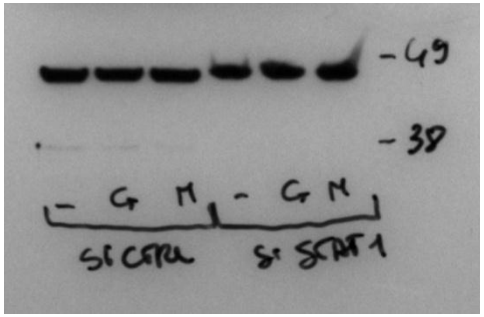

Supplement: Supplementary file 7 [file emmm0005-0441-SD7.pdf]
