# Supplementary material for: Loss of TIMP3 underlies diabetic nephropathy via FoxO1/STAT1 interplay
Source: EMBO Mol Med. 2013 Feb 12;5(3):441–55. doi: 10.1002/emmm.201201475 (PMC3598083; doi:10.1002/emmm.201201475)
Supplement: Supplementary file 8 [file emmm0005-0441-SD8.pdf]

Supplemental Information

Loss of TIMP3 underlies diabetic nephropathy via FoxO1/STAT1 interplay

Loredana Fiorentino, Michele Cavalera, Stefano Menini, Valentina Marchetti Maria Mavilio, Marta Fabrizi, Francesca Conserva, Viviana Casagrande, Rossella Menghini, Paola Pontrelli, Ivan Arisi, Mara D'Onofrio, Davide Lauro, Rama Khokha, Domenico Accili, Giuseppe Pugliese, Loreto Gesualdo, Renato Lauro and Massimo Federici

Table of contents:

Figure S1: TIMP3 expression in kidney of normoglycemic and diabetic WT mice.

Figure S2: Renal features of WT and *Timp3*$^{-/-}$ mice after STZ treatment.

Figure S3: PAS staining of kidney sections from normoglycemic and diabetic WT and *Timp3*$^{-/-}$ mice.

Figure S4: Histological features of WT and *Timp3*$^{-/-}$ diabetic kidney.

Figure S5: MCP-1 expression in kidney of normoglycemic and diabetic WT and *Timp3*$^{-/-}$ mice.

Figure S6: F4/80 expression in kidney of normoglycemic and diabetic WT and *Timp3*$^{-/-}$ mice**.**

Figure S7: RAGE expression in kidney of normoglycemic and diabetic WT and *Timp3*$^{-/-}$ mice.

Figure S8: N-Carboxymethyl-lysine (CML) expression in kidney of normoglycemic and diabetic WT and *Timp3*$^{-/-}$ mice.

Figure S9: Nitrotyrosine expression in kidney of normoglycemic and diabetic WT and *Timp3*$^{-/-}$ mice.

Figure S10: NOX4 expression in kidney of normoglycemic and diabetic WT and *Timp3*$^{-/-}$ mice.

Figure S11: Expression profiling of kidneys from diabetic WT and *Timp3*$^{-/-}$ mice.

Figure S12: FOXO1 expression in kidney of normoglycemic and diabetic WT and *Timp3*$^{-/-}$ mice.

Figure S13: FOXO1 expression in the tubular compartment of diabetic WT and *Timp3*$^{-/-}$ mice.

Figure S14: Modulation of FOXO target genes.

Figure S15: Signal transduction pathways unchanged by TIMP3 deficiency.

Figure S16: Immunofluorescence for LC3A/B in *T3*^{kd} and control MES13 cells.

Figure S17: Characterization of *T3*^{kd} cells.

Figure S18: Rescue of FoxO1 and its targets in *T3*^{kd} MES13 cells infected with FoxO1-ADA adenovirus.

Figure S19: ADAM17 activity in *T3*^{ko} primary mesangial cells.

Figure S20: Immunofluorescence for LC3A/B in *T3*^{ko} and control pMes cells infected with GFP or TIMP3 adenovirus.

Figure S21: STAT1 expression in kidney of normoglycemic and diabetic WT and *Timp3*$^{-/-}$ mice.

Figure S22: STAT1 expression in kidney from healthy and diabetic subjects.

Figure S23: FOXO1 expression in kidney from healthy and diabetic subjects.

Supplemental Table 1. Metabolic parameters of WT and *Timp3*$^{-/-}$ mice used in the study.

Supplemental Table 2. Selection of differentially expressed transcripts in kidneys of diabetic WT and *Timp3*$^{-/-}$ mice.

Supplemental Table 3. Metabolic parameters of healthy subjects and diabetic patients.

Supplemental Table 4. List of mouse (A) and human (B) primers used for real time PCR.

Supplemental methods:

List of mouse Real time PCR primers.

Animal models and induction of diabetes.

Histological analysis and quantification of renal lesions.

Supplemental Figures:

Supplemental Figure 1

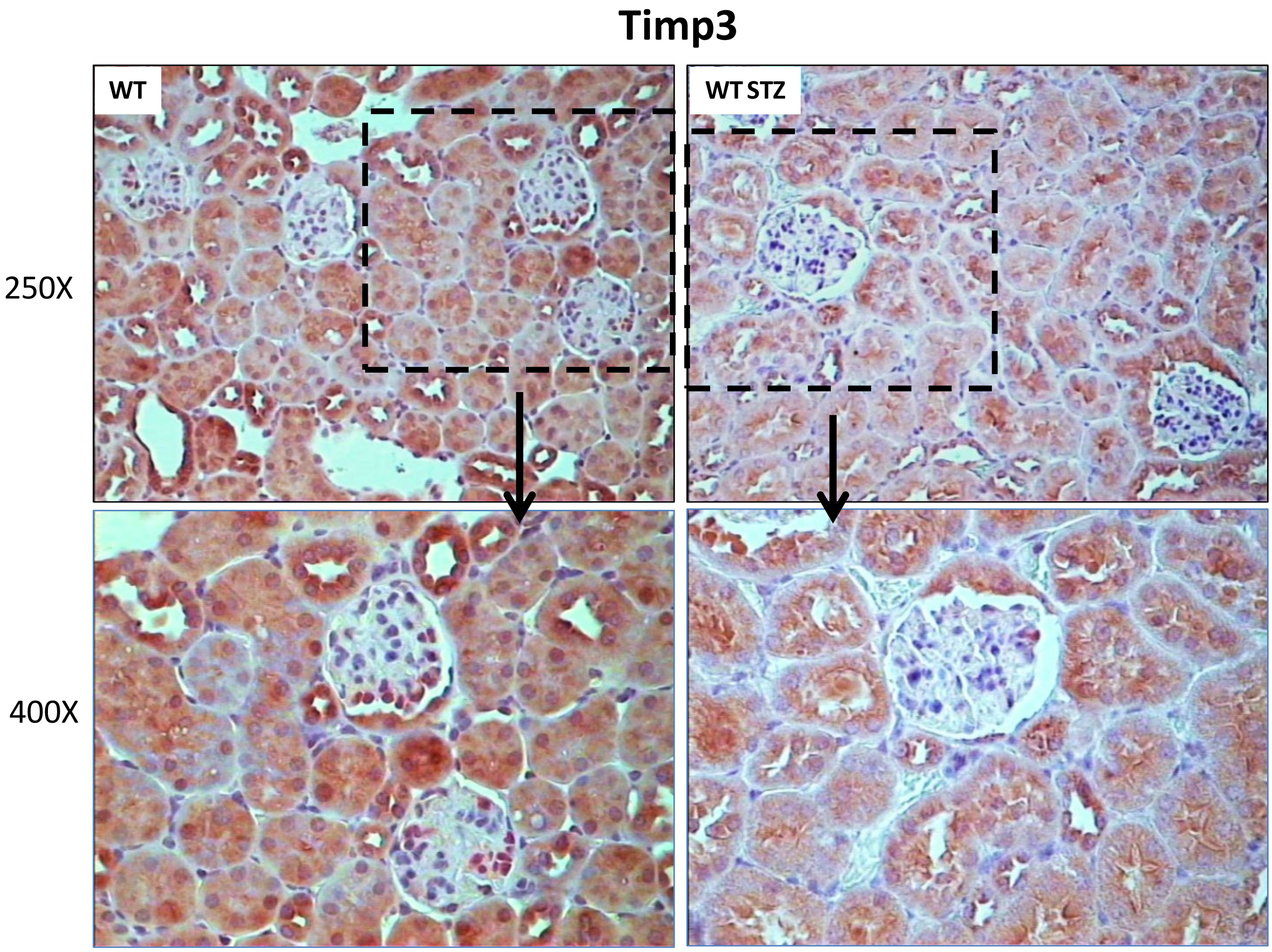

Supplemental Figure 1: TIMP3 expression in kidney of normoglycemic and diabetic WT mice. Immunohistochemical staining of kidney sections from normoglycemic and diabetic WT mice showing a decrease in TIMP3 expression in diabetic conditions. Magnification 250x (top panels) and 400x (bottom panels).

Supplemental Figure 2

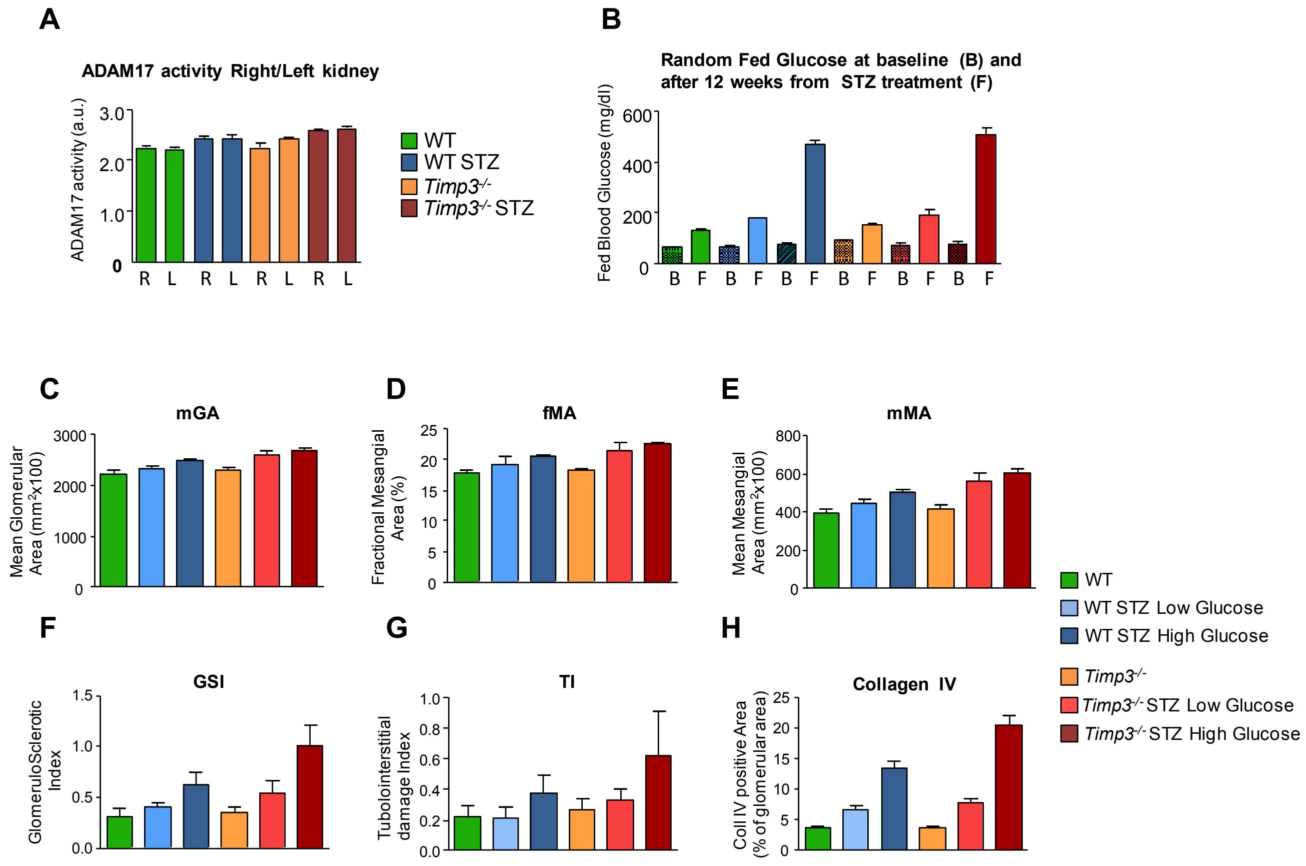

Supplemental Figure 2: Renal features of WT and *Timp3*$^{-/-}$ mice after STZ treatment. (**A**) Fluorimetric measurement of ADAM17 proteolytic activity in right and left kidneys of healthy and diabetic WT and *Timp3*$^{-/-}$ mice (n=6). (**B**) Glycemic values of WT and *Timp3*$^{-/-}$ mice (either resistant, STZ LG, and sensible, STZ HG, to STZ treatment) compared to untreated control (n=6) at baseline (B) and after 12 weeks from STZ treatment. (**C-G**) Assessment of kidney damage in the same groups of mice described in B, estimated by mean glomerular area (mGA) (C), fractional mesangial areas (fMA) (D), mean mesangial areas (mMA) (E), glomerusclerosis index (GSI) (F), tubulointerstial damage index (TI) (G) (n=6 for all). (**H**) Evaluation of collagen IV positive areas in kidney from the same groups of mice described in B (n=6).

Supplemental Figure 3

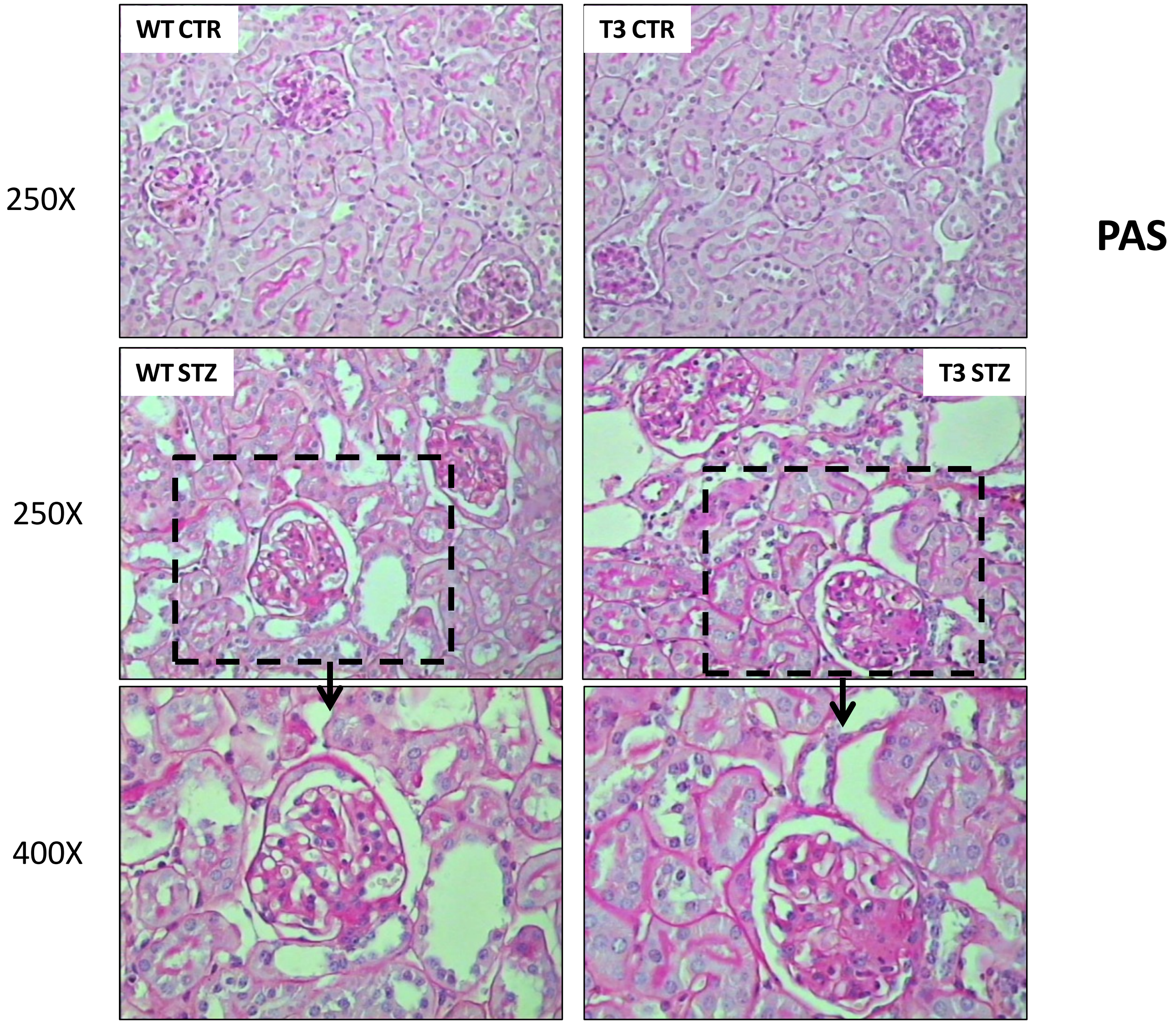

Supplemental Figure 3. PAS staining of kidney sections from normoglycemic and diabetic WT and *Timp3*$^{-/-}$ mice.

Supplemental Figure 4

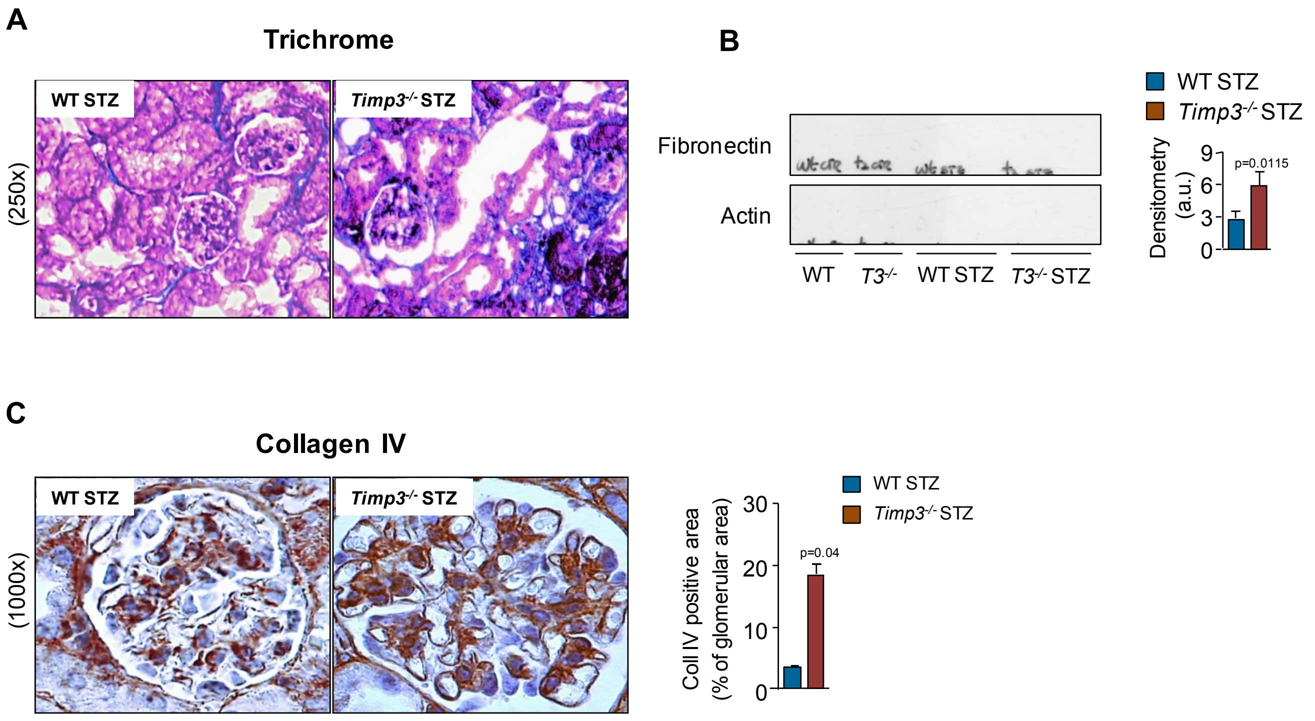

Supplemental Figure 4: Histological features of WT and *Timp3*$^{-/-}$ diabetic kidney. (**A** and **C**) Trichrome (A) and collagen IV (C) staining of kidney sections from diabetic WT and *Timp3*$^{-/-}$ mice. Picture magnification is shown on the left. Quantification of collagen IV positive area is shown (n=6, Student's t test). (**B**) Representative western blot of lysates from healthy and diabetic WT and *Timp3*$^{-/-}$ kidneys showing increased fibronectin expression in STZ-treated *Timp3*$^{-/}$ mice. Actin was used as a loading control. Densitometric analysis of results is shown on the right (n=6, Student's t test).

Supplemental Figure 5

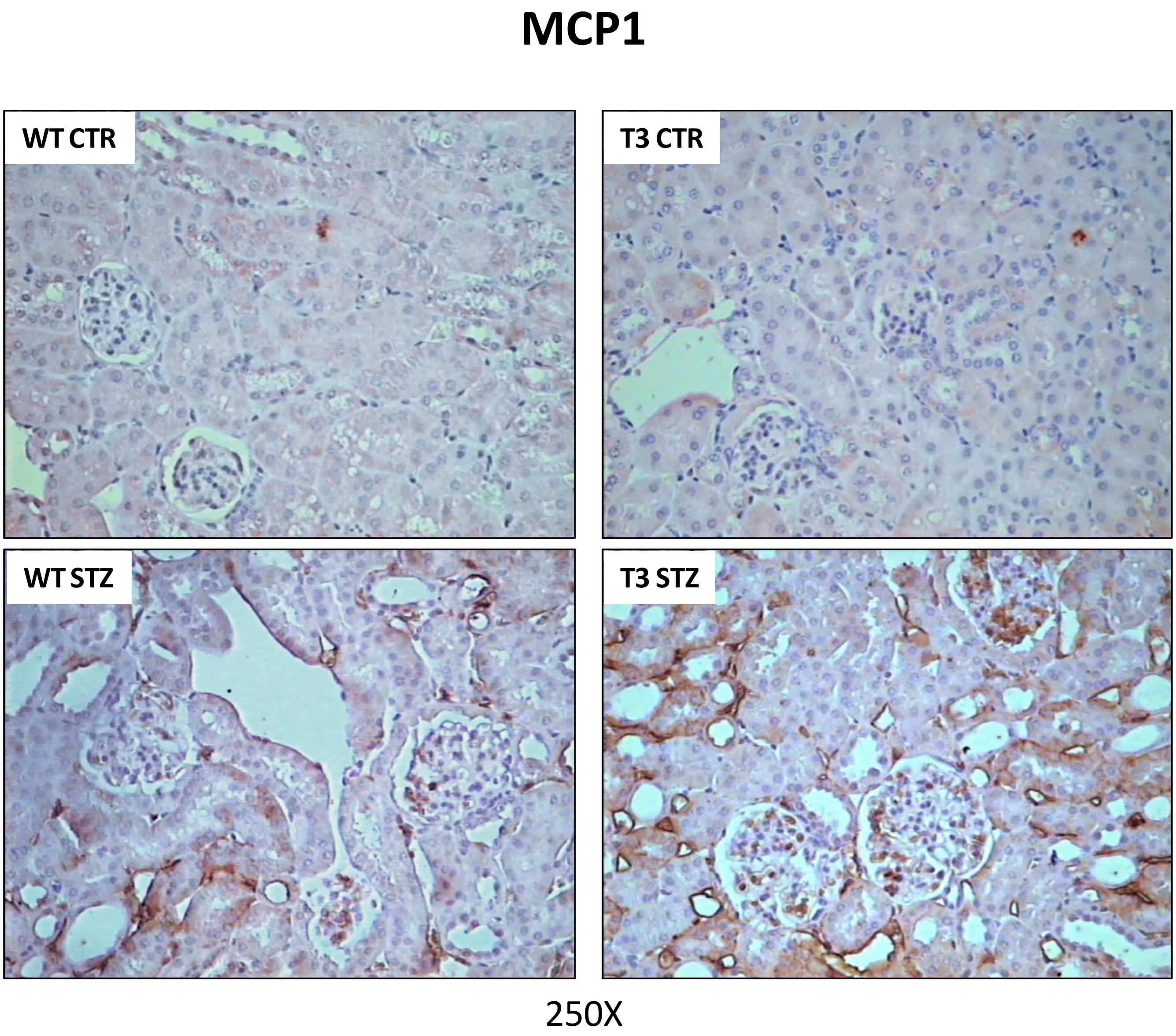

Supplemental Figure 5: MCP-1 expression in kidney of normoglycemic and diabetic WT and *Timp3*$^{-/-}$ mice. Immunohistochemical staining of kidney sections from normoglycemic and diabetic WT and *Timp3*$^{-/-}$ mice showing increased MCP-1 expression in diabetic conditions. Magnification 250x.

Supplemental Figure 6

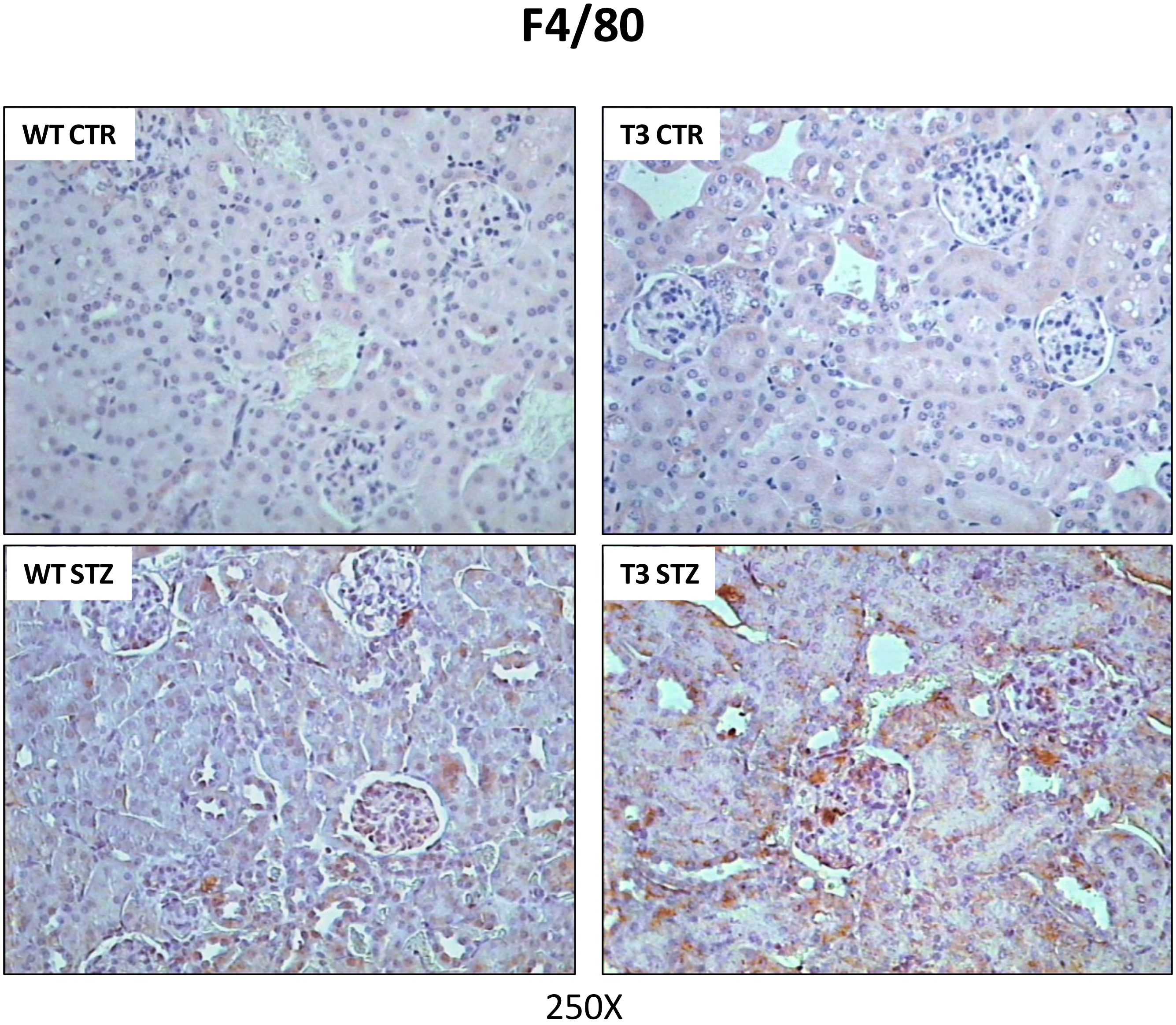

Supplemental Figure 6: F4/80 expression in kidney of normoglycemic and diabetic WT and *Timp3*$^{-/-}$ mice. Immunohistochemical staining of kidney sections from normoglycemic and diabetic WT and *Timp3*$^{-/-}$ mice showing increased F4/80 expression in diabetic conditions. Magnification 250x.

Supplemental Figure 7

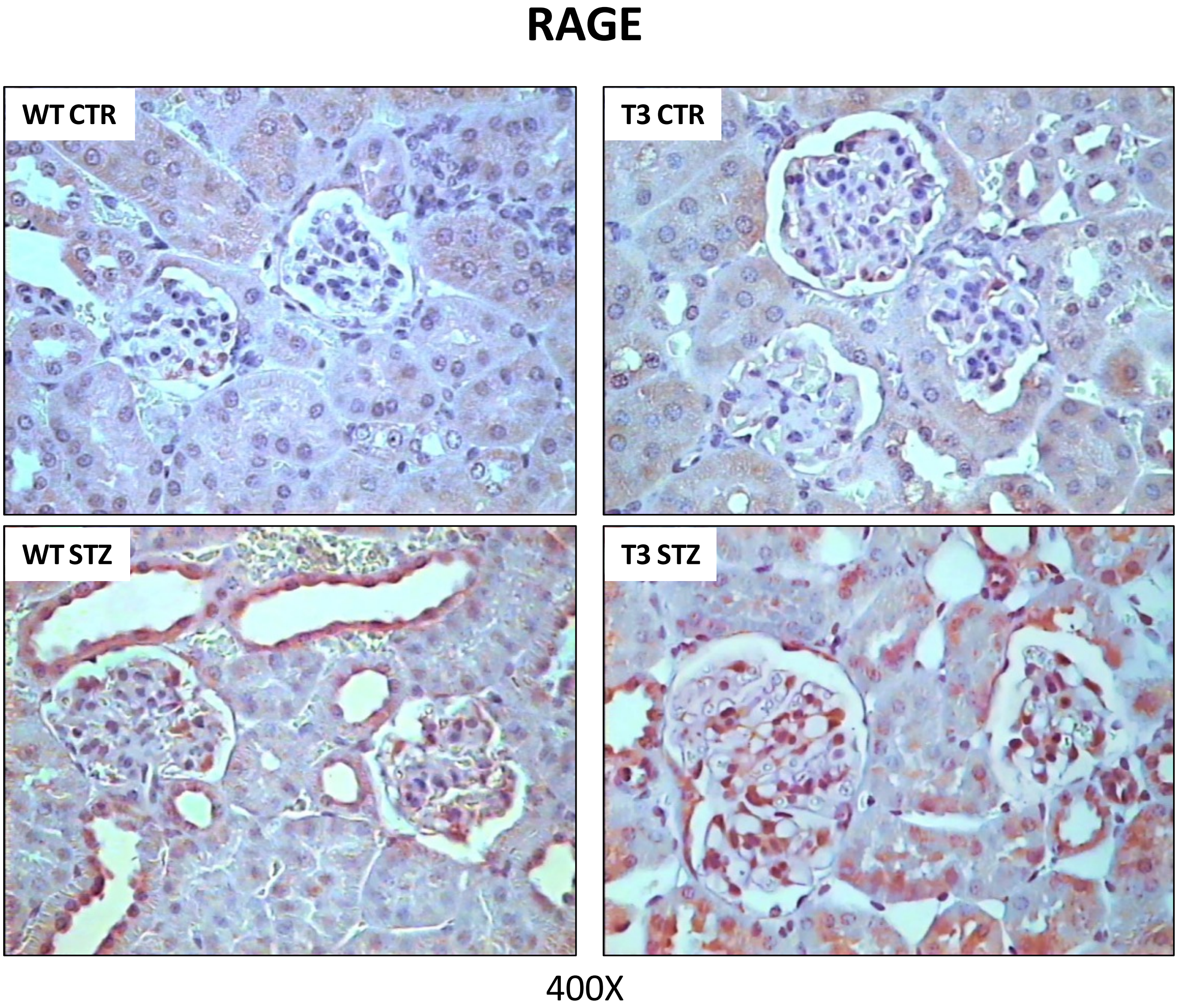

Supplemental Figure 7: RAGE expression in kidney of normoglycemic and diabetic WT and *Timp3*$^{-/-}$ mice. Immunohistochemical staining of kidney sections from normoglycemic and diabetic WT and *Timp3*$^{-/-}$ mice showing increased RAGE expression in diabetic conditions. Magnification 400x.

Supplemental Figure 8

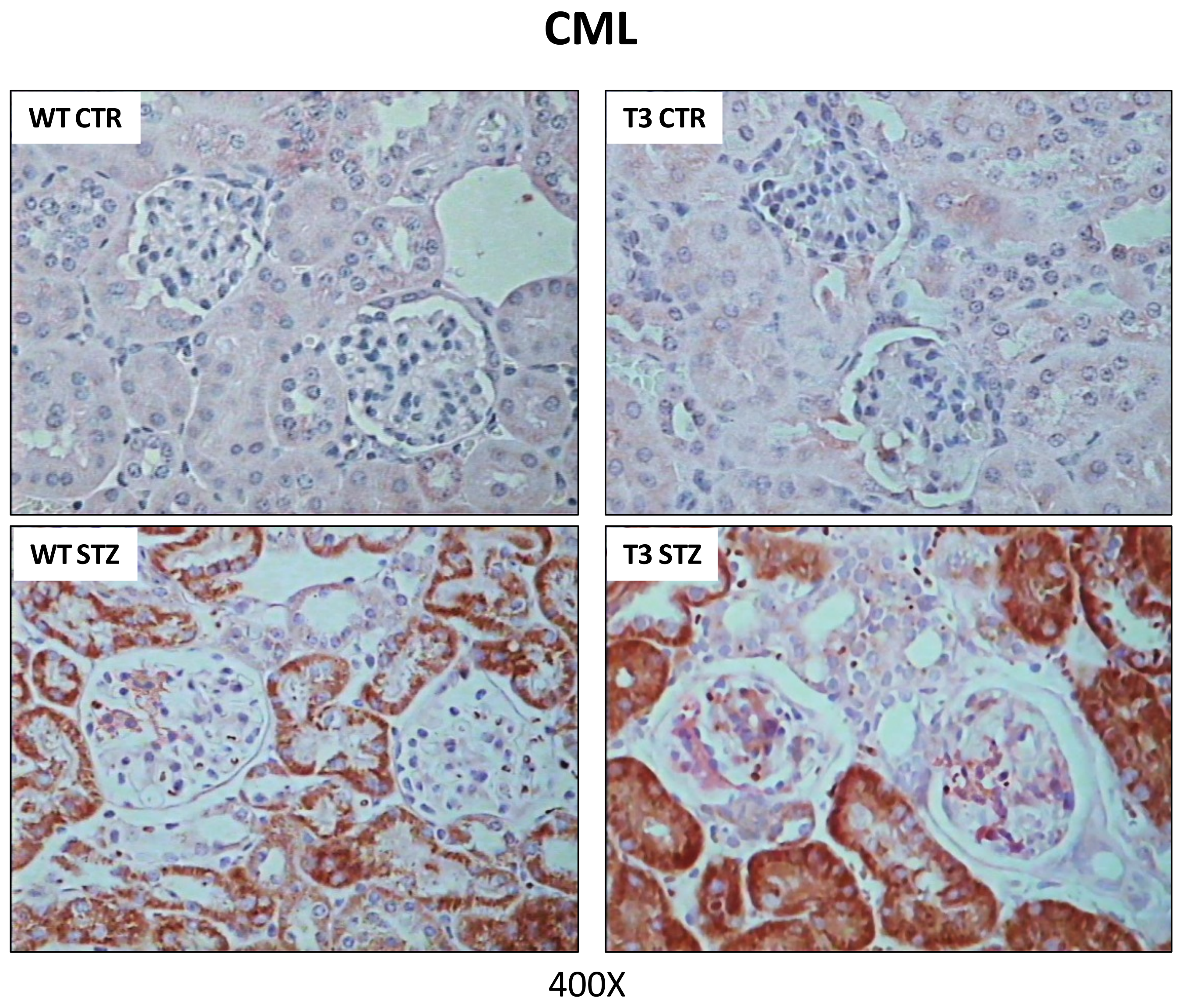

Supplemental Figure 8: N-Carboxymethyl-lysine (CML) expression in kidney of normoglycemic and diabetic WT and *Timp3*$^{-/-}$ mice. Immunohistochemical staining of kidney sections from normoglycemic and diabetic WT and *Timp3*$^{-/-}$ mice showing increased N-Carboxymethyl-lysine expression in diabetic conditions. Magnification 400x.

Supplemental Figure 9

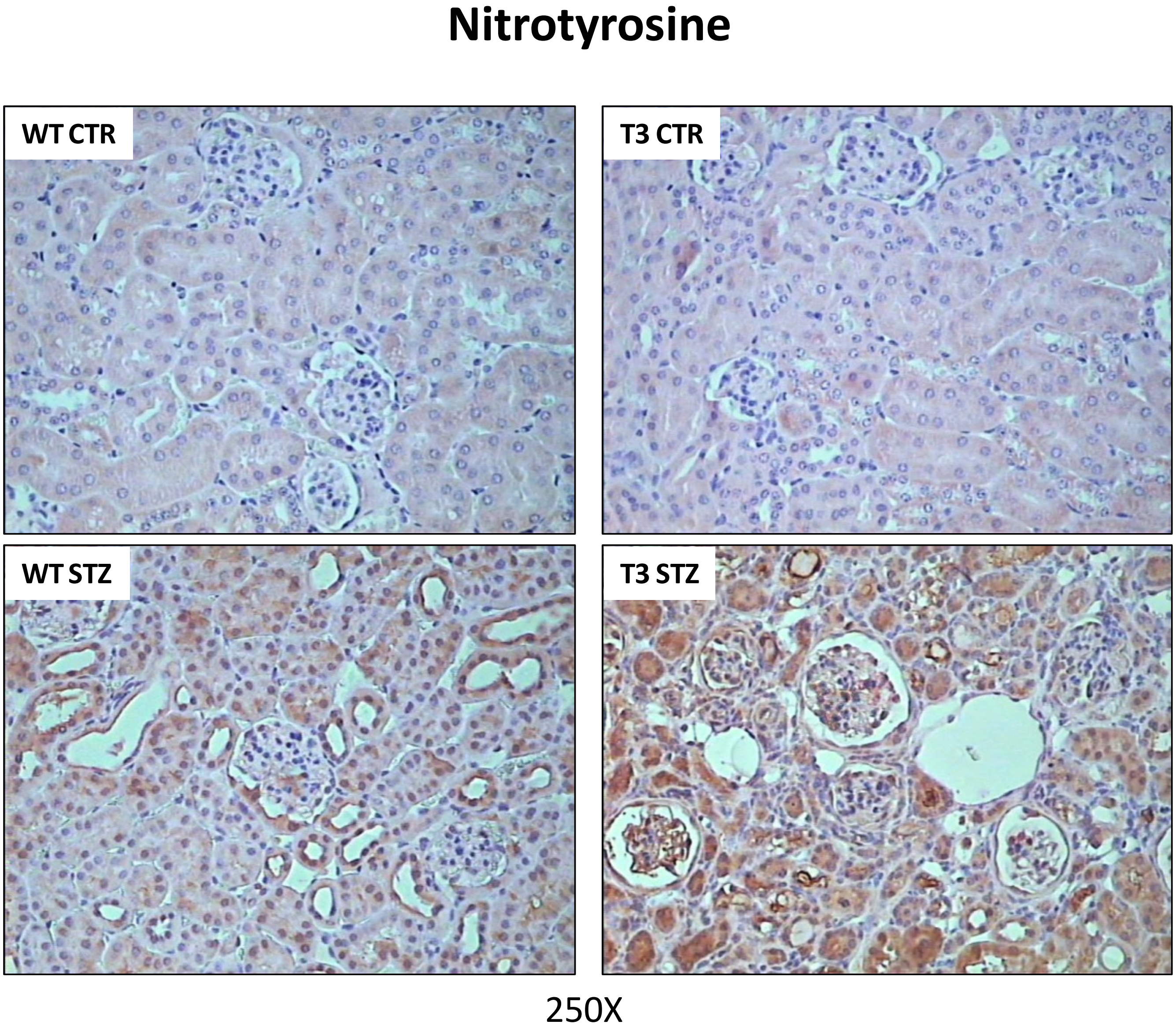

Supplemental Figure 9: Nitrotyrosine expression in kidney of normoglycemic and diabetic WT and *Timp3*$^{-/-}$ mice. Immunohistochemical staining of kidney sections from normoglycemic and diabetic WT and *Timp3*$^{-/-}$ mice showing increased Nitrotyrosine expression in diabetic conditions. Magnification 250x.

Supplemental Figure 10

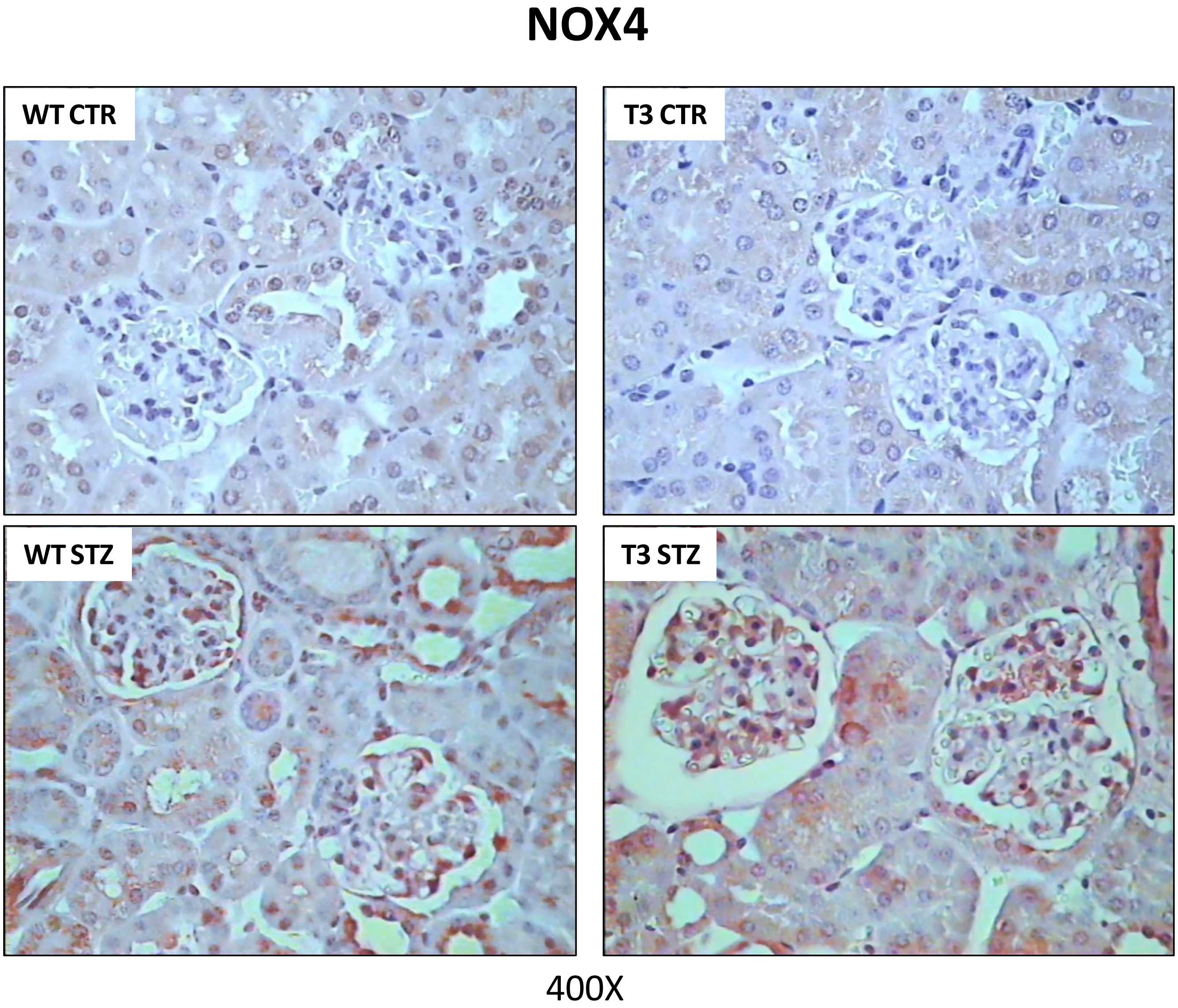

Supplemental Figure 10: NOX4 expression in kidney of normoglycemic and diabetic WT and *Timp3*$^{-/-}$ mice. Immunohistochemical staining of kidney sections from normoglycemic and diabetic WT and *Timp3*$^{-/-}$ mice showing increased NOX4 expression in diabetic conditions. Magnification 400x.

Supplemental Figure 11

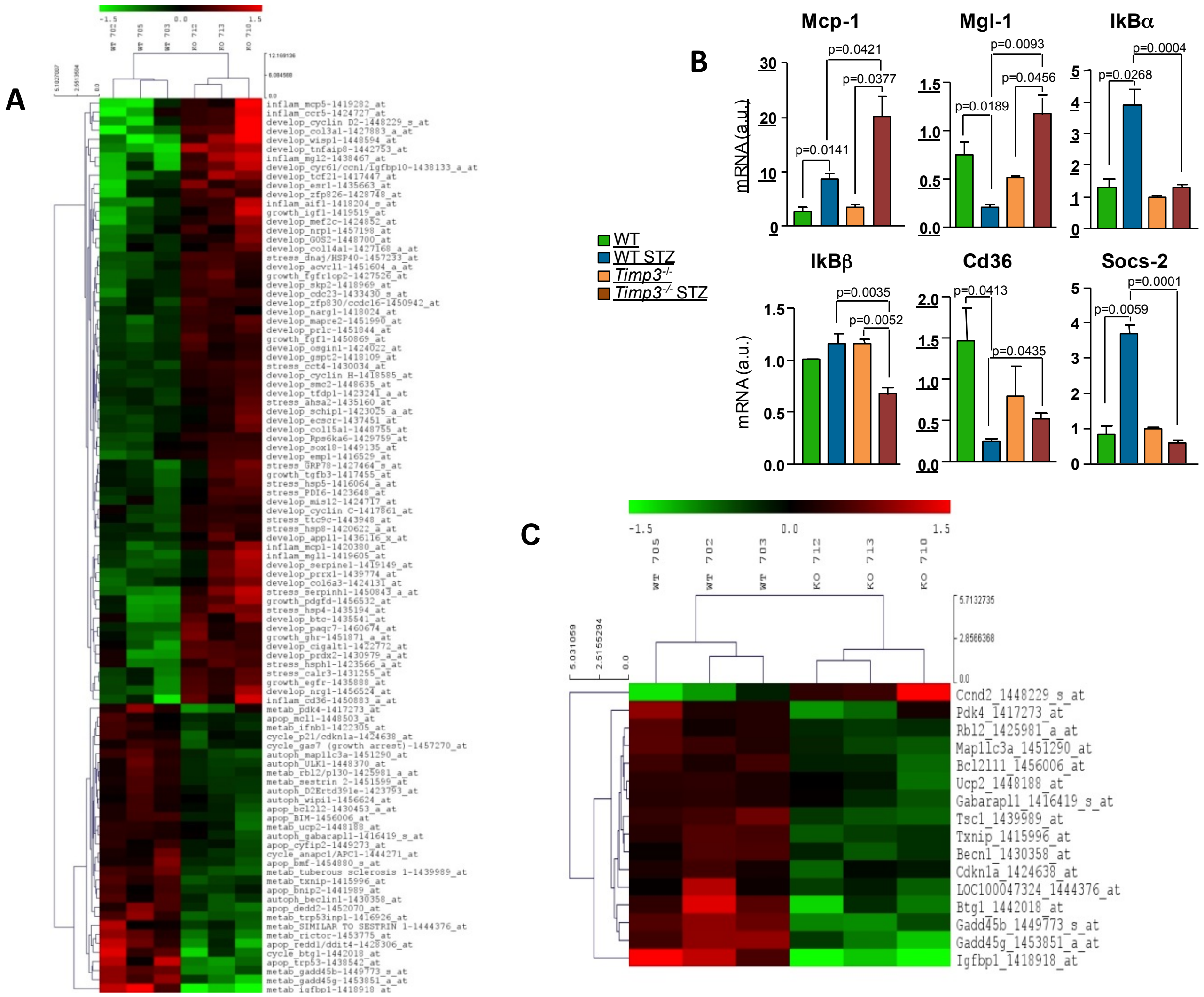

Supplemental Figure 11: Expression profiling of kidneys from diabetic WT and *Timp3$^{-/-}$* mice. (**A**) Results are shown using a heat map visualization and hierarchical clustering in which columns represent the different kidney mouse samples analyzed and rows represent the various genes, indicated on the right with their identifier including: gene functional category, gene symbol and Affymetrix probe ID. Genes expressed at low levels are in green; those expressed at high levels are in red. Expression values are in Log2 scale and mean-centered. Each group was composed of 3 mice. (**B**) Real-time PCR on kidney mRNA from healthy and diabetic WT and *Timp3$^{-/-}$* mice showing upregulation of inflammatory genes and dowregulation of anti-inflammatory ones (n=6, Student's t test). (**C**) Heat map and hierarchical clustering of FoxO1 target genes in diabetic WT and *Timp3$^{-/-}$* kidney (n=3). Results are shown as in A.

Supplemental Figure 12

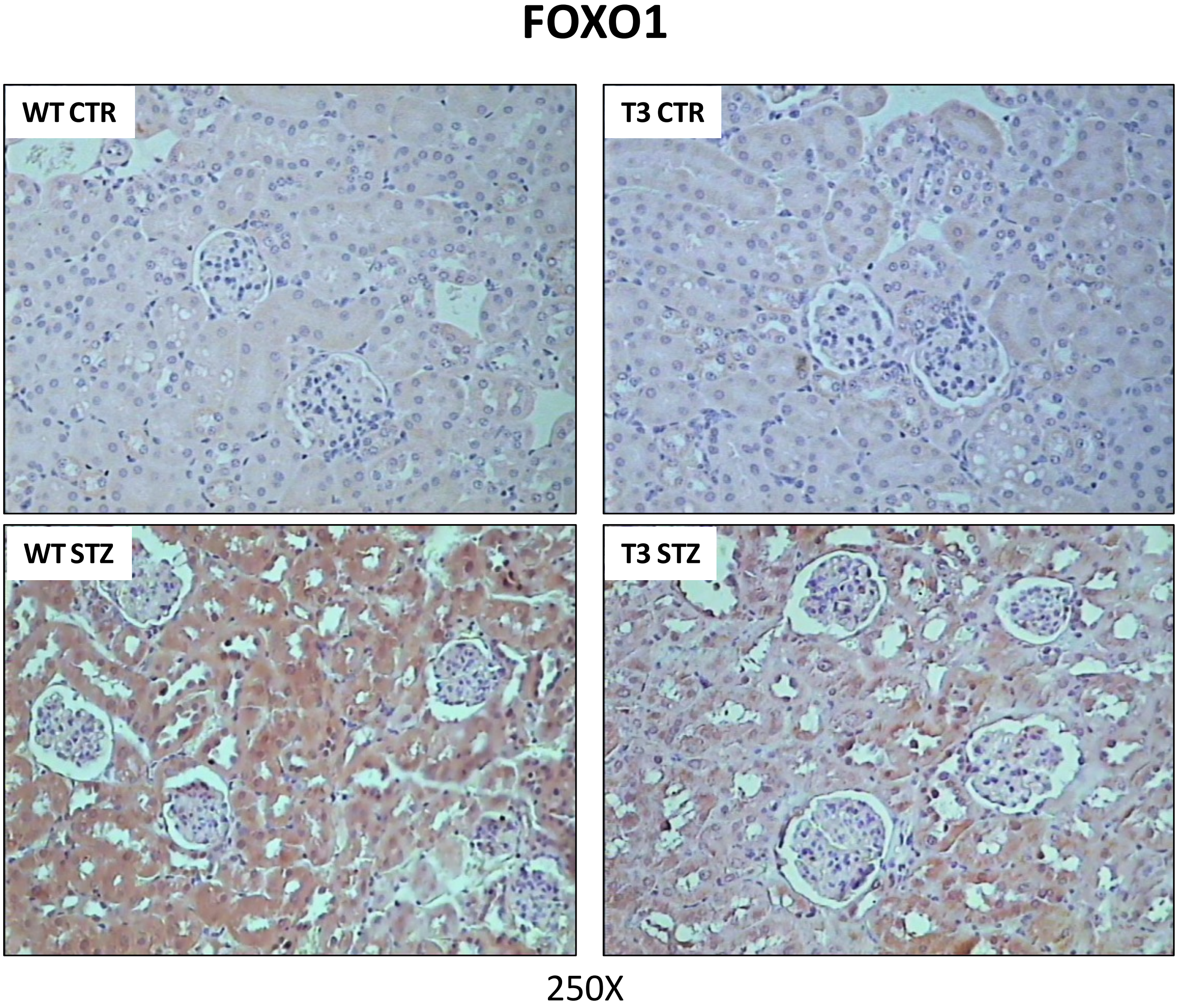

Supplemental Figure 12: FOXO1 expression in kidney of normoglycemic and diabetic WT and *Timp3*$^{-/-}$ mice. Immunohistochemical staining of kidney sections from normoglycemic and diabetic WT and *Timp3*$^{-/-}$ mice showing decreased FOXO1 expression in diabetic *Timp3*$^{-/-}$ mice compared to diabetic WT. Magnification 250x.

Supplemental Figure 13

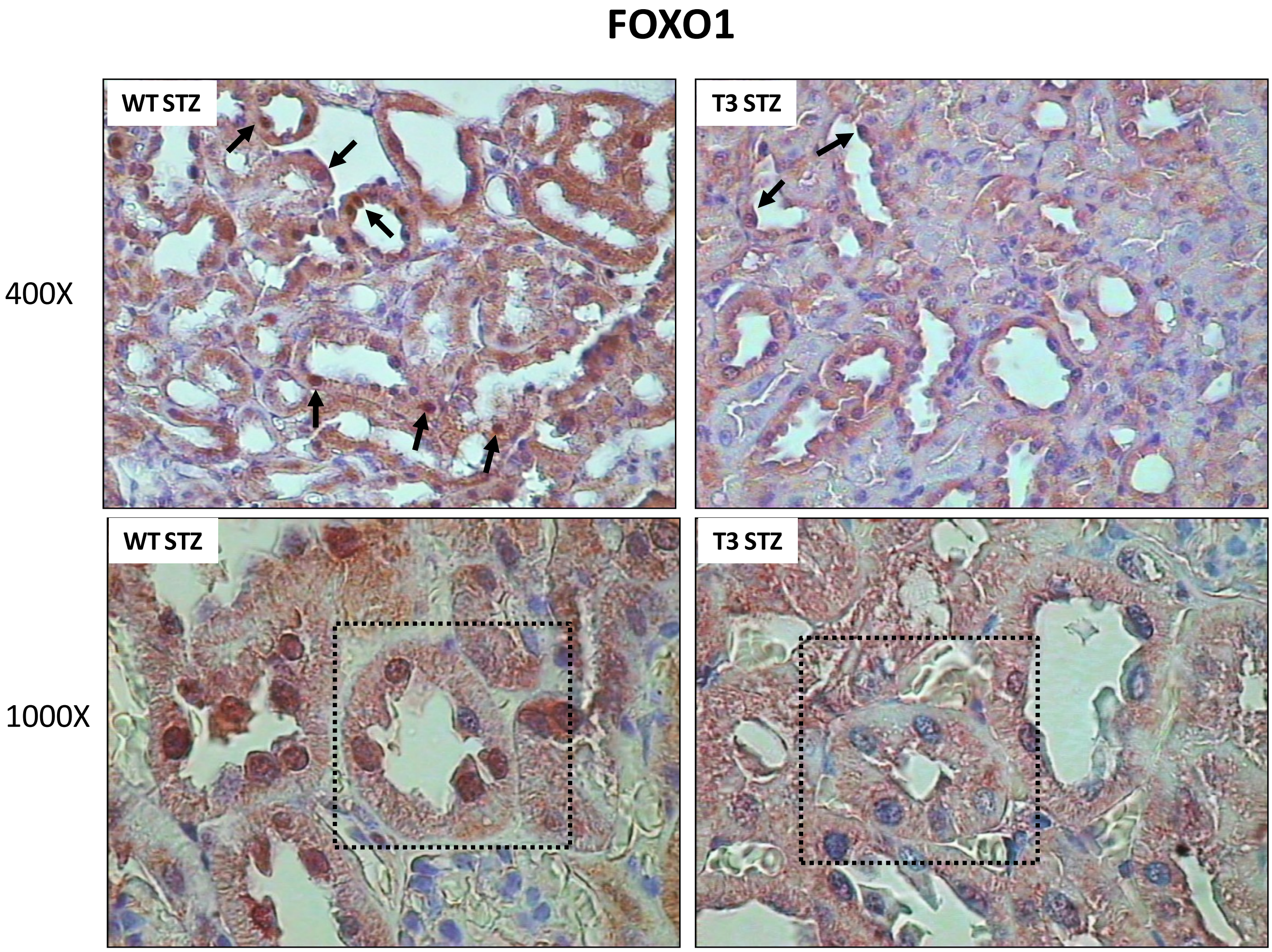

Supplemental Figure 13: FOXO1 expression in the tubular compartment of diabetic WT and *Timp3*$^{-/-}$ mice. Immunohistochemical staining of the tubular compartment of kidney from healthy and diabetic WT and *Timp3*$^{-/-}$ mice clearly illustrating nuclear accumulation of FOXO1 in diabetic WT mice and nuclear exclusion of the protein in diabetic *Timp3*$^{-/-}$ mice (top panels 400x, bottom panels 1000x). Arrows point to nuclei, dashed squares refer to Figure 3.

Supplemental Figure 14

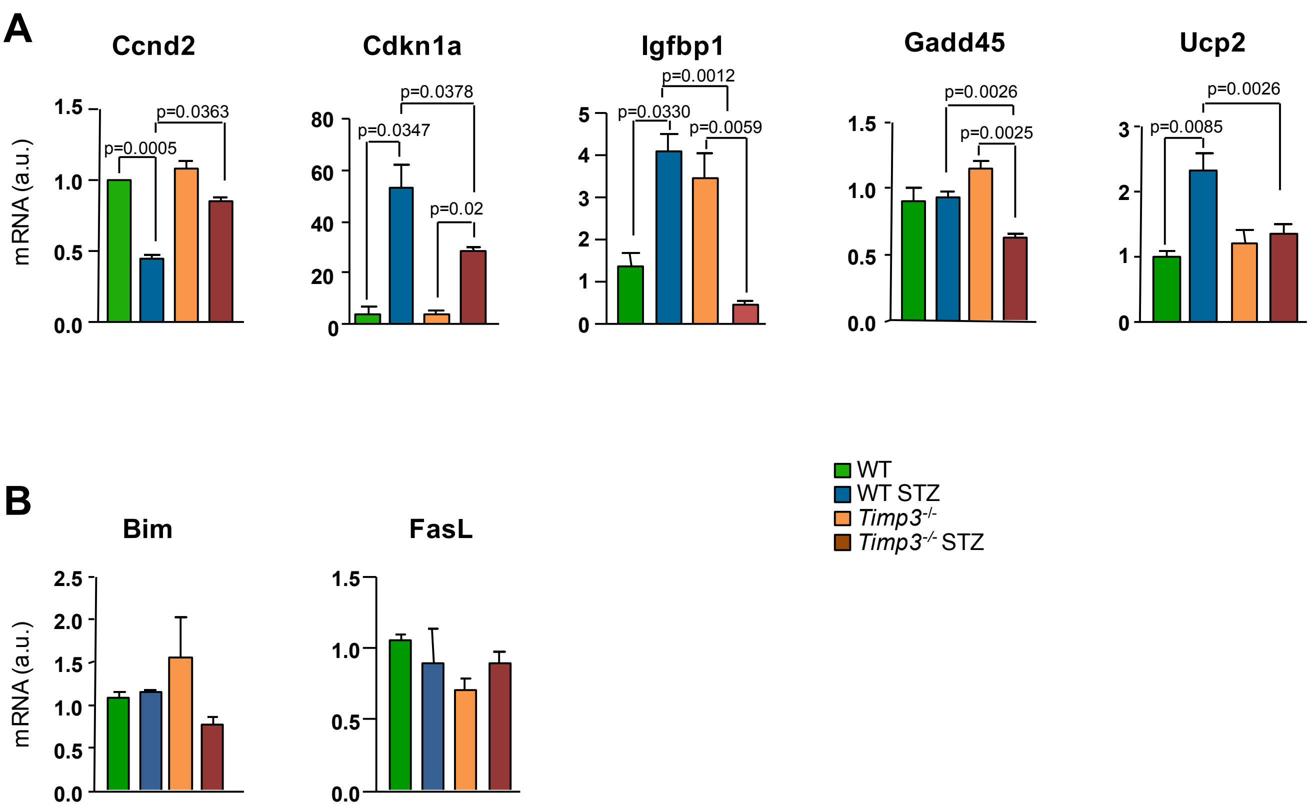

Supplemental Figure 14: Modulation of FOXO target genes. (**A-B**) Quantitative PCR analysis of Foxo1 target genes in healthy and diabetic WT and *Timp3*$^{-/-}$ kidneys, showing regulation (A) or unchanged levels of expression (B). (n=6, Student's t test).

Supplemental Figure 15

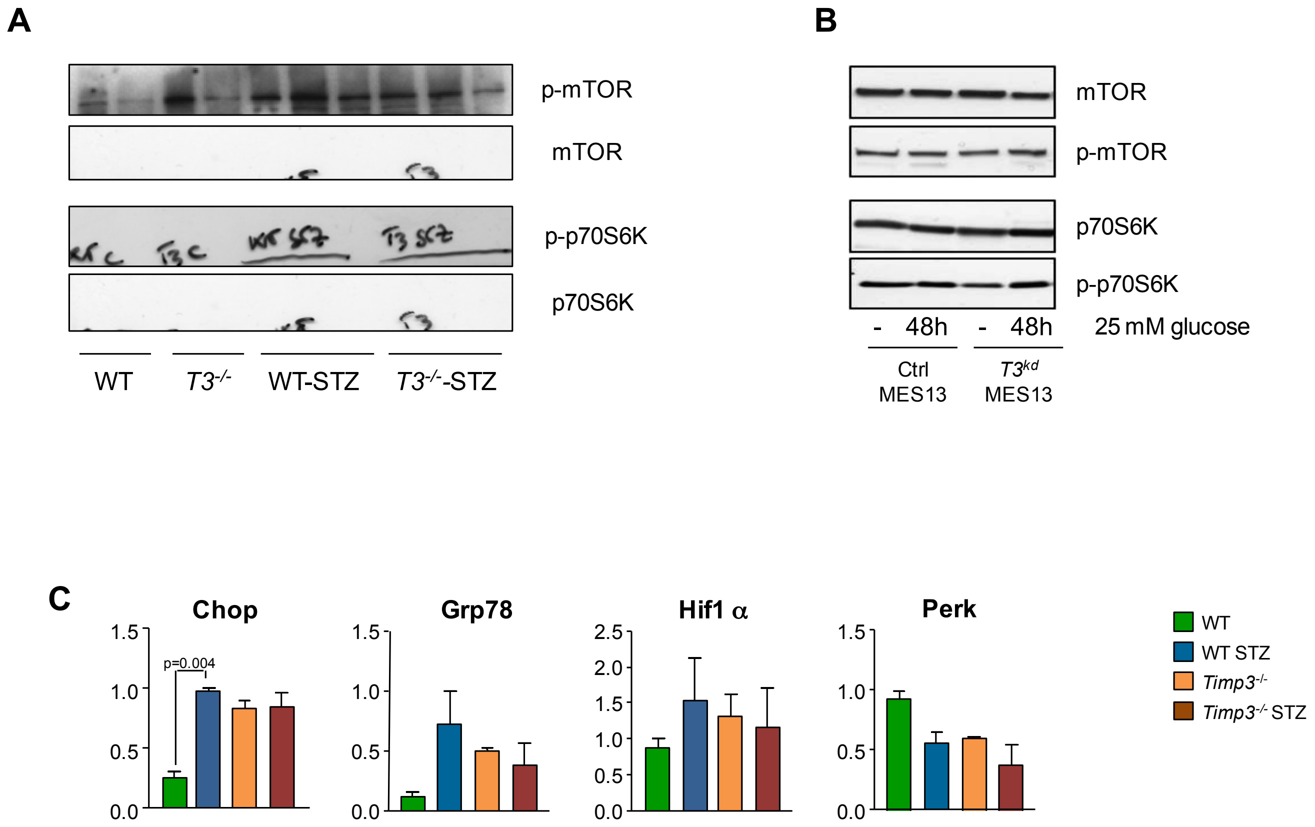

Supplemental Figure 15: Signal transduction pathways unchanged by TIMP3 deficiency. (**A-B**) Representative western blot analysis of diabetic WT and *Timp3*$^{-/-}$ kidney (**A**) and *T3*^{kd} MES13 cell lysates (**B**) showing no differences in mTOR and p70S6 kinase expression and phosphorylation. (**C**) Real time PCR analysis of ER stress-related genes in normoglycemic and diabetic WT and *Timp3*$^{-/-}$ kidneys (n=6, Student's t test).

Supplemental Figure 16

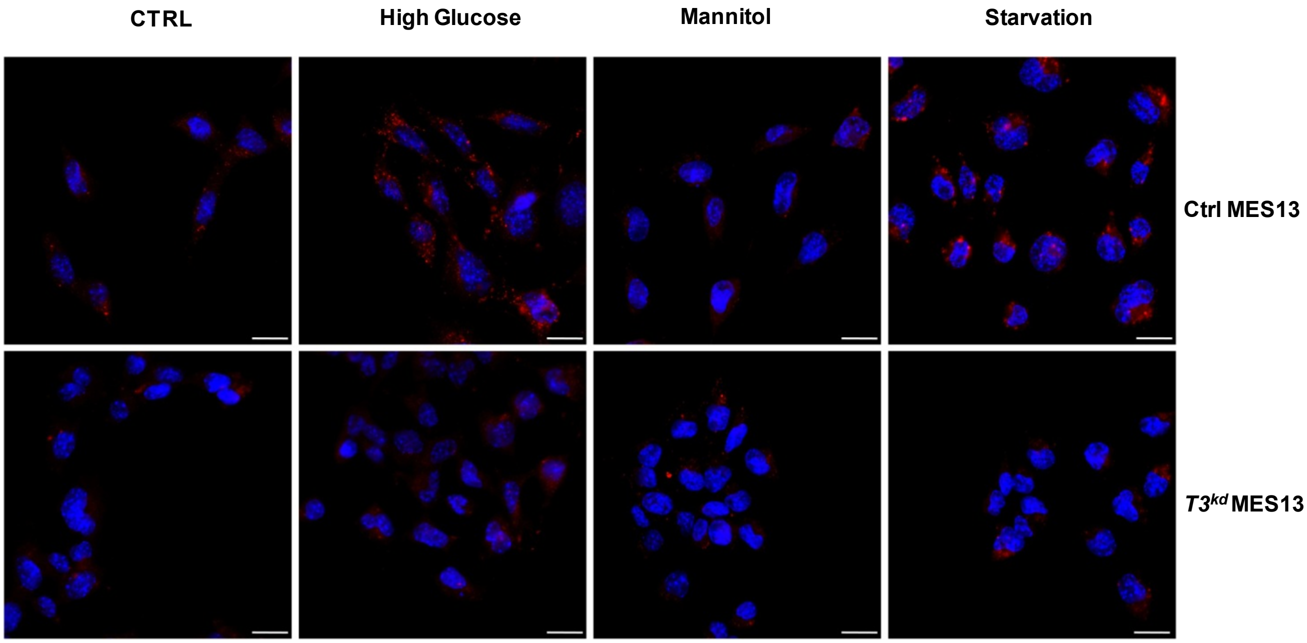

Supplemental Figure 16: Immunofluorescence for LC3A/B in *T3*^{kd} and control MES13 cells. Digital zoom of figure 4E. Immunofluorescence (IF) for LC3A/B (red) in *T3*^{kd} and control MES13 cells left untreated or treated with high glucose, mannitol, or serum-starved for 24 hrs. Cells were counterstained with DAPI to detect nuclei (blue). Magnification: 60x. Scale Bars: 20μm.

Supplemental Figure 17

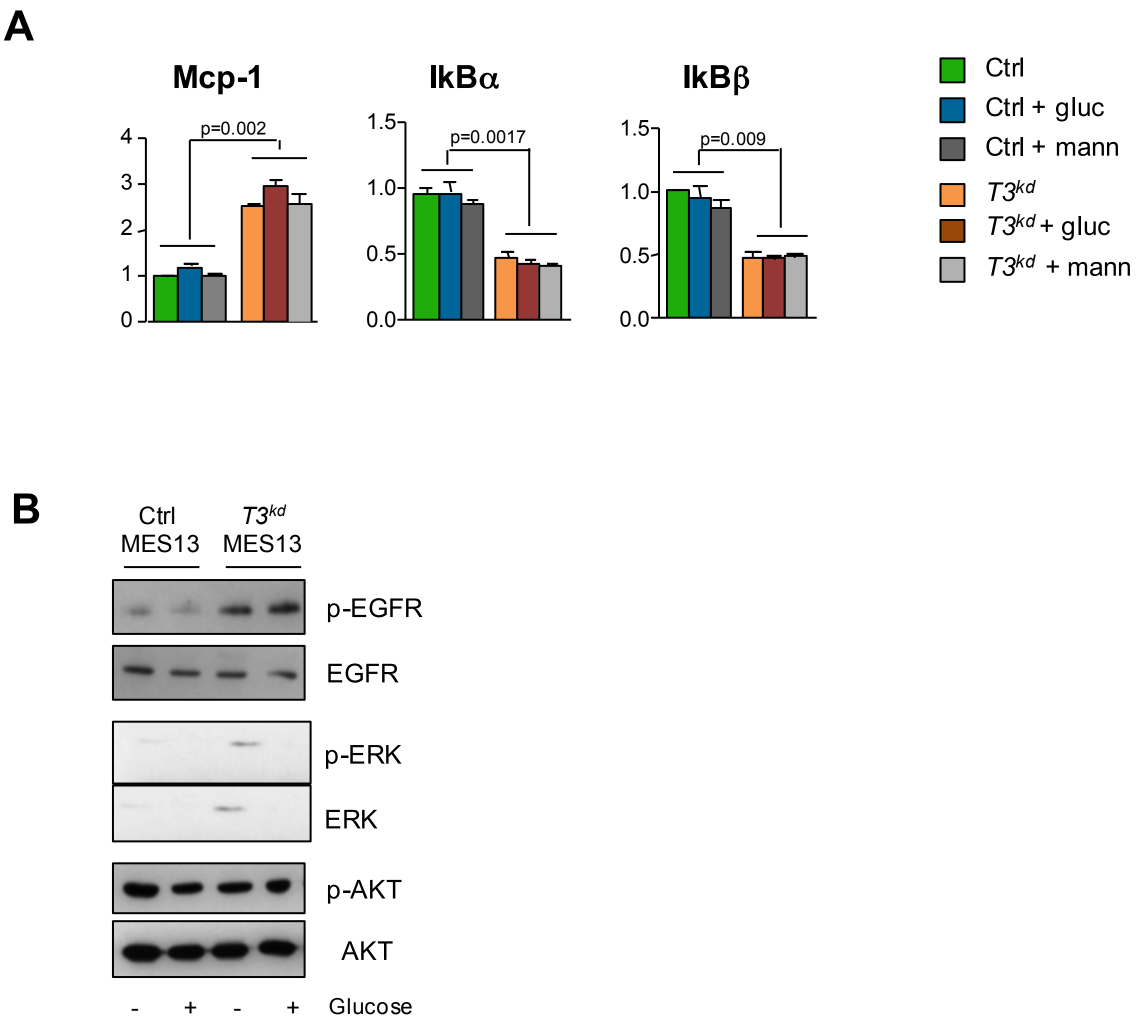

Supplemental Figure 17: Characterization of *T3*^{kd} MES13 cells. (**A**) Real-time PCR on *T3*^{kd} or control MES13 cells grown in basal medium (low glucose) or treated with high glucose or mannitol (25mM) showing modulation of inflammatory genes (n=3, Student's t test). (**B**) Representative western blot analysis of lysates from *T3*^{kd} or control MES13 cells left untreated or treated as in (A). Levels of phosphorylation of Akt (Ser473), ERK (Thr202/Tyr204) and EGFR (Tyr1068) were assayed.

Supplemental Figure 18

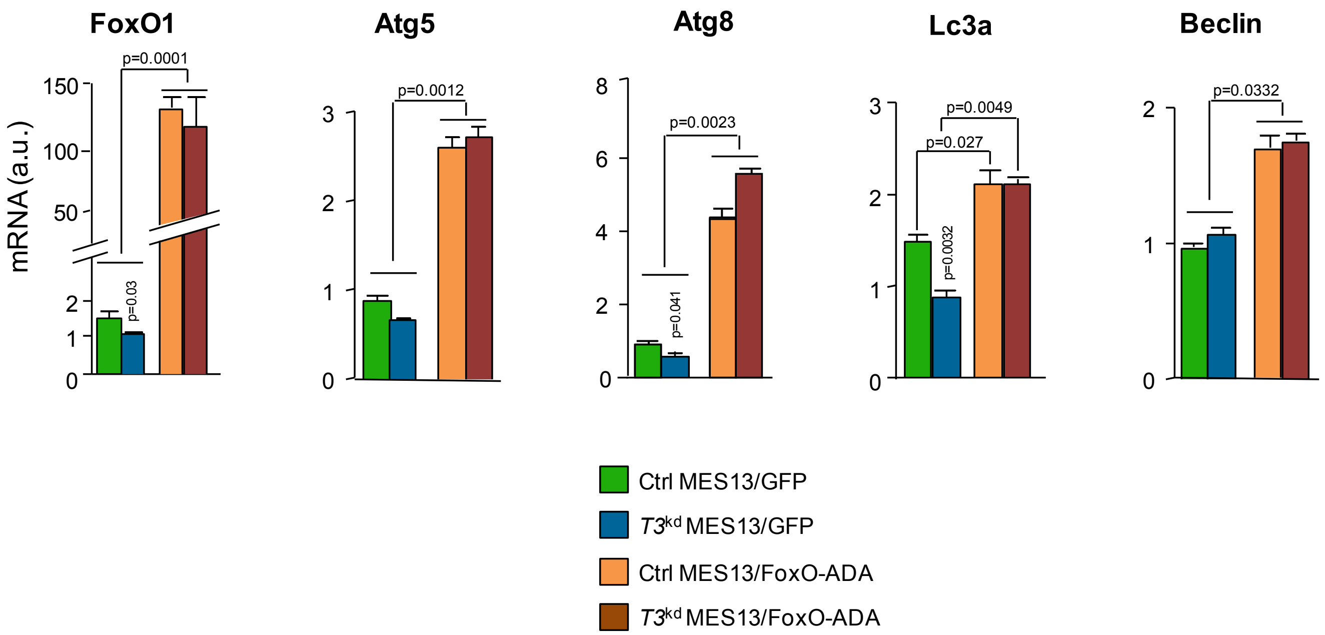

Supplemental Figure 18: Rescue of FoxO1 and its targets in *T3^{kd}* MES13 cells infected with FoxO1-ADA adenovirus. *T3^{kd}* and control MES13 cells were infected with FoxO1-ADA or GFP adenovirus and subjected to real time PCR to quantify the expression of FoxO1 and its autophagy-related target genes *Atg5*, *Atg8*, *Lc3* and *Beclin* (n=3, Student's t test).

Supplemental Figure 19

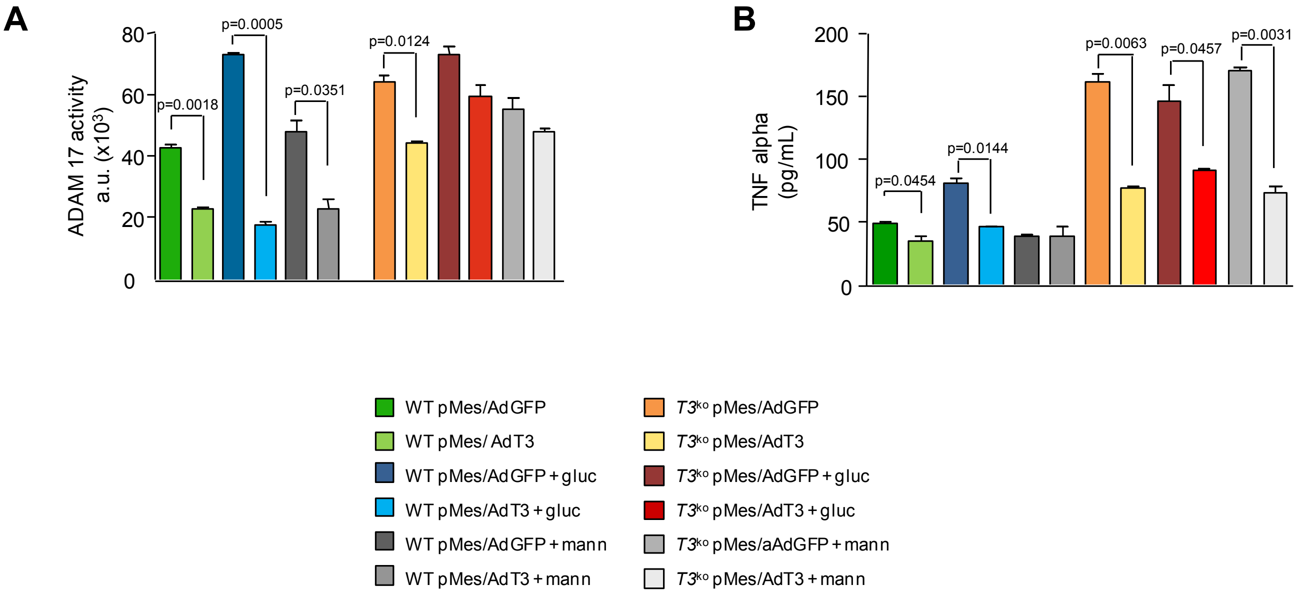

Supplemental Figure 19: ADAM17 activity in *T3^{ko}* primary mesangial cells. (**A**) Fluorimetric measurement of ADAM17 proteolytic activity in *T3^{ko}* and WT primary cells infected with GFP or TIMP3 adenovirus. Cells were either left untreated or treated with 25mM glucose or mannitol (n=3, Student's t test). (B) Soluble form of TNFα was measured by ELISA on supernatants of *T3^{ko}* and WT primary cells infected and treated as in A (n=3, Student's t test).

Supplemental Figure 20

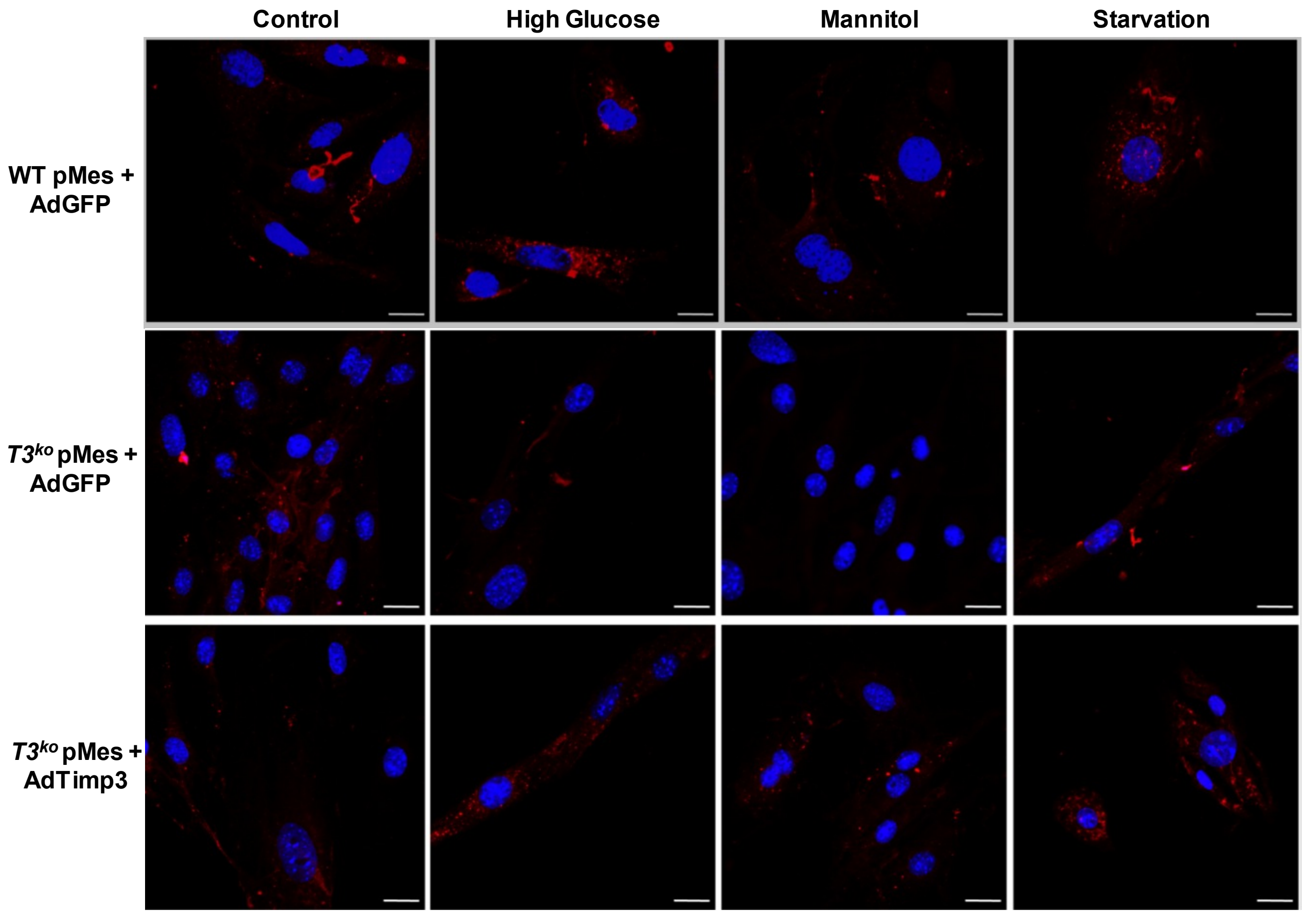

Supplemental Figure 20: Immunofluorescence analysis of LC3A/B in *T3*^{ko} and control pMes cells. Immunofluorescence (IF) for LC3A/B (red) in *T3*^{ko} and control pMes infected with GFP or TIMP3 adenovirus. Cells were left untreated or treated with high glucose, mannitol, or serum-starved for 24 hrs. Cells were counterstained with DAPI to detect nuclei (blue). Magnification: 60x. Scale Bars: 20μm. Insets for WT pMes + AdGFP cells (top panel) are 2x digital zoom of the original 60X magnification view.

Supplemental Figure 21

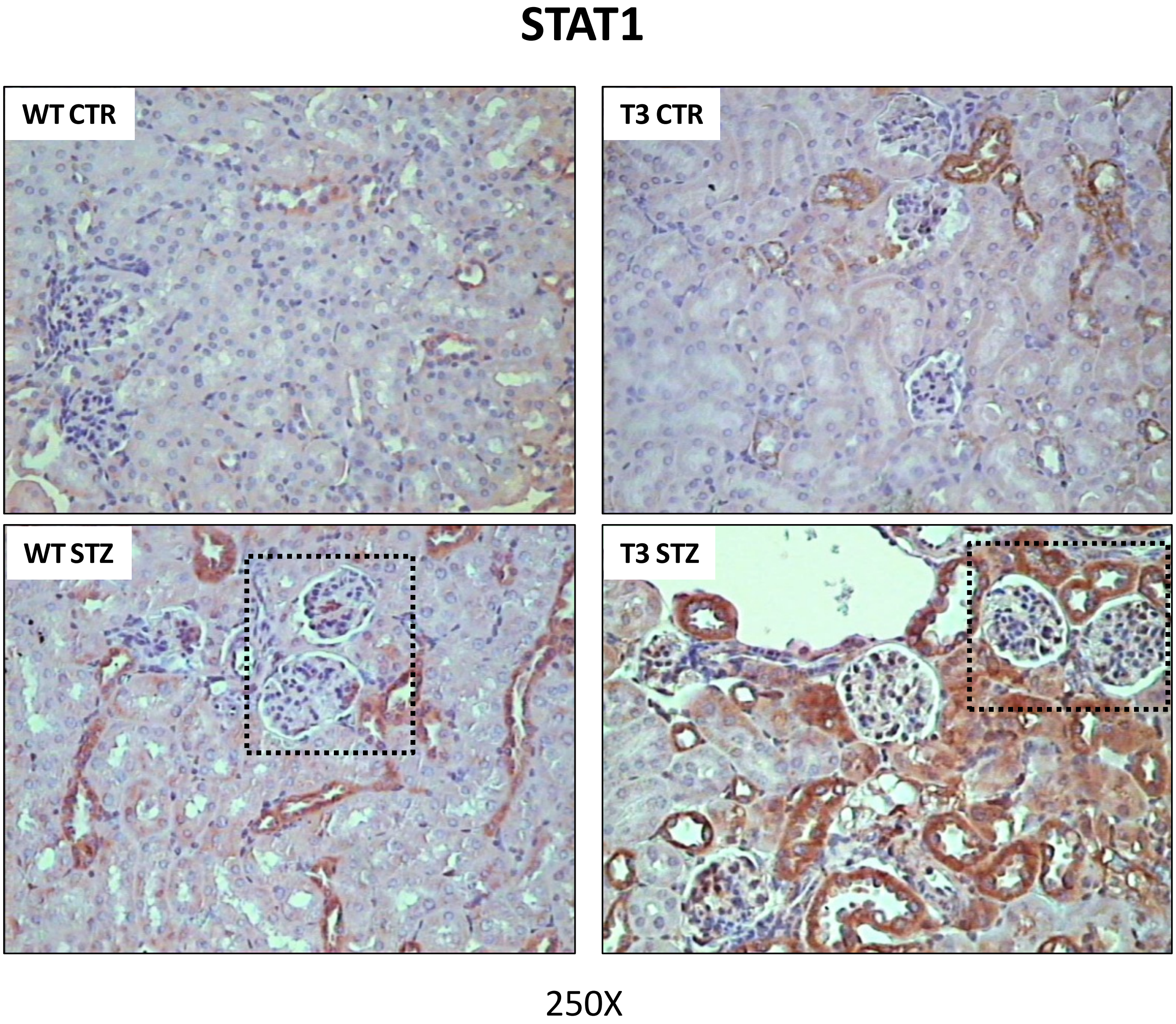

Supplemental Figure 21: STAT1 expression in kidney of normoglycemic and diabetic WT and *Timp3*$^{-/-}$ mice. Immunohistochemical staining of kidney sections from normoglycemic and diabetic WT and *Timp3*$^{-/-}$ mice showing increased STAT1 expression in diabetic *Timp3*$^{-/-}$ mice compared to diabetic WT. Magnification 250x.

Supplemental Figure 22

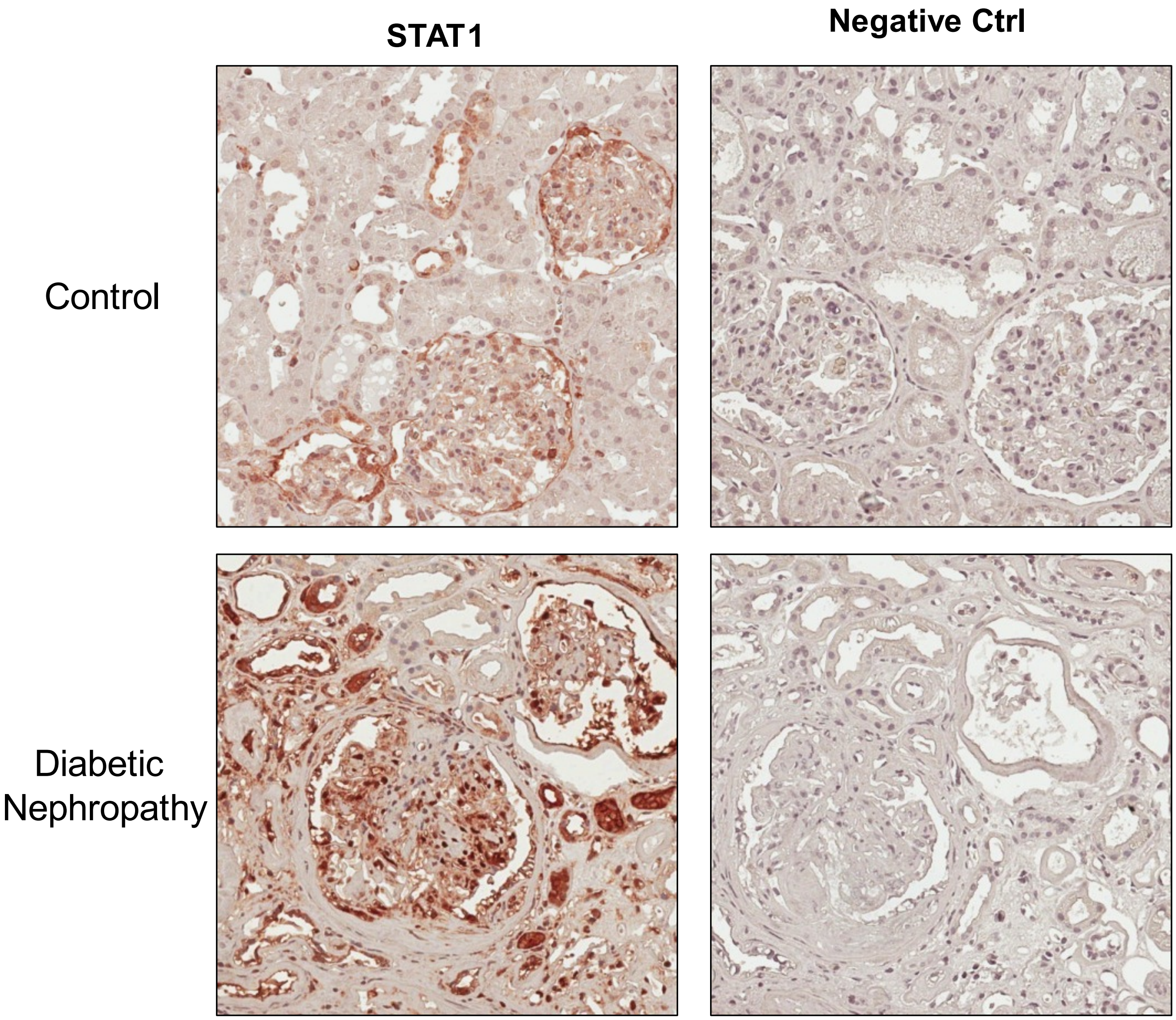

Supplemental Figure 22: STAT1 expression in kidney from healthy and diabetic subjects. STAT1 and control stainings of kidney sections from healthy and diabetic subjects. Scanning magnification 40x, zoom 20x.

Supplemental Figure 23

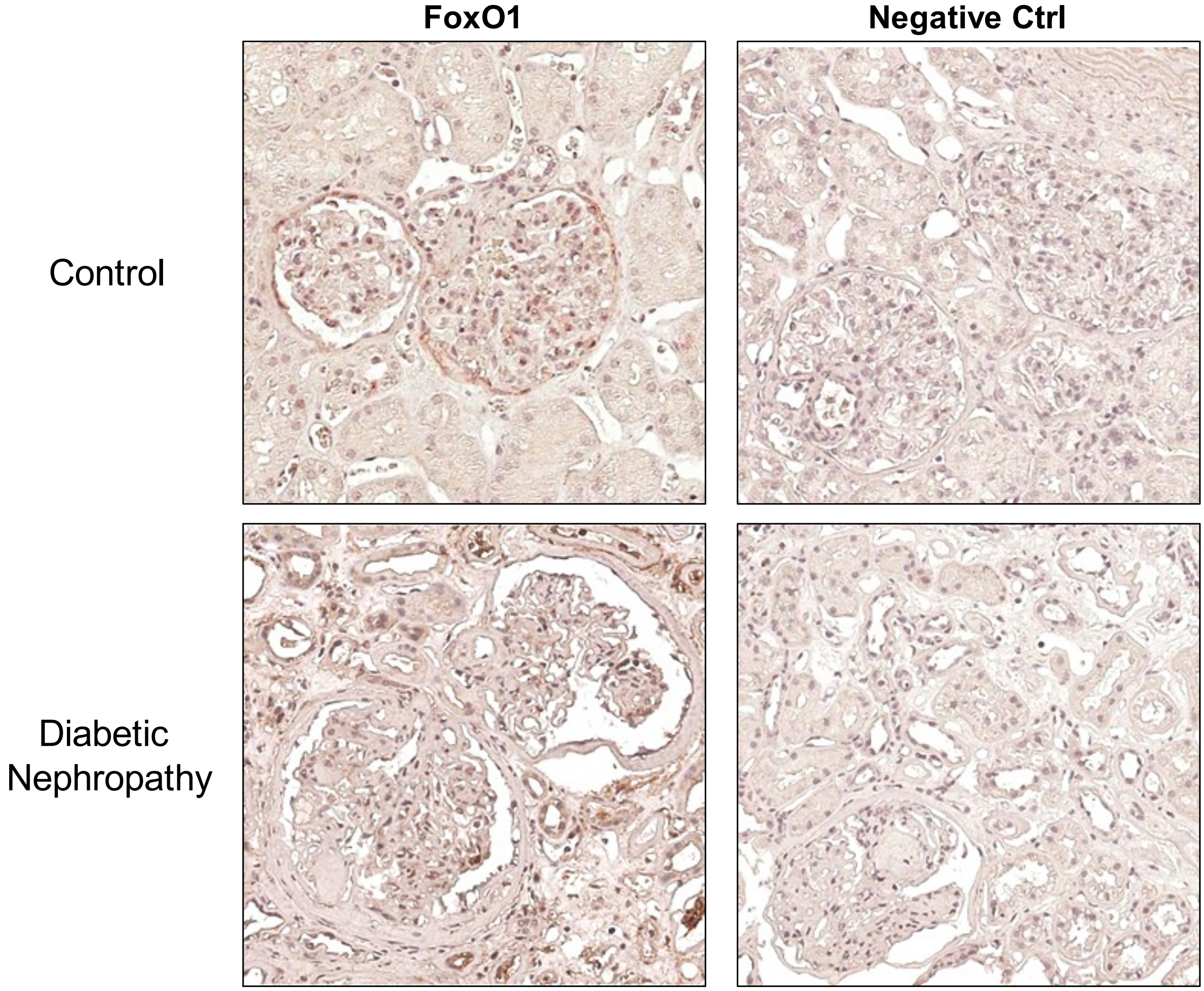

Supplemental Figure 23: FOXO1 expression in kidney from healthy and diabetic subjects. FOXO1 and control stainings of kidney sections from healthy and diabetic subjects. Scanning magnification 40x, zoom 20x.

Supplemental Table 1.

Genotype	Mean Weight (g) T0	Mean Glycemia (mg/dl) T0	Mean Weight (g) T12	Mean Glycemia (mg/dl)T12
**WT**	26 ± 3.4	95.8 ± 13.4	21.8 ± 1.9	492.2 ± 77
Timp3 $^{-/-}$	21.2 ± 1.8	154 ± 23.8	19.6 ± 1.9	488.4 ± 81.8

Supplemental Table 1: Metabolic parameters of WT and *Timp3* $^{-/-}$ **mice used in the study.** The table shows the mean ± SD of weight and glycemic values of mice at the beginning (T0) and the end (T12) of the streptozotocin treatment (n=6 for each genotype).

Supplemental Table 2.

Probe Set ID	Gene Symbol	Fold-change
1418652_at	Cxcl9	4,9
1419282_at	Ccl12	3,9
1438467_at	Mgl2	3,7
1424727_at	Ccr5	2,9
1450883_a_at	Cd36	2,7
1418204_s_at	Aif1	2,5
1419605_at	Mgl1	2,1
1420380_at	Ccl2	2,0
1446718_at	Nfkbib	0,5
1449731_s_at	Nfkbia	0,4
1418507_s_at	Socs2	0,3
1456532_at	Pdgfd	2,7
1451871_a_at	Ghr	2,0
1417455_at	Tgfb3	1,7
1423136_at	Fgf1	1,7
1449301_at	Slc7a13	13,8
1425559_a_at	Acsm3	8,2
1423828_at	Fasn	2,6
1416022_at	Fabp5	2,5
1430804_at	Slc13a1	2,4
1421821_at	Ldlr	2,1
1434185_at	Acaca	1,8
1437870_at	Slco4c1	1,7
1446552_at	Slc12a3	1,4
1440303_at	Slc7a6	1,4
1450628_at	Slc2a8	0,6

Table S2: Selection of differentially expressed transcripts in kidneys of diabetic WT and *Timp3*$^{-/-}$ mice. Statistical significance was determined by the Limma package of R-Bioconductor (p-value<0.05) The fold change ratio is in linear scale. The fold change threshold was set to 1.5.

Supplemental Table 3.

	Mean Glycemia (mg/dl)	Mean Glycosylated Hb
**Healthy subjects**	97.75 ± 6.6	5.12 ± 0.5
**Diabetic patients**	284 ± 40.4	10.58 ± 1.4

Table S3: Metabolic parameters of healthy subjects and diabetic patients. The table shows the mean ± SD of age, body mass index (BMI), systolic blood pressure (SBP), diastolic blood pressure (DBP), glycemia, glycosylated hemoglobin (HbA1C), soluble creatinine and proteinuria (n=4 for healthy subjects, n=5 for diabetic patients).

Supplemental methods:

qPCR primers

Gene	Primer	Sequence
Timp1	TaqMan	Mm00441818_m1
Timp2	TaqMan	Mm00441825_m1
Timp3	TaqMan	Mm00441827_m1
Timp4	TaqMan	Mm00446568_m1
ADAM 10	TaqMan	Mm00545742_m1
ADAM 15	TaqMan	Mm00477318_m1
ADAM 17	TaqMan	Mm00456428_m1
mir 192	TaqMan	TM 491
mir 216	TaqMan	TM 1944
mir 217	TaqMan	TM 2556
SirT1	TaqMan	Mm00490758_m1
Cd2ap	TaqMan	Mm00815310_s1
PAX2	TaqMan	Mm01217939_m1
Nphs1	TaqMan	Mm00497828_m1
Nphs2	TaqMan	Mm00499929_m1
WT1	TaqMan	Mm00460570_m1
MCP-1	TaqMan	Mm00441242_m1
Mgl-1	TaqMan	Mm00546124_m1
IkBa	TaqMan	Mm00477798_m1
IkBb	TaqMan	Mm00456849_m1
CD36	TaqMan	Mm00432403_m1
SOCS-2	TaqMan	Mm00850544_g1
FoxO1	TaqMan	Mm00490672_m1
FoxO3A	TaqMan	Mm00490673_m1
CCND2	TaqMan	Mm00438070_m1
Cdnk1a	TaqMan	Mm00432448_m1
IGFBP	TaqMan	Mm00833447_m1
GADD45β	TaqMan	Mm00435123_m1
Ucp2	TaqMan	Mm00495907_g1
Beclin1	TaqMan	Mm01265461_m1
Map1LC3a	TaqMan	Mm00458724_m1
Atg5	TaqMan	Mm00504340_m1
Gabarapl1	TaqMan	Mm00457880_m1
STAT1	TaqMan	Mm00439531_m1
Actin	TaqMan	Mm00607939

Supplemental Table 4. List of mouse (A) and human (B) primers used for real time PCR.

SUPPLEMENTAL METHODS

Histological analysis and quantification of renal lesions. Animals were anesthetized and tissues collected for further analysis; right kidney was washed in PBS and fixed in paraformaldehyde for 18-24 hours prior to paraffin-embedding. Four-µm sections of the formalin-fixed, paraffin-embedded kidney tissues were stained with periodic acid-Schiff (PAS) and Masson's trichrome to assess glomerular and tubulointerstitial damage by light microscopy. Sections were evaluated for glomerular sclerosis, as assessed by a standard semi quantitative analysis of 100 glomeruli per animal and expressed as glomerular sclerosis index (GSI). One hundred glomeruli per animal were graded as 0, 1, 2, 3 or 4, according to absent, <25, 25–50, 51–75, or >75% cross-sectional sclerosis, respectively. The GSI for each mouse was calculated by the formula: (N1 x 1 + N2 x 2 + N3 x 3 + N4 x 4) / n, where N1, N2, N3, and N4 represent the numbers of glomeruli exhibiting grades 1, 2, 3, and 4, respectively, and n is the total number of glomeruli assessed (i.e. 100). Glomerular area and mesangial expansion analysis was performed by the use of a custom-made, C-language macro written with the Optimas 6.5 image analysis system (Optimas Corp, MediaCybernetics, Silver Spring, MD). Briefly, the areas of at least 60 glomerular tuft profiles per sample were measured and the harmonic mean of the profile area (mean glomerular area, mGA) was obtained. Then, PAS-positive material in each of these glomeruli was quantified and expressed as percentage of the glomerular tuft area (fractional mesangial area, fMA). The color threshold was set by identifying three to five separate pixels in areas of positive staining. Finally, the mean mesangial area (mMA) was calculated by the formula: (fMA x mGA)/100. For assessment of tubular damage, 20 random fields of renal cortex were examined at a final magnification of 250x. Tubular damage (dilation, atrophy, and interstitial fibrosis) was graded in each field as follows: grade 0, well-preserved renal architecture; grade 1, less than 25% of the field involved; grade 2, 25 to

50% of the field involved; grade 3, 50 to 75% of the field involved; grade 4, 75 to 100% of the field involved.

Human kidney biopsies were collected for diagnostic purpose. Remaining material was fixed in Formalin and Embedded in Paraffin (FFPE) for later use. For IHC, 4μm thick FFPE tissues sections were cut and mounted on microscope slides. TIMP3 expression was tested on 6 different patients' tissues, 4 controls (healthy part of RCC) and 5 affected by Diabetic Nephropathy (DN). Informed consent was obtained from all subjects and all the experiments conformed to the principles set out in the WMA Declaration of Helsinki [http://www.wma.net/en/30publications/10policies/b3/] and the NIH Belmont Report [http://ohsr.od.nih.gov/guidelines/belmont.html].

Briefly, dewaxing and hydration was obtained using an alcohol gradient; heat induced antigen retrieval was performed in citrate buffer pH 6.0; endogenous peroxidases were blocked with H_2O_2 while unspecific binding sites were blocked with protein block (DAKO); TIMP3 antibody was diluted 1:500 in PBS and incubated for 30'. A negative control section was present on each slide. As secondary antibody, Dako REAL™ EnVision™/HRP, Rabbit/Mouse was used and incubated for 30'. Following the addition of the chromogen/substrate solution, cells were stained with Emallume. Slides were mounted and scanned using Aperio's Scan Scope at a 40x magnification.

Quantification of immunohistochemistry. Analogously to PAS positivity, the percentage of glomerular area of positive staining was calculated by means of the image analysis system Optimas™6.5. A region of interest was drawn around the glomerulus. Then, the percentage of positive area for the specific stain (brown color) was calculated at a fixed color threshold (see above). For each kidney specimen, the average for percentage of glomerular area from at least 60 glomeruli was used. For some antigens (F4/80 and MCP-1), positive staining was measured in 20 random fields of the renal cortex examined at a final magnification of 400X and expressed as the mean percentage of fields area

occupied by the specific stain. The antibodies used for immunohistochemistry were: F4/80 (Novus Biologicals, Littleton, CO), CML (Wako Chemicals, Neuss, Germany), NOX4 (SIGMA, St.Louis, MO), Collagen IV (Abcam, Cambridge, UK), RAGE (Novus Biologicals, Littleton, CO), MCP1 (Abcam, Cambridge, UK), FOXO1A (Abcam, Cambridge, UK)
