# Supplementary material for: Loss of TIMP3 underlies diabetic nephropathy via FoxO1/STAT1 interplay
Source: EMBO Mol Med. 2013 Feb 12;5(3):441–55. doi: 10.1002/emmm.201201475 (PMC3598083; doi:10.1002/emmm.201201475)
Supplement: Supplementary file 9 [file emmm0005-0441-SD9.pdf]

Loss of TIMP3 underlies diabetic nephropathy via FoxO1/STAT1 interplay

Loredana Fiorentino, Michele Cavalera, Stefano Menini, Valentina Marchetti, Maria Mavilio, Marta Fabrizi, Francesca Conserva, Viviana Casagrande, Rossella Menghini, Paola Pontrelli, Ivan Arisi, Mara D'Onofrio, Davide Lauro, Rama Khokha, Domenico Accili, Giuseppe Pugliese, Loreto Gesualdo, Renato Lauro and Massimo Federici

Corresponding author: Massimo Federici, University of Rome Tor Vergata

Review timeline:

Submission date:	16 April 2012
Editorial Decision:	02 May 2012
Revision received:	30 July 2012
Editorial Decision:	31 August 2012
Revision received:	18 November 2012
Additional Editorial Correspondence	26 November 2012
Additional Author Correspondence	27 November 2012
Editorial Decision:	06 December 2012
Revision received:	10 December 2012
Accepted:	11 December 2012

Transaction Report:

(Note: With the exception of the correction of typographical or spelling errors that could be a source of ambiguity, letters and reports are not edited. The original formatting of letters and referee reports may not be reflected in this compilation.)

Editors: Céline Carret / Anneke Funk / Roberto Buccione

1st Editorial Decision 02 May 2012

Thank you for the submission of your research manuscript to our editorial offices. It has been seen three referees, and you will find below the last report.

As you will see, this referee is not as positive and raises substantial concerns. In particular protein expression data should be shown, better controls and better images provided where suggested. Importantly, this referee would like to see some indication of autophagy more than just mRNA expression changes of some components.

We would however, give you a chance to revise your manuscript providing that you respond satisfactorily to all issues raised (by all three referees).

Revised manuscripts should be submitted within three months of a request for revision; they will otherwise be treated as new submissions, except under exceptional circumstances in which a short extension is obtained from the editor. Also, the length of the revised manuscript may not exceed 60,000 characters (including spaces) and, including figures, the paper must ultimately fit onto optimally ten pages of the journal. You may consider including any peripheral data (but not methods in their entirety) in the form of Supplementary information.

***** Reviewer's comments *****

Referee #1:

Fiorentino et al report that albuminuria in increased in diabetic Timp3-/- mice. Microarray profiling uncovered a reduction of Foxo1 and FoxO1 target genes expression in diabetic Timp3-/- mice compared to WT, while STAT1, a repressor of FoxO1 transcription, was increased. Re-expression of Timp3 in Timp3-/- mesangial cells rescued the expression of Foxo1 and its targets, and decreased STAT1 expression to control levels. Abolishing STAT1 expression led to a rescue of FoxO1, evoking a role of STAT1 in linking Timp3 deficiency to FoxO1. In kidney biopsies from patients with diabetic nephropathy a reduction was observed in TIMP3, FoxO1 and FoxO1 target genes, while STAT1 expression was increased. The authors conclude that loss of TIMP3 is a hallmark of diabetic kidney disease in human and mouse models and designates TIMP3 as a new possible therapeutic target for diabetic nephropathy. However, there are some major issues that should be solved.

Major issues

1. The title "Loss of TIMP3 underlies diabetic nephropathy and autophagy via FoxO1/STAT1 interplay" is not supported by the data, since autophagy was not studied. It is not enough to show changes in mRNA of genes involved in regulation of autophagy. You have to prove that there is a real autophagy starting with the formation of autophagosome with an immunofluorescence, LC-3 I/LC-3 II ratio...it requires much more in-depth investigations.
2. Protein expression data are missing throughout the entire manuscript (figure 1, figure 3, figure 4, figure 5, figure 6): mRNA expression data should be confirmed at the protein level.
3. Immunohistochemical and histology staining in figure 2 are very poor quality, fig 3b not labeled,
4. Figure 2, figure 3, figure 4 and figure 5 are a potpourri of data that do not follow a thread in the paper and are not cited properly in the manuscript.
5. The discussion is too speculative regarding autophagy, since autophagy is not really studied: gene expression does not mean protein expression and protein expression does not mean that autophagy is taking place
6. The authors refer several times to podocyte functional and molecular dysfunction but they mainly provide mRNA data for whole kidneys. In figure 2 better quality EM should be shown where changes in podocyte morphology may be appreciated and protein data are needed to complement figure 2C.
7. Additional data for the animal model in figure 1 should be shown, such as albuminuria and very importantly, TIMP3 protein data (WB and IH with images from glomeruli and tubules).
8. Figure 1D: we find the purported connection to other parts of the figure too speculative.
9. Why Timp3-/- STZ mice has higher CML expression that WT STZ mice if they have similar glycemia? Also the differences between WT STZ and Timp3-/- STZ in Nitro Tyr staining are not convincing.
10. Non-diabetic TIMP3-/- mice data missing throughout the ms.
11. Non-diabetic TIMP3-/- mice have a basal glucose higher that the WT mice. The differences appear to be statistically significant. Because of this Non-diabetic vehicle-treated TIMP3-/- mice data should be followed similar to the diabetic mice are data shown and compared to vehicle treated WT,
12. It is particularly important to show vehicle treated data for CML since the differences observed for diabetic mice are unaccounted for, but may be explained by higher glucose levels in vehicle treated ko mice.
13. Once again, figure 3 is a mix of genes whose expression is downregulated in the microarray but this information is not enough to support the mechanisms by which Timp3 deficiency may worsen DN. Mgl-1, CD36, SOCS-2 are shown, but the choice of these genes is not well explained in the text, it seems an arbitrary choice of genes.
14. In figure 3B the images should be improved because the decrease in FoxO1 expression in Timp3-/- mice is not clear. Also, the authors should show a series of glomeruli in each figure, in addition to a high power images
15. Again figure 3E is a mixture of downregulated genes such as Cdkn1a, IGFBP1, Ucp2 that do not reappear in the paper and are not introduced or explained. Is there any explanation for CCND2 increases? Why show these genes in the figure but not discuss them?
16. Figure 4: it is unlike that MCP-1 expression does not increases in cells after 48h treatment with

glucose. Also, in Timp3-/- mice in Figure 2F they show MCP-1 increases but not in vitro in T3kd cells with glucose?
17. Why they do not study Atg5 and Beclin1 in figure 4.E as they did in figure 3.E? I do not understand this arbitrary choice...
18. The authors should investigate more deeply the FoxO1 role in ERK, EGFR and AKT phosphorylation. What is the effect on ERK, EGFR and AKT of transfecting the cells with FoxO1 adenovirus?
19. Figure 5 should be better explained. How authors explain that STAT1 expression increases at the protein level but decreases at the mRNA levels? Why STAT1 expression in T3kd + gluc cells decreases in figure 5C but increases in figure 5D when they are compared with T3kd? In figure 5B authors show an increase in STAT1 protein in Timp3-/- diabetic kidneys. STAT1 mRNA and immunohistochemistry expression should be added.
20. Human studies: mRNA data are provided. Protein confirmation is needed
21. Kassiri comparison. The authors speculate in the discussion on the causes of potential differences, but they do not show they tubule data.
22. data showed in figure 6 is not convincing. The author just show selected pictures. They should show a panoramic view where tubules and several glomeruli are appreciated.
23. Figure 6: Also FoxO1 and STAT1 staining is missing.
24. Co-staining with podocyte markers is required to draw any conclusions on TIMP3 staining in podocytes
25. Some clinical information required, such as creatinine and albuminuria levels of the patients
26. Whole kidney RNA studied in humans: cannot draw conclusions on compartment expression

Minor changes

1. Legends for figures are missing (3E, D, 4f) statistics do not correspond to the figure, numbering in legend 4 does not correspond properly with the figure, proper labeling is missing in 3b and 6a.
2. What are the data in RNA, normalized for?
3. Figure 5: Stat1 siRNA, western blot should be shown confirming abolishing of STAT1 expression? Also, siRNA not described in materials and methods section.
4. Osmolarity control for glucose studies.
5. The blot in figure 4.B (p-ERK) is not convincing and should be improved.

Referee #2:

This manuscript describes the role of Adam 17 and Timp3, it inhibitor, as molecular actors of nephropathy. The Timp3 Ko mice rendered diabetic by the use of STZ, are characterized by a reduced foxo1 mRNA concentration along with the corresponding targeted genes whereas conversely Stat1 was augmented. The molecular mechanism controls by Stat1/Timp3 seems to be related to oxidative stress. Several molecular pathways have been characterized supporting their hypothesis. Interestingly, Timp3 gain of function of Stat1 loss of function reversed the molecular phenotypes. Altogether the authors show that impacting Timp3 through Stat1 and Foxo1 regulates the kidney disease. Another important issue is that the authors confirm the reduction of Timp3 in human kidney biopsies further comforting their hypothesis.
This is hence and interesting study that pin point a molecular mechanism of diabetic nephropathy. Several pathways have been incriminated however, this is an observational study at this point and their role still needs to be demonstrated. Conversely, the authors show by multiple approaches that Timp3 seems to be at the cornerstone of the disease. The authors provide some evidences through molecular tools that help supporting the hypothesis however, several issues remain to be addressed.

- The authors have found an increased Adam activity and reduced Timp3 expression. I guess that the processing of proTNFa is impaired. The authors want to check whether TNFa concentration is altered.
- The efficacy of STZ for the induction of type 1 diabetes in mice is not 100%. I guess the authors might have some mice with low levels of hyperglycemia and other high levels, similarly body weight loss might have changed according to the mouse studied. A correlation between index of

type 1 diabetes and Adam 17 and Timp3 and nephropathy would have been interesting to further suggest the causal relationship. The authors nicely described several indexes on page 5 of nephropathy. These should be therefore used to identify relationship with Adam17activity and Timp3 expression.
- The hypothesis of mir217 is interesting, since it would require a full paper to demonstrate this role I would advise to comment the data in the discussion section and suggest the hypothesis only without speculating on the data.
- The distribution of Adam 17 could be different according to the different compartments of the kidney. Although, too tricky to perform the analyses the authors want to comment this point in the discussion section.
- Was the effect of STZ similar on both kidneys? If not was there a relationship between the change in Adam 17 activity and the intensity of the nephropathy?
- The authors have knockdown Timp3 in mesengial cells however they did not provide any effect on TNFa processing or Adam 17 activity. It would be important, through the different genetic approach to have the impact on the shedding enzymes to validate the physiological approach and the specificity of the system.
- In the discussion, the authors would like to discuss why diabetes is reducing Timp3 expression as observed in STZ mice. Is timp3 reduced only after 12 weeks of STZ? Do the authors have experience with shorter time course?

Referee #3 (Comments on Novelty/Model System):

Very nice original paper with a large number of experiments and detailed story.

Referee #3 (Other Remarks):

This paper by Fiorentino and co-authors and entitled: "Loss of TIMP3 underlies diabetic nephropathy and autophagy via FoxO1/STAT1 interplay" describes the role of ADAM17 and its inhibitor TIMP3 in diabetic nephropathy. Diabetic Timp3-/- mice showed increased albuminuria with signs of podocyte and mesangial dysfunction with reduction of Foxo1 (at microarray profiling) and increase in STAT1. Mechanistically, re-expression of Timp3 in Timp3-/- mesangial cells rescued the expression of Foxo1 and its targets, and decreased STAT1 expression to control levels. Finally and more interestingly, studies on kidney biopsies from patients with diabetic nephropathy confirmed a significant reduction in TIMP3, FoxO1 and FoxO1 target genes compared to controls.

Authors ended their study suggesting that loss of TIMP3 is a hallmark of diabetic kidney disease in human and mouse models and designates TIMP3 as a new possible therapeutic target.

This is overall a great work with a large number of experiments performed.

Few minor issues should be addressed, all related to the specific role of Timp3 in diabetic nephropathy:

1) Are all the mechanism of autophagy altered by Timp3 knock down?

2) We are aware of many functions of FoxO1. It will be important to understand if FoxO1 activity is globally or selectively altered. What happen to the FoxO1 targates involved in apoptosis?

3) Did the authors study the expression of Timp3 in other tissues? I am wondering if it is a renal specific issue or a more general thing.

4) By looking at Figure 6 (one of the most important in my view) it looks clear that in diabetic nephropathy there is a downregulation of Timp3. However, the localization is not clear. I am suggesting here to better localize expression by using arrows.

5) I am not 100% sure where Timp3 is localized within human kidney. It will be nice to show one

picture of colocalization of Timp3 and some podocytes (synaptopodin or podocyn) or mesangial markers.

1st Revision - authors' response 30 July 2012

Referee #1:

We thank the Referee for his/her comments. Please find our answers:

Major issues
1-The title "Loss of TIMP3 underlies diabetic nephropathy and autophagy via FoxO1/STAT1 interplay" is not supported by the data, since autophagy was not studied. It is not enough to show changes in mRNA of genes involved in regulation of autophagy. You have to prove that there is a real autophagy starting with the formation of autophagosome with an immunofluorescence, LC-3 I/LC-3 II ratio...it requires much more in-depth investigations.
R: we have now included more data on autophagy in Figure 4D and Figure 6B. In our cell models beside mRNA expression we observed consistent changes in protein expression and the formation of autophagosome (with an immunofluorescence against LC3). However, we propose a new title as follows "Loss of TIMP3 underlies diabetic nephropathy via FoxO1/STAT1 interplay".

2- Protein expression data are missing throughout the entire manuscript (figure 1, figure 3, figure 4, figure 5, figure 6): mRNA expression data should be confirmed at the protein level.
R: we have now included new more data protein expression: see Figures 1D/F, 2D, 6B, 7F.

3. Immunohistochemical and histology staining in figure 2 are very poor quality, fig 3b not labeled,
R: we have now included new data in the Supplemental Information Figures S1, S3, S5-S10, S12-S13, S19-21. We have also tried to improve the quality of the images at the best of our possibility.

4. Figure 2, figure 3, figure 4 and figure 5 are a potpourri of data that do not follow a thread in the paper and are not cited properly in the manuscript.
R: Our aim was to show the line of experiments explaining that TIMP3 may regulate several processes including inflammation (a known story that we did not explored further) and possibly autophagy through FoxO1. We have now re-written the results and re-arranged the figure to follow a thread in the manuscript, according to reviewer suggestions.

5. The discussion is too speculative regarding autophagy, since autophagy is not really studied: gene expression does not mean protein expression and protein expression does not mean that autophagy is taking place
R: we have limited the discussion on autophagy to the evidence that we show now in the manuscript.

6. The authors refer several times to podocyte functional and molecular dysfunction but they mainly provide mRNA data for whole kidneys. In figure 2 better quality EM should be shown where changes in podocyte morphology may be appreciated and protein data are needed to complement figure 2C.
R: we have now included new data from EM (see Figure 2A). We have tried to complement with protein data the expression of protein podocyte markers but the antibodies did not work properly possibly because of the limited expression of these proteins in the whole kidney homogenate that we had available. Unfortunately due to restricted time for revision we were unable to start a new protocol to freshly isolate glomeruli. We accepted this limitation in the discussion (Page 15 lines 25-28).

7. Additional data for the animal model in figure 1 should be shown, such as albuminuria and very importantly, TIMP3 protein data (WB and IH with images from glomeruli and tubules).
R: we not show for TIMP3 both WB and IHC data in Figure 1, Albuminuria from diabetic mice was already reported in the previous Figure 2D and we now extended the analysis that is shown in Figure 2C of the revised version.

8. Figure 1D: we find the purported connection to other parts of the figure too speculative.
R: according also to the other reviewers we eliminated this connection from the data.

9. Why Timp3-/- STZ mice has higher CML expression that WT STZ mice if they have similar glycemia? Also the differences between WT STZ and Timp3-/- STZ in Nitro Tyr staining are not convincing.

R: Nε-(Carboxymethyl)lysine (CML) is an advanced glycation end product formed on protein by combined nonenzymatic glycation and oxidation (glycoxidation) reactions. CML is also formed during lipid oxidation in the presence of protein. Thus, it is better defined as a glyco and lipoxidation endproduct (Fu et al. J Biol Chem. 26; 271:9982-6, 1996). Augmented production of CML can depend on either increased levels of the substrates (glucose and/or lipids) or oxidative stress. The finding that, in addition to the increase of CML, Nitrotyrosine and Nox4 were also increased in glomeruli of diabetic Timp3 $^{-/-}$ vs the corresponding WT mice, suggests that higher CML levels should be considered as marker of oxidative stress rather than a measure of plasma glucose concentration. In our experimental setting, increased oxidative stress can be generated by both higher glucose and increased inflammation. As Timp3$^{-/-}$ and WT diabetic mice have similar glycemic values, increased levels of glomerular CML in diabetic mice depend on higher local ROS production.

10. Non-diabetic TIMP3-/- mice data missing throughout the ms.
11. Non-diabetic TIMP3-/- mice have a basal glucose higher that the WT mice. The differences appear to be statistically significant. Because of this Non-diabetic vehicle-treated TIMP3-/- mice data should be followed similar to the diabetic mice are data shown and compared to vehicle treated WT,
R to both points: We have now included the data from non-diabetic vehicle treated WT and TIMP3-/- mice for most of IH and WB and PCR throughout the manuscript. As a control we used only non-diabetic vehicle treated WT and TIMP3-/-.

12. It is particularly important to show vehicle treated data for CML since the differences observed for diabetic mice are unaccounted for, but may be explained by higher glucose levels in vehicle treated ko mice.
Data are now shown in Figure S8.
Circulating AGE-peptides, included CML, are reabsorbed at the tubular level (Gugliucci et al. Diabetologia. 39:149-60, 1996). As a result, while tubular staining essentially depends on circulating levels of CML, staining of glomerular structures, such as mesangium and glomerular basal membranes (see images at 1000X), reflects the rate of local CML generation through the above described mechanisms (point #9). This is the reason why we measured CML staining only at glomerular level. Thus, as stated above, differences in glomerular level of CML observed between Timp3 $^{-/-}$ and WT diabetic mice are accounted for by the differences in renal inflammation and oxidative stress observed between the two genotypes. This interpretation is supported by the observation that, despite the higher basal levels of glucose in Timp3 $^{-/-}$ mice, there was no increase in glomerular CML staining, or in nitrotyrosine, Nox4 and inflammatory markers. A mild increase in CML staining observed in the tubular compartment of Timp3$^{-/-}$ vs WT mice was noticed but not reported, as well as was not reported the increased tubular staining in Timp3$^{-/-}$ vs WT diabetic mice.

13. Once again, figure 3 is a mix of genes whose expression is down regulated in the microarray but this information is not enough to support the mechanisms by which Timp3 deficiency may worsen DN. Mgl-1, CD36, SOCS-2 are shown, but the choice of these genes is not well explained in the text, it seems an arbitrary choice of genes.
R: the choice of genes to be shown was related to underline that loss of TIMP3 through the increased activities of MMPs and ADAMs might raise inflammation, cell proliferation/fibrosis and other processes. We have now clarified better this concept stating from page 6 lines 25 to page 7 lines 11) that we identifies some genes belonging to different gene clusters to validate microarray results by PCR, specifically those markers that we found modulated in the "inflammation", "cell proliferation", "transcription factor" clusters. Since the data may diverge the attention from the main focus of the manuscript that is on TIMP3/FoxO1/autophagy, we elected to move the validation data in Supplementary Figure S11.

14. In figure 3B the images should be improved because the decrease in FoxO1 expression in Timp3-/- mice is not clear. Also, the authors should show a series of glomeruli in each figure, in addition to a high power images

R: we have now included several more images to show that FoxO1 is both decreased and nuclear excluded in TIMP3-/- mice in the different compartments of the kidney. See figure 3 and Figures S12, S13.

15. Again figure 3E is a mixture of down regulated genes such as Cdkn1a, IGFBP1, Ucp2 that do not reappear in the paper and are not introduced or explained. Is there any explanation for CCND2 increases? Why show these genes in the figure but not discuss them?

R: these genes were shown because they were found down regulated in the microarray and are targets of FoxO1. The validation PCR data were shown to support the concept of a specific FoxO1 defect in our model. However since the data may diverge the attention from the main focus of the manuscript that is on TIMP3/FoxO1/autophagy, we elected to move the validation data in Supplementary Figure S14, together with some other FoxO1 targets not modulated to support the idea that some but not all the targets of FoxO1 are deregulated in our model.

16. Figure 4: it is unlike that MCP-1 expression does not increases in cells after 48h treatment with glucose. Also, in Timp3-/- mice in Figure 2F they show MCP-1 increases but not in vitro in T3kd cells with glucose?

R: the difference may be explained by the duration of hyperglycemia in vivo (12 weeks) in comparison to in vitro (48h). Moreover, the in vivo results report the expression of MCP-1 from different sources rather than one cell type as in vitro.

17. Why they do not study Atg5 and Beclin1 in figure 4.E as they did in figure 3.E? I do not understand this arbitrary choice...

R: these data are now included in Figures 3E, 4C, 6A, 6B, 8A and S17.

18. The authors should investigate more deeply the FoxO1 role in ERK, EGFR and AKT phosphorylation. What is the effect on ERK, EGFR and AKT of transfecting the cells with FoxO1 adenovirus?

R: we have performed the signaling studies suggested. For space reason we could not include this experiment in the manuscript but we are sending the data for the referee 1 (see Figure for Referee 1 only).

For referee 1 only

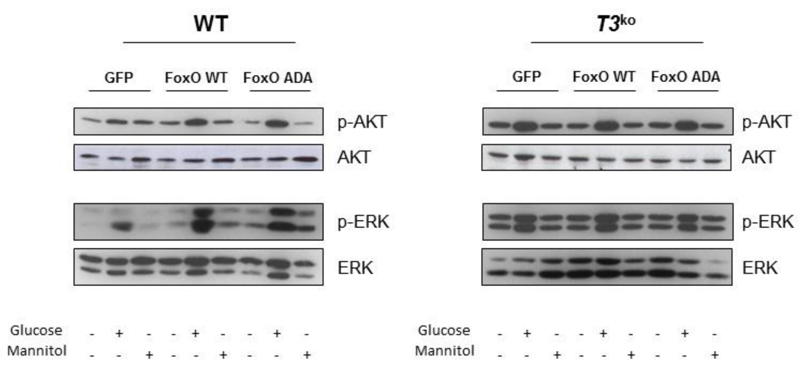

Effect of FoxO1 WT or FoxO1 ADA overexpression on AKT and ERK signaling pathways in WT and Timp3KO primary mesangial cells

19. Figure 5 should be better explained. How authors explain that STAT1 expression increases at the protein level but decreases at the mRNA levels? Why STAT1 expression in T3kd + gluc cells decreases in figure 5C but increases in figure 5D when they are compared with T3kd? In figure 5B

authors show an increase in STAT1 protein in Timp3-/- diabetic kidneys. STAT1 mRNA and immunohistochemistry expression should be added.
R: the apparent controversy was due to the fact that the mRNA expression in previous figures 5C/D was related to different controls. However we have now elected to show only the data on primary mesangial cells, reported in Figure 7D,E were we show the effect of TIMP3 reconstitution on both STAT1 RNA and Protein levels. We included all other STAT1 mRNA, protein and immunohistochemistry from cell and animal models in Figure 7F-h and S19.

20. Human studies: mRNA data are provided. Protein confirmation is needed
R: we could only provide immunostaining confirmation since the fine needle biopsy material is not enough to extract protein for western blots. We accepted this limitation in the discussion (page 15 lines 19-24).

21. Kassiri comparison. The authors speculate in the discussion on the causes of potential differences, but they do not show they tubule data.
R: the data are shown now for both mice and humans in Figures 1C, S1, 8B-E. Despite some intensity differences between mice and humans our data support the concept that TIMP3 is down regulated in all the kidney compartments in diabetic conditions. However, the glomerular down regulation appeared to us more significant than the tubular down regulation and this is supported by Woroniecka et al, Diabetes 2011.

22. data showed in figure 6 is not convincing. The author just show selected pictures. They should show a panoramic view where tubules and several glomeruli are appreciated.
R: we show the images as requested now in Figure 8B.

23. Figure 6: Also FoxO1 and STAT1 staining is missing.
R: we show the images as requested now in Figure S20 and S21.

24. Co-staining with podocyte markers is required to draw any conclusions on TIMP3 staining in podocytes
R: we show the images as requested now in Figure 8D,E. As we comment in the discussion, being TIMP3 an extracellular protein it is conceivable that its reduction affects more than one cell type, including podocyte (page 15 lines 19-24).

25. Some clinical information required, such as creatinine and albuminuria levels of the patients
R: we show the images as requested now in Supplementary Table 3.

26. Whole kidney RNA studied in humans: cannot draw conclusions on compartment expression
R: we included this limitation in the discussion (page 15 lines 19-24).

Minor changes

1. Legends for figures are missing (3E, D, 4f) statistics do not correspond to the figure, numbering in legend 4 does not correspond properly with the figure, proper labeling is missing in 3b and 6a.
R: we improved the quality of legends and figures as suggested.

2. What are the data in RNA, normalized for?
R: the RNA data are normalized for actin (mouse) and 18S (human) as reported in the methods.

3. Figure 5: Stat1 siRNA, western blot should be shown confirming abolishing of STAT1 expression? Also, siRNA not described in materials and methods section.
R: we show not that STAT1 expression is abolished in Figure 7F and siRNA are now described.

4. Osmolarity control for glucose studies.
R: data are included as requested in Figures 4A-C, S16A, S18A,B, 6A, 6B.

5. The blot in figure 4.B (p-ERK) is not convincing and should be improved.
R: the blot has been improved and it is no in Figure S16B.

Referee #2:

We thank the referee for his/her positive comments. We have included new results to answer his questions as follows

- The authors have found an increased Adam activity and reduced Timp3 expression. I guess that the processing of proTNFa is impaired. The authors want to check whether TNFa concentration is altered.

R: we show in Figure 1E-G increased ADAM17 activity, elevated circulating TNFa and increased shedding of ProTNF-a in tissue homogenates from diabetic and non diabetic TIMP3-/- compared to diabetic and non diabetic WT.

- The efficacy of STZ for the induction of type 1 diabetes in mice is not 100%. I guess the authors might have some mice with low levels of hyperglycemia and other high levels, similarly body weight loss might have changed according to the mouse studied. A correlation between index of type 1 diabetes and Adam 17 and Timp3 and nephropathy would have been interesting to further suggest the causal. The authors nicely described several indexes on page 5 of nephropathy. These should be therefore used to identify relationship with Adam17 activity and Timp3 expression.

R: Upon your suggestion we re-analyzed the mice that did not responded to STZ (so called STZ Low Glucose in the manuscript). As shown in Figure S2B-H, in these mice we found overall not significant increases in indexes of kidney damage compared to control.

- The hypothesis of mir217 is interesting, since it would require a full paper to demonstrate this role I would advise to comment the data in the discussion section and suggest the hypothesis only without speculating on the data.

R: according to the other reviewers we eliminated this point which deserves more study.

- The distribution of Adam 17 could be different according to the different compartments of the kidney. Although, too tricky to perform the analyses the authors want to comment this point in the discussion section.

R: we have commented this point in the discussion at page 15 lines 19-24.

- Was the effect of STZ similar on both kidneys? If not was there a relationship between the change in Adam 17 activity and the intensity of the nephropathy?

R: as shown in Figure S2A there is no difference between dx and sx

- The authors have knockdown Timp3 in mesengial cells however they did not provide any effect on TNFa processing or Adam 17 activity. It would be important, through the different genetic approach to have the impact on the shedding enzymes to validate the physiological approach and the specificity of the system.

R: the effect of TIMP3 knockout in mesangial cells is shown in Figure S18A,B

- In the discussion, the authors would like to discuss why diabetes is reducing Timp3 expression as observed in STZ mice. Is timp3 reduced only after 12 weeks of STZ? Do the authors have experience with shorter time course?

R: we have commented this point in the discussion at page 14 lines 4-6. Unfortunately, to limit the number of mice for ethical reasons we only analyzed this time point that was suggested to be relevant for kidney nephropathy by analysis of the literature.

Referee #3 (Comments on Novelty/Model System):

We thank the referee for his/her positive comments. We have included new results to answer his questions as follows

Few minor issues should be addressed, all related to the specific role of Timp3 in diabetic nephropathy:

1) Are all the mechanism of autophagy altered by Timp3 knock down?

R: in the microarray analysis only some ATG genes were deregulated; since autophagy is a complex process requiring different players we both validated the microarray results but also investigated some other potential markers, which we excluded from further analysis because of negative data, now reported in Figure S15.

2) We are aware of many functions of FoxO1. It will be important to understand if FoxO1 activity is globally or selectively altered. What happen to the FoxO1 targates involved in apoptosis?

R: The effect of TIMP3 on FoxO1 seems to be specific for some pathways regulating autophagy, possibly as a consequence of the level of nuclear exclusion which is associated to the loss of TIMP3. For instance expression of apoptosis targets such as BiM and FasL is not modulated in diabetic TIMP3-/-, now shown in Figure S14.

3) Did the authors study the expression of Timp3 in other tissues? I am wondering if it is a renal specific issue or a more general thing.

R: it is not a general thing since for instance the expression is increased in the liver and unchanged in WAT.

Figure for referee 3 only.

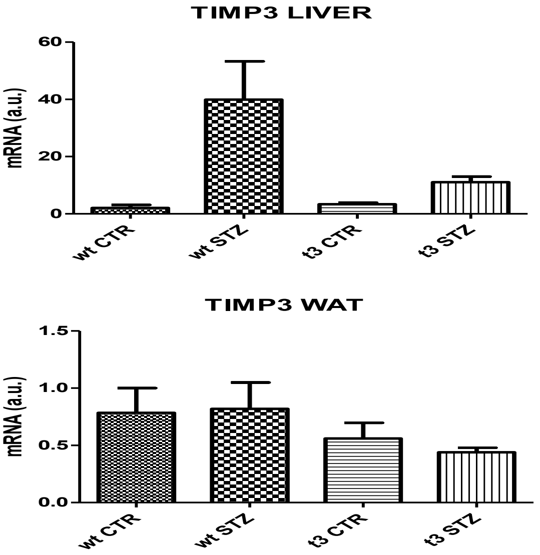

4) By looking at Figure 6 (one of the most important in my view) it looks clear that in diabetic nephropathy there is a down regulation of Timp3. However, the localization is not clear. I am suggesting here to better localize expression by using arrows.

R: the data are now in Figure 8B with arrows to indicate TIMP3 expression which is present both in glomeruli and tubules although in the diabetic specimens the reduction of Timp3 seems to be higher in the glomeruli compartment.

5) I am not 100% sure where Timp3 is localized within human kidney. It will be nice to show one picture of colocalization of Timp3 and some podocytes (synaptopodin or podocyn) or mesangial markers.

R: the data are now shown in Figure 8D,E using Synaptopodin as comarker. From the immunofluorescence shown it appears that TIMP3 is deposited in the extracellular matrix potentially affecting not only podocyte but also both mesangial and endothelial cells. In particular, Synaptopodin immunolabelling (green) highlights podocyte foot processes and is characterized by thin linear staining along the surface of the glomerular basement membranes (GBM) of capillary loops. Timp3 immunoreactivity (red) is mainly observed in the podocyte cell body (*) and primary processes (white arrows).

2nd Editorial Decision 31 August 2012

Thank you for the submission of your revised manuscript "Loss of TIMP3 underlies diabetic nephropathy via FoxO1/STAT1 interplay" to EMBO Molecular Medicine and please accept my apologies for the delayed reply. We have now received the enclosed reports from the referees whom we asked to re-assess it.

As you will see, the Reviewers acknowledge that the manuscript was significantly improved during revision and Reviewer #2 indicates that it is suitable for publication. However, Reviewer #1 raises significant concerns about the conclusiveness of the newly added results. Since we do acknowledge the potential interest of your findings, we would be willing to consider a revised manuscript with the understanding that the referee concerns must be convincingly and conclusively addressed.

Importantly, Reviewer #1 points out that it remains unclear whether autophagy is indeed impaired under high glucose conditions in T# KD cells (point 2).
Revised manuscripts should be submitted within three months of a request for revision; they will otherwise be treated as new submissions, unless arranged differently with the editor.

I look forward to seeing a revised form of your manuscript as soon as possible.

***** Reviewer's comments *****

Referee #1:

The authors have answered many of my questions. However, some of the new data raise new questions, as for example, the controls added to figure 2.B. Furthermore the studies regarding autophagocytosis and podocytes should be improved since the new figures provide contradictory information.
1. Last lines page 4. "We identify a similar cooperation between human TIMP3, FoxO1 and autophagy in renal biopsies from patients with diabetic nephropathy". However, autophagy was not studied in human biopsies.
2. LC3 studies are unconvincing.
"The glucose-induced autophagosome formation process visualized through LC3 redistribution inside cells was greatly impaired in T3kd MES cells compared to control MES (Fig 4D)." The figure shows increased LC3 staining in glucose treated WT cells vs WT and less staining in glucose T3kd MES. However the LC3 immunofluorescence is not convincing. No redistribution is observed. The figure is low clarity and I cannot appreciate autophagosomes (for an example see the Ab datasheet: http://www.cellsignal.com/products/4108.html).
Furthermore, these immunofluorescence results are discordant with Western blot results. In Western blot, and using the same antibody, LC3 staining is decreased in cells cultured in high glucose, both in WT, T3kd and T3kdTt3. These results do not suggest "glucose-induced autophagosome formation". Furthermore in the Western blot I cannot appreciate the conversion of LC3 to the lower migrating form LC3-II, as indicator of autophagy. Also it would be good to have a control that efficiently induce autophagy. Again, mRNA data is not enough as indicator of autophagy.
3. Figure 2.b. We thank the authors for providing controls for this figure. However, when examining the new controls provided one can clearly appreciate that, despite the authors write about podocyte molecular dysfunction, figure 2B shows that he pattern of podocyte mRNA expression in STZ TIMP3 KO mice is "normal", that is, undistinguishable from TIMPO3 KO non-diabetic and from wt non-diabetic. In the absence of protein studies the significance of these findings is unclear. Thus, the sentence "Electron microscopy analysis of STZ-Timp3-/- kidney showed morphological and molecular alterations suggestive of podocyte dysfunction, including increased basal membrane thickness, signs of severe foot processes effacement (Fig 2A) and decreased mRNA expression of podocyte markers Cd2ap, Pax2, Nephrin, Podocin and WT1 (Fig 2B)," should be modified. In addition, since STZ diabetics have albuminuria and increased mRNA expression, it is clear these mRNAs are unrelated to albuminuria: they are increased in WT STZ with pathological albuminuria, but are normal in TIMP3KO STZ with even more albuminuria. Again, protein studies are required.

Referee #2 (Comments on Novelty/Model System):

I think the originality of the animal model and the large number of data are strongly supporting the hypothesis and demonstrating the mechanisms. it looks that the authors did a huge effort to provide many more data.

Referee #2 (Other Remarks):

I thank the authors for all efforts performed to gather all additional data required by the reviewers including myself

2nd Revision - authors' response 18 November 2012

Point to point answer to Editor and Reviewers

First of all we would like to thank you and the reviewers for the positive and constructive comments, which allowed us to improve our manuscript. With the help of these comments, we hope that this revised version meets the stringent criteria of interest for your readers.

New Experiments incorporated in the second revised version
Following the useful suggestions of the referees, we performed several new experiments and show further results, now incorporated in the second revised version:

1. Protein levels for ATG5/ATG8/LC3/Beclin in cellular models described in Figure 4, including mRNA and protein levels in cells cultured under osmotic control (mannitol) and serum deprivation (positive control).
2. Calculation of LC3II/LC3I ratio in cellular models described in Figure 4.
3. Incorporation of new immunofluorescence confocal images showing LC3 redistribution under high glucose culture conditions and using serum starvation as positive control in cellular models described in Figure 4, also including a digital zoom as a separate Supporting Information S16.
4. A new image of LC3 protein western blot analysis in cellular models described in Figure 6.
5. Calculation of LC3II/LC3I ratio in cellular models described in Figure 6.
6. Incorporation of new images showing LC3 redistribution under high glucose culture conditions and using starvation as positive control in cellular models described in Figure 6, also including a digital zoom as a separate Supporting Information S20.
7. we also tried at our best to improve the quality of the immunoflorescence figures. However, for the reviewer use, we also included raw data figures regarding Figure 4E, 6C, S20.

Reviewer #1

We wish to thank the Reviewer #1 for her/his constructive and positive comments.

Major comments:

1. *Last lines page 4. "We identify a similar cooperation between human TIMP3, FoxO1 and autophagy in renal biopsies from patients with diabetic nephropathy". However, autophagy was not studied in human biopsies.*

OUR ANSWER: we agree with the reviewer. The sentence was modified into “We identify similar changes in expression of human TIMP3 and FoxO1 in renal biopsies from patients with diabetic nephropathy”.

2. LC3 studies are unconvincing.
"The glucose-induced autophagosome formation process visualized through LC3 redistribution inside cells was greatly impaired in T3kd MES cells compared to control MES (Fig 4D)." The figure

shows increased LC3 staining in glucose treated WT cells vs WT and less staining in glucose T3kd MES. However the LC3 immunofluorescence is not convincing. No redistribution is observed. The figure is low clarity and I cannot appreciate autophagosomes (for an example see the Ab datasheet: http://www.cellsignal.com/products/4108.html).
OUR ANSWER: we have now had the opportunity to repeat the immunofluorescence studies with the Ab suggested by the R1. The new images are shown in Figure 4 and Supporting Information Figure S16, Figure 6 and Supporting Information Figure S20; the Supporting Figures show increased digital zoom. We believe that the redistribution of LC3 can now be easily appreciated. In particular in Figure 4 and S16, LC3 redistribution is evident in Ctrl MES13 under high glucose condition but is lacking in $T3^{kd}$ MES13. A similar pattern is shown in cells under serum starvation included as a positive control.
Finally, we also uploaded the raw data for immunofluorescence studies in cells (Figure 4E, Figure 6C, S20) for the reviewer use.

Furthermore, these immunofluorescence results are discordant with Western blot results. In Western blot, and using the same antibody, LC3 staining is decreased in cells cultured in high glucose, both in WT, T3kd and T3kdTt3. These results do not suggest "glucose-induced autophagosome formation". Furthermore in the Western blot I cannot appreciate the conversion of LC3 to the lower migrating form LC3-II, as indicator of autophagy. Also it would be good to have a control that efficiently induce autophagy. Again, mRNA data is not enough as indicator of autophagy.
OUR ANSWER: we repeated our western blot analysis of LC3 protein using the Ab suggested by the R1 both in MES13 (cell line) and pMes (primary cells) systems (Figure 4 and 6 respectively), and the results obtained are in agreement with the IF studies. We have also calculated LC3II/I ratio (as an indicator of autophagy activation) which support the results of a different regulation of autophagy in cells lacking *Timp3* compared to the controls. Moreover, we also acquired new confocal images (Figure 6 and Supporting Information S20) in which LC3 redistribution is rescued in $\mathit{Timp3}^{ko}$ pMes cells under high glucose when TIMP3 is reintroduced through an adenovirus system, while the introduction of GFP does not obtain the same effect; serum starvation, used as positive control, showed the same result.

Figure 2.b. We thank the authors for providing controls for this figure. However, when examining the new controls provided one can clearly appreciate that, despite the authors write about podocyte molecular dysfunction, figure 2B shows that he pattern of podocyte mRNA expression in STZ TIMP3 KO mice is "normal", that is, undistinguishable from TIMPO3 KO non-diabetic and from wt non-diabetic. In the absence of protein studies the significance of these findings is unclear. Thus, the sentence "Electron microscopy analysis of STZ-Timp3-/- kidney showed morphological and molecular alterations suggestive of podocyte dysfunction, including increased basal membrane thickness, signs of severe foot processes effacement (Fig 2A) and decreased mRNA expression of podocyte markers Cd2ap, Pax2, Nephrin, Podocin and WT1 (Fig 2B)," should be modified. In addition, since STZ diabetics have albuminuria and increased mRNA expression, it is clear these mRNAs are unrelated to albuminuria: they are increased in WT STZ with pathological albuminuria, but are normal in TIMP3KO STZ with even more albuminuria. Again, protein studies are required.
OUR ANSWER: we agree with the reviewers that these data deserve further investigation which goes beyond the scope of this study. For these reasons we have removed the data from the second revised version, limiting the observation to increased basal membrane thickness and albuminuria.

Reviewer #2

We wish to thank the Reviewer #2 for her/his constructive and positive comments.

Additional Editorial Correspondence 26 November 2012

This is to let you know that we have now received the comments from the two Reviewers whom we asked to evaluate your revised manuscript.

I would like to offer you the opportunity to respond to this Reviewer's comments:

"The authors have acted on the issues raised. However, I am seriously concerned about the changing nature of the data. As an example, in figure 6B of the previous version it is clearly observed that LC3 expression is decreased under high glucose conditions. The authors have changed this blot and now report that LC3 is increased under high glucose conditions, even though the antibody used is the same. However, the actin bands are the same in both versions of the manuscript. Thus, I am not confident that the actin belongs to the same blot that is now shown in figure 6B, and I am very puzzled by these different results."

I look forward to hearing from you as soon as possible with a detailed response to these comments.

Additional Author Decision 27 November 2012

Please find below our response to R1 comments.
Dr. Fiorentino from my lab prepared a powerpoint figure reporting the new and old figure 6B and the control loading for the new WB:

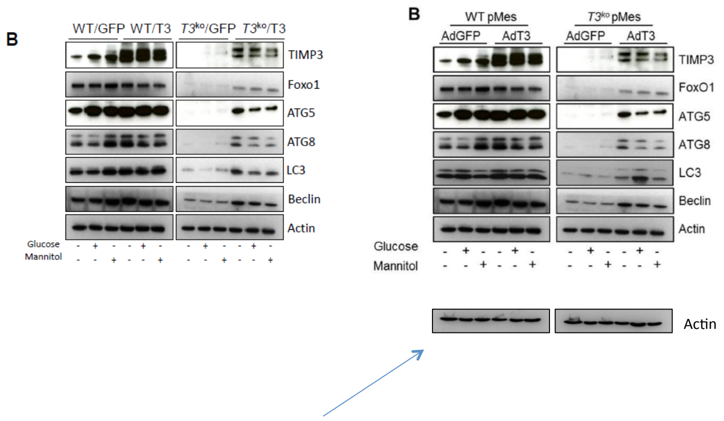

This actin blot was used to control the blot with the new LC3 ab.
We maintained the previous actin since it is representative for the other blots.

Comments for the editor:

"The authors have acted on the issues raised. However, I am seriously concerned about the changing nature of the data. As an example, in figure 6B of the previous version it is clearly observed that LC3 expression is decreased under high glucose conditions. The authors have changed this blot and now report that LC3 is increased under high glucose conditions, even though the antibody used is the same. However, the actin bands are the same in both versions of the manuscript. Thus, I am not confident that the actin belongs to the same blot that is now shown in figure 6B, and I am very puzzled by these different results."

Our answer: in the latest version of fig.6B, the SAME samples shown in the previous version of the figure were run on a 15% gel to allow a better separation between the two forms of LC3. The LC3 antibody used for the WB was the NEW one suggested by the referee (as clearly stated in the point by point response) and not the one used in the previous version of the figure. We did not change the panel relative to the actin loading control because it refers to all the other blots of the figure; however, we did check for equal loading also the 15% gel, and we are attaching the relative figure.

The nature of the data has not changed and the results shown are not different: we simply used the new antibody, which was better than the previous one and allowed a better recognition of the LC3 bands. Since the new antibody recognizes both LC3 bands the quality of the new blot is markedly improved.

The use of the new antibody was requested by R1 and clearly stated in our answer and in the methods.

The new blot in figure 6B confirms that in our model 1) in the absence of TIMP3 there is a deficit in autophagy, 2) there is no reporting that high glucose increases autophagy but instead 3) we show the evidence that under high glucose conditions if you rescue TIMP3 expression you rescue autophagy as observed by different approaches and different methods.

2nd Editorial Decision 06 December 2012

Thank you for the submission of your revised manuscript to EMBO Molecular Medicine and also for providing a commentary and the files and source data to solve the remaining issues raised by Reviewer 1.

We have carefully examined and discussed your new version, comments and data. I should state beforehand that, as mentioned by Reviewer 1, Fig.6B as presented, is not formally acceptable and appropriate . However, we are fully convinced that the new LC3 data shown in Figure 6B were obtained using the same samples but running them on a gel with different polyacrilamide concentration to allow better band separation and using the new antibody suggested by Reviewer 1. We also note that the quantification shown in the histogram in panel 6B was performed using the appropriate loading controls shown in your provided source data.

We also acknowledge your comments that activation of autophagy is not the central point pf your work, but rather that TIMP3 reduction primes the diabetic kidney with reduced ability to use autophagy proteins if needed as a consequence of other processes.

For these reasons, I am pleased to inform you that we will be able to accept your manuscript pending the following final amendments: please separate the LC3 blot panel in Fig.6B by associating it with the appropriate actin loading control and explaining in the figure legend that those particular samples were run using a different gel concentration. Also please clearly state the exact antibodies used throughout your work (by citing code numbers) in the Materials and Methods section.

Finally, also make sure that the instructions listed below are carefully adhered to (where applicable).

In general, we are now starting to encourage the publication of source data, particularly for electrophoretic gels and blots, with the aim of making primary data more accessible and transparent to the reader. Would you be willing to provide a PDF file per figure that contains the original, uncropped and unprocessed scans of all or key gels used in the figure? The PDF files should be labeled with the appropriate figure/panel number, and should have molecular weight markers; further annotation may be useful but is not essential. The PDF files will be published online with the article as supplementary "Source Data" files. If you cannot do this, we could at least do so for Figure 6, if you provide us with the corresponding PDF file. If you have any questions regarding this just contact me.

Please submit your revised manuscript as soon as possible and in any case within two weeks. I look forward to seeing a revised form of your manuscript as soon as possible.

3rd Revision - authors' response 10 December 2012

First of all we would like to thank you and your colleagues in the editorial office to have thoroughly evaluated our comments to Reviewer 1.

We apologize to have not paid attention to the composition of the Figure 6B in the V3 version.
Please find a new version of Figure 6 in which we have separated the LC3 western from the others. We also included separate actin loading control as suggested. The legend for Figure 6B has been accordingly corrected and we specified that LC3 blot was performed on a 15% gel (see page 32 lines 1-3).
We have now included the specifics for the antibodies used for the western blots in the material and methods section (see page 17 lines 2-9).
As a secondary note we have re-uploaded also Figure 1 since in the V3 version the word tubulin was missing from the label in Figure 1D lower panel blot (control loading).

A PDF reporting the uncropped/unprocessed scans for principal gels showed in Figure 1 to 8 has been included as a supporting file.

We hope that our manuscript may be now acceptable for *EMBO MOLECULAR MEDICINE*.
